# miR-199a-5p Is Upregulated during Fibrogenic Response to Tissue Injury and Mediates TGFbeta-Induced Lung Fibroblast Activation by Targeting Caveolin-1

**DOI:** 10.1371/journal.pgen.1003291

**Published:** 2013-02-14

**Authors:** Christian Lacks Lino Cardenas, Imène Sarah Henaoui, Elisabeth Courcot, Christoph Roderburg, Christelle Cauffiez, Sébastien Aubert, Marie-Christine Copin, Benoit Wallaert, François Glowacki, Edmone Dewaeles, Jadranka Milosevic, Julien Maurizio, John Tedrow, Brice Marcet, Jean-Marc Lo-Guidice, Naftali Kaminski, Pascal Barbry, Tom Luedde, Michael Perrais, Bernard Mari, Nicolas Pottier

**Affiliations:** 1 EA4483, Faculté de Médecine de Lille, Pole Recherche, Lille, France; 2 Centre National de la Recherche Scientifique, Institut de Pharmacologie Moléculaire et Cellulaire, UMR-7275, Valbonne Sophia-Antipolis, France; 3 Université de Nice Sophia-Antipolis, Nice, France; 4 Department of Medicine III, University Hospital RWTH Aachen, Aachen, Germany; 5 Institut National de la Santé et de la Recherche Médicale, U837, Jean-Pierre Aubert Research Center, Equipe 5 “Mucines, Différentiation et Cancérogenèse Épithéliales”, Lille, France; 6 Pôle de Pathologie, CHRU Lille, Lille, France; 7 Faculté de Médecine, Université de Lille 2, Lille, France; 8 Service de Pneumologie et Immunoallergologie, CHRU Lille, Lille, France; 9 Dorothy P. and Richard P. Simmons Center for Interstitial Lung Disease, Division of Pulmonary, Allergy, and Critical Care Medicine, University of Pittsburgh School of Medicine, Pittsburgh, Pennsylvania, United States of America; Centre for Cancer Biology, SA Pathology, Australia

## Abstract

As miRNAs are associated with normal cellular processes, deregulation of miRNAs is thought to play a causative role in many complex diseases. Nevertheless, the precise contribution of miRNAs in fibrotic lung diseases, especially the idiopathic form (IPF), remains poorly understood. Given the poor response rate of IPF patients to current therapy, new insights into the pathogenic mechanisms controlling lung fibroblasts activation, the key cell type driving the fibrogenic process, are essential to develop new therapeutic strategies for this devastating disease. To identify miRNAs with potential roles in lung fibrogenesis, we performed a genome-wide assessment of miRNA expression in lungs from two different mouse strains known for their distinct susceptibility to develop lung fibrosis after bleomycin exposure. This led to the identification of miR-199a-5p as the best miRNA candidate associated with bleomycin response. Importantly, miR-199a-5p pulmonary expression was also significantly increased in IPF patients (94 IPF versus 83 controls). In particular, levels of miR-199a-5p were selectively increased in myofibroblasts from injured mouse lungs and fibroblastic foci, a histologic feature associated with IPF. Therefore, miR-199a-5p profibrotic effects were further investigated in cultured lung fibroblasts: miR-199a-5p expression was induced upon TGFβ exposure, and ectopic expression of miR-199a-5p was sufficient to promote the pathogenic activation of pulmonary fibroblasts including proliferation, migration, invasion, and differentiation into myofibroblasts. In addition, we demonstrated that miR-199a-5p is a key effector of TGFβ signaling in lung fibroblasts by regulating CAV1, a critical mediator of pulmonary fibrosis. Remarkably, aberrant expression of miR-199a-5p was also found in unilateral ureteral obstruction mouse model of kidney fibrosis, as well as in both bile duct ligation and CCl_4_-induced mouse models of liver fibrosis, suggesting that dysregulation of miR-199a-5p represents a general mechanism contributing to the fibrotic process. MiR-199a-5p thus behaves as a major regulator of tissue fibrosis with therapeutic potency to treat fibroproliferative diseases.

## Author Summary

Fibrosis is the final common pathway in virtually all forms of chronic organ failure, including lung, liver, and kidney, and is a leading cause of morbidity and mortality worldwide. Fibrosis results from the excessive activity of fibroblasts, in particular a differentiated form known as myofibroblast that is responsible for the excessive and persistent accumulation of scar tissue and ultimately organ failure. Idiopathic Lung Fibrosis (IPF) is a chronic and often rapidly fatal pulmonary disorder of unknown origin characterized by fibrosis of the supporting framework (interstitium) of the lungs. Given the poor prognosis of IPF patients, new insights into the biology of (myo)fibroblasts is of major interest to develop new therapeutics aimed at reducing (myo)fibroblast activity to slow or even reverse disease progression, thereby preserving organ function and prolonging life. MicroRNAs (miRNAs), a class of non-coding RNA recently identified, are associated with normal cellular processes; and deregulation of miRNAs plays a causative role in a vast array of complex diseases. In this study, we identified a particular miRNA: miR-199a-5p that governs lung fibroblast activation and ultimately lung fibrosis. Overall we showed that miR-199a-5p is a major regulator of fibrosis with strong therapeutic potency to treat fibroproliferative diseases such as IPF.

## Introduction

Tissue fibrosis, defined as the excessive and persistent formation of non functional scar tissue in response to repeated injury and insult, is a leading cause of morbidity and mortality associated with organ failure in various chronic diseases such as those affecting the lung interstitium [1]. Among the interstitial lung diseases of unknown etiology, Idiopathic Pulmonary Fibrosis (IPF) is the most common and lethal with a median survival of 3 to 5 years after diagnosis [2]. The pathogenesis of IPF is complex and largely unknown [2], but observations based on both animal models of pulmonary fibrosis and lung sections from patients with IPF suggest a dynamic pathobiological process involving excessive wound healing with chronic inflammation, apoptosis of epithelial and endothelial cells, mesenchymal cell proliferation and activation with the formation of fibroblasts/myofibroblasts foci, and finally excessive deposition of extracellular matrix resulting in the destruction of the lung architecture and the loss of lung functions [2]. In particular, myofibroblasts play a substantial role in IPF by secreting important amount of ECM components and by promoting lung tissue stiffening [3]. Given the poor response rate of IPF patients to current therapy, a detailed understanding of the underlying pathogenic mechanisms is of major interest to develop new effective therapeutic strategies targeting the cellular and molecular events involved in the fibrotic response.

MicroRNAs (miRNAs) are a class of noncoding small RNA, which most often bind to the 3′ UTR of target genes mRNAs and thereby repress their translation and/or induce their degradation. Since the first miRNA identification in *Caenorhabditis elegans* in a context of larval development [4], [5], thousands miRNAs have now been characterized including about 2000 in human (miRbase v19) [6]. MiRNAs are now recognized as major regulators of gene expression with crucial functions in numerous biological processes including development, proliferation, differentiation, apoptosis and stress response. Importantly, recent studies have identified specific miRNA expression patterns related to the initiation and progression of various diseases including cancer as well as inflammatory, infectious and autoimmune diseases [7]–[9]. Additionally, gain and loss of function miRNA studies have further established their functional impact in various *in vivo* models [10]–[15]. Nevertheless, the precise contribution of miRNAs in fibrotic diseases, especially lung fibrosis, is still poorly understood [16], [17]. Our rationale was therefore to test whether miRNAs may provide new perspectives on disease mechanisms, diagnosis as well as new therapeutic opportunities in the specific context of fibrosis.

In an effort to identify miRNAs with potential roles in the development of lung fibrosis (strategy detailed in Figure S1), we aimed to identify miRNAs of interest in two mouse strains showing different susceptibility to develop lung fibrosis after bleomycin exposure. This led to the identification of a panel of miRNAs specifically dysregulated in the lungs of fibrosis prone mouse strain in response to bleomycin. Among these miRNAs, miR-199a-5p was found to be selectively up-regulated in myofibroblasts of the injured lung in bleomycin-treated mice and fibroblastic foci of IPF patients. In lung fibroblasts, miR-199a-5p acts as an effector of TGFβ signaling, regulates CAV1 expression, a critical mediator of the lung fibrosis process [18]–[21] and participates to multiple fibrogenic associated-processes including cell proliferation, migration, invasion and differentiation into myofibroblasts. Finally, dysregulation of miR-199a-5p was also found in two other mouse models of tissue fibrosis, namely kidney fibrosis and liver fibrosis, suggesting therefore that miR-199a-5p is likely to be a common mediator of fibrosis.

## Results

### Fibrosis-sensitive and -resistant mice exhibit a distinct miRNA expression profile in response to bleomycin

**Figure 1:**
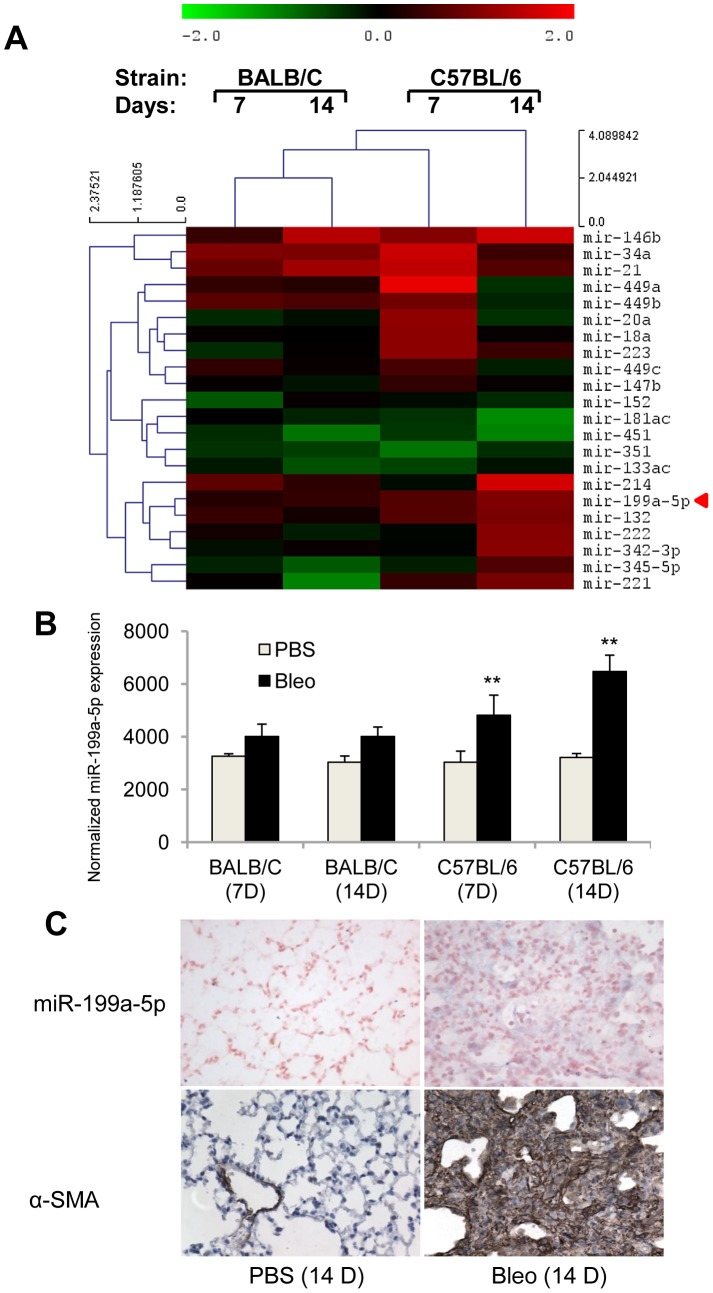
miR-199a-5p expression during bleomycin induced lung fibrosis. (A) Heat map representing the statistically significant (adjusted p-value<0.05) differentially expressed microRNAs in lungs from BALB/C and C57BL/6 mice in response to bleomycin at the indicated time points. Up-regulated microRNAs are shown in progressively brighter shades of red, depending on the fold difference, and down-regulated microRNAs are shown in progressively brighter shades of green. miR-199a-5p is marked in red. n = 3 mice in each group. (B) miR-199a-5p expression in lungs from BALB/C and C57BL/6 mice in response to bleomycin at the indicated time points. n = 3 mice in each group. Data from microarrays experiments are expressed as mean of normalized fluorescence intensity ± SEM. **p<0.01 (C) Paraffin sections were prepared from lungs of C57BL/6 mice 14 days following bleomycin intra-tracheal instillation. *In situ* hybridization and immunohistochemistry assays were performed to determine the colocalization of miR-199a-5p and α-SMA. Results represent one out of three independently performed experiments.

Previous studies based on mice have demonstrated a genetic susceptibility to bleomycin-induced pulmonary fibrosis [22], [23]. Indeed, C57BL/6 mice are considered to be fibrosis prone, whereas BALB/C mice are less prone to fibrosis. To identify miRNAs that may contribute to the lung fibrosis process, miRNA expression profile in response to bleomycin was assessed 7 days and 14 days following bleomycin administration (*i.e.* when active fibrogenesis occurs) on both strains using a microarray based platform (Data set 1, GEO accession number GSE34812) described elsewhere [24]–[26]. We identified 22 differentially expressed miRNAs between lungs from bleomycin- and control-treated animals in at least one strain, the majority being upregulated in bleomycin-instillated lungs (Figure 1A). We focused our analysis on miRNAs that exhibited an enhanced expression in response to bleomycin during disease progression in the C57BL/6 sensitive mice only. Among several miRNAs candidates with such a profile, miR-199a-5p displayed the highest statistical score (Figure 1B). This was further established using an independent set of mice at day 14 following bleomycin treatment (Figure S2A). These findings strongly suggested that miR-199a-5p may play an important role during the lung fibrosis process. To investigate the regulatory mechanisms underlying miR-199a-5p production, we assessed the expression status of the 2 mouse genes, miR-199a-1 (on chromosome 9) and miR-199a-2 (on chromosome 1) in response to bleomycin using a Taqman assay designed to discriminate between pri-miR-199a-1 and pri-miR-199a-2. Our results showed that, 14 days after bleomycin instillation, both pri-miR-199a transcripts were up-regulated in the lungs of C57BL/6 mice (Figure S2B) and thus, contributed to miR-199a-5p production. In addition, *in situ* hybridization experiments performed in the injured lungs from C57BL/6 mice 14 days after bleomycin instillation revealed a selective expression of miR-199a-5p in myofibroblasts (Figure 1C).

Of note, consistent with previous findings [14], we also found a significant upregulation of miR-21 (now referenced in miRbase as mmu-miR-21a-5p) in response to bleomycin (Figure 1A and Figure S3). Nevertheless, miR-21 induction did not differ between bleomycin-sensitive and bleomycin-resistant strains of mice.

### Identification of miR-199a-5p target genes in lung fibroblasts

**Figure 2:**
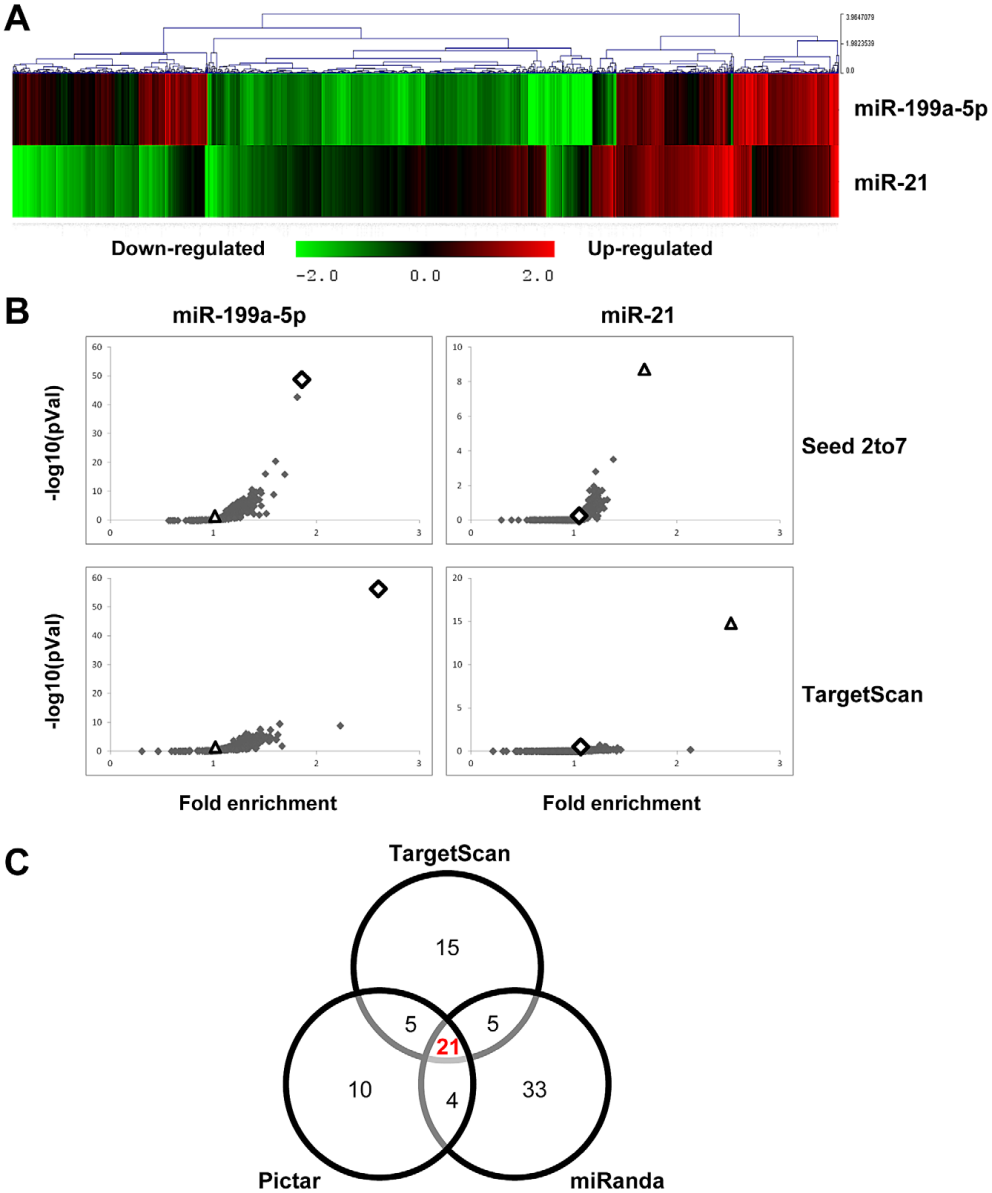
Identification of miR-199a-5p candidate targets using a transcriptomic approach. Normal human pulmonary fibroblasts hFL1 were transfected with pre-miR-Neg, pre-miR-199a-5p or pre-miR-21 (n = 2). RNA samples were harvested at 48 h post-transfection and expression profiles were determined with pan genomic arrays. (A) Heatmap comparing the normalized log_2_ of the ratios between the signal in the different conditions and the pre-miR-Neg signal. (B) Overrepresentation of miRNA predicted targets in the set of down-regulated transcripts following miR-199a-5p and miR-21 transfection using the webtool miRonTop. Graphs show the significance of the enrichment, represented as −log_10_ (adjusted p-value), according to the fold enrichment using 2 different prediction tools for all known miRNAs: miR-199a-5p and miR-21 are represented as an open diamond and triangle, respectively. Threshold values used to define the set of up- and down-regulated genes: AveExp = 7.0; log FC = 0.7; adjusted p-value = 0.05. (C) Venn diagram comparing the number of miR-199a-5p targets among the set of highly-downregulated genes following pre-miR-199a-5p transfection according to 3 distinct target prediction tools. Cut-offs for selection are equal to 7.0 for the log_2_ (signal), to −1.5 for the log_2_ (ratio), and to 0.01 for the adjusted p-value.

We next sought to determine the mechanism by which miR-199a-5p dysregulation may lead to tissue fibrosis. To address this question, we first attempted to identify gene targets and cellular pathways regulated by miR-199a-5p using the methodology described earlier [25], [26]. The influence of miR-199a-5p on human pulmonary hFL1 fibroblast transcriptome was compared with that of miR-21, which has been previously associated with the development of fibrotic diseases including lung fibrosis [14], [15], [27] (Data set 2, GEO accession number GSE34815). Forty-eight hours after ectopic overexpression of each miRNA, a significant alteration (defined by an absolute log_2_ratio above 0.7 and an adjusted p-value below 0.05) of 1261 and 753 transcripts was detected in the miR-199a-5p and miR-21 conditions, respectively. While these 2 miRNAs induced very distinct gene expression patterns (Figure 2A), a functional annotation of these signatures, using Ingenuity Pathway software, indicated an overlap for “canonical pathways” including “Cell Cycle regulation” and “TGFβ Signaling” (Table S1). Consistent with previous findings [28], highly significant pathways associated with miR-21 were related to “Cyclins and Cell Cycle Regulation” as well as “Cell Cycle Control of Chromosomal Replication”, “Mismatch Repair in Eukaryotes” and “ATM signalling”. While the highest scoring pathway for miR-199a-5p corresponded to the metabolic pathways “Biosynthesis of Steroids”, we also noticed enrichment for pathways related to “Integrin Signaling” and “Caveolar-mediated Endocytosis Signaling”. We next looked for an enrichment of putative direct targets in the population of down-regulated transcripts, as described in [29]. A specific overrepresentation of predicted targets for miR-199a-5p and miR-21 in the population of down-regulated transcripts was noticed after heterologous expression of either miR-199a-5p or miR-21, respectively. This enrichment was independent of the prediction tool used to define the targets (Figure 2B and not shown). We then focussed our analysis on a subset of 21 transcripts containing miR-199a-5p complementary hexamers in their 3′UTR, showing the largest inhibition of expression, and identified by TargetScan, PicTar and miRanda (Figure 2C and Table 1). The gene list of interest was further narrowed by focussing on targets also associated with the most significant canonical pathways described above. Interestingly, the expression levels of 4 out of 21 mouse orthologs were also significantly down-regulated in C57BL/6 mice 14 days after instillation of bleomycin (Data set 3, GEO accession number GSE34814, Table S2). These targets, highlighted in Table 1, are *ARHGAP12*, *CAV1*, *MAP3K11* and *MPP5*. Based on previous studies that demonstrated a significant link between the downregulation of caveolin-1 (CAV1) in lung fibroblasts and the deleterious effects mediated by TGFβ [19], [30], *CAV1* represented a particularly relevant putative miR-199a-5p target gene.

**Table 1:** List of the main miR-199a-5p predicted targets significantly downregulated following miR-199-5p overexpression in human fibroblasts using the bioinformatics tool miRonTop (http://www.microarray.fr:8080/miRonTop/index).

Symbol	Accession Number	Description	Av.Exp^1^	logFC^2^
ARHGAP12^3^	NM_018287	Rho GTPase activating protein 12	11.12	−1.96
C17orf63	NM_018182	-	11.90	−1.58
CAV1^3^	NM_001753	Caveolin 1, caveolae protein, 22 kDa (CAV1)	15.28	−1.78
DDR1	NM_013993	Discoidin domain receptor tyrosine kinase 1	9.57	−2.31
EPB41L1	NM_012156	Erythrocyte membrane protein band 4.1-like 1	12.88	−1.94
EXTL3	NM_001440	Exostoses (multiple)-like 3	13.66	−1.20
FZD6	NM_003506	Frizzled homolog 6	11.62	−2.07
IPO8	NM_006390	Importin 8	12.00	−2.09
KLHL3	NM_017415	Kelch-like 3	8.44	−1.32
KPNA4	NM_002268	Karyopherin alpha 4	13.44	−1.11
MAP3K11^3^	NM_002419	Mitogen−activated protein kinase kinase kinase 11	13.11	−2.77
MPP5^3^	NM_022474	Membrane protein, palmitoylated 5	11.76	−1.99
NLK	NM_016231	Nemo-like kinase	11.36	−1.63
PODXL	NM_001018111	Podocalyxin-like (PODXL)	11.21	−2.59
PPP1R2	NM_006241	Protein phosphatase 1, regulatory (inhibitor) subunit 2	11.29	−2.50
PXN	NM_002859	Paxillin	14.99	−1.41
RNF11	NM_014372	Ring finger protein 11	14.30	−1.09
ST6GAL1	NM_173216	ST6 beta-galactosamide alpha-2,6-sialyltranferase 1	10.67	−1.70
TAF9B	NM_015975	TAF9B RNA polymerase II, TATA box binding protein (TBP)-associated factor, 31 kDa	9.77	−2.25
VPS26A	NM_004896	Vacuolar protein sorting 26 homolog A	12.37	−1.60
ZNF706	NM_001042510	Zinc finger protein 706	8.15	−1.10

1 logarithm (base 2) of the average intensity (AveExpr).

2 logarithm (base 2) of the ratio of miR-199a-5p/miR-Neg (logFC).

3 Expression significantly down-regulated in bleomycin-treated C57BL/6 mice.

### CAV1 is a *bona fide* miR-199a-5p target

**Figure 3:**
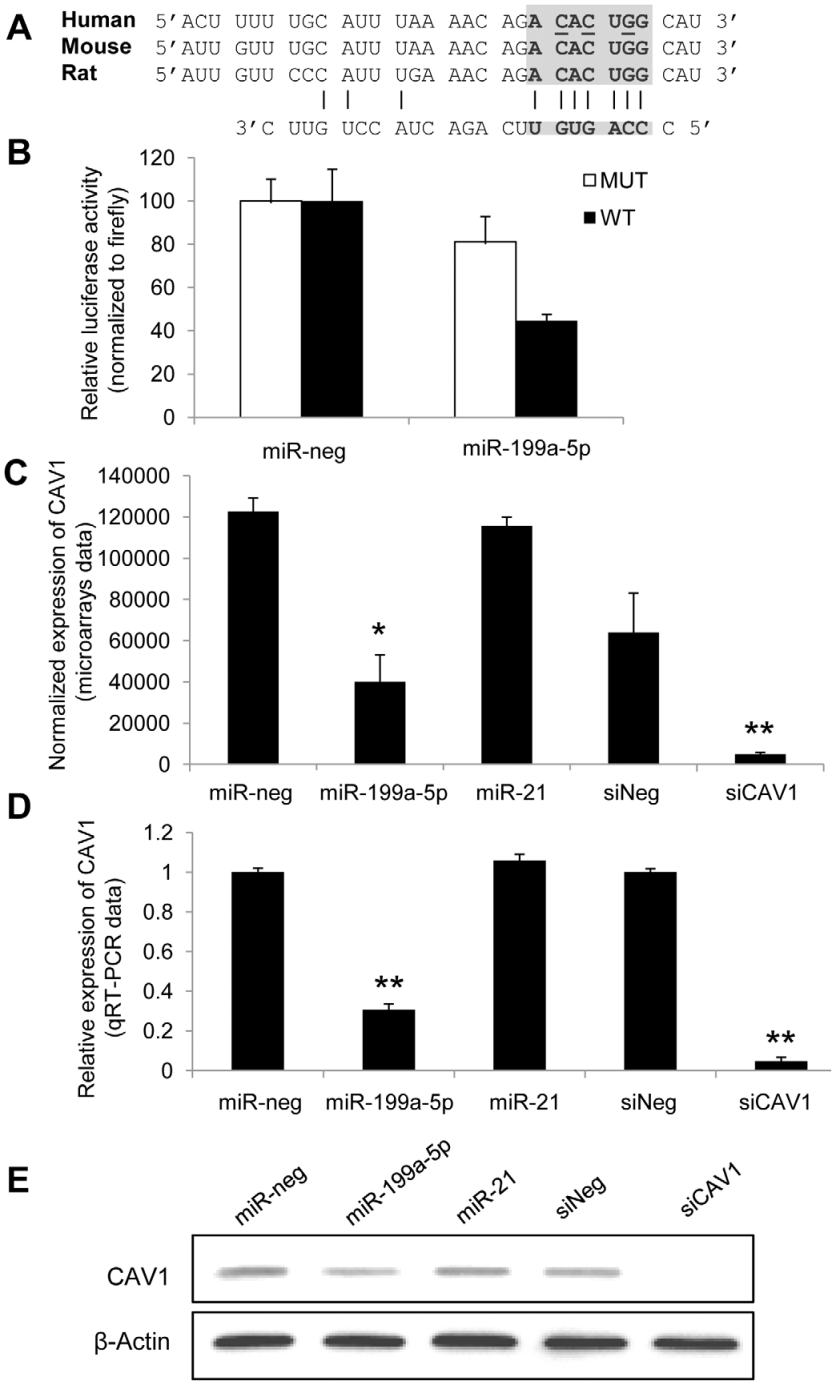
CAV1 is a direct target of miR-199a-5p. (A) Position of miR-199a-5p target site in CAV1 3′ UTR and sequence alignment of miR-199a-5p and the CAV1 3′ UTR from various species are shown. The “seed” region with a conserved WC match to the eighth nucleotide of the miRNA is highlighted. Bases that have been mutated in the psiCHECK-2 construct are underlined. (B) Co-transfection of pre-miR-199a-5p or pre-miR-Neg and human CAV1 3′UTR-derived psiCHECK-2 construct (wild type or mutated in the putative miR-199a-5p seed region) in HEK293 cells. Cells were harvested two days after transfection and luciferase activities were analyzed. All renilla luciferase activities were normalized with firefly luciferase activity. * p<0.05. (C) and (D) Normalized expression values of CAV1 in hFL1 lung fibroblasts after transfection with pre-miR-199a-5p, pre-miR-21 or si-CAV1 from microarrays (C, n = 2) or real time PCR (D, n = 3) experiments. Data are expressed as mean ± SEM (n = 2). **p<0.01 (E) Western blot analysis showing the down regulated expression of CAV1 protein after transfection of hFL1 lung fibroblasts with pre-miR-199a-5p, pre-miR-21 or si-CAV1. One representative experiment out of two is shown.

Alignment of miR-199a-5p with human CAV1 3′UTR sequence revealed one potential conserved seed site (Figure 3A). We then fused part of the human CAV1 3′UTR to a luciferase reporter using the psiCHEK-2 vector and transfected it into HEK293 cells in the presence of either a pre-miR-199a-5p mimic or a pre-miR-control (Figure 3B). As a control, we also used a CAV1 3′UTR construct mutated on the predicted miR-199a-5p site. Human pre-miR-199a-5p induced a significant decrease in the normalized luciferase activity relative to control in the presence of the wild type construction only, confirming that it represents a functional site. Moreover, this inhibition was also repeated using the whole 3′-UTR of human CAV1 (Figure S4), demonstrating that *CAV1* is indeed a direct target of miR-199a-5p. Finally, transfection of pre-miR-199a-5p into MRC-5 and hFL1 lung fibroblasts led to a significant and specific decrease of CAV1 at both mRNA and protein levels while miR-21 had no significant effect (Figure 3C–3E and Figure S5).

### miR-199a-5p mediates TGFβ-induced downregulation of CAV1 in pulmonary fibroblasts

**Figure 4:**
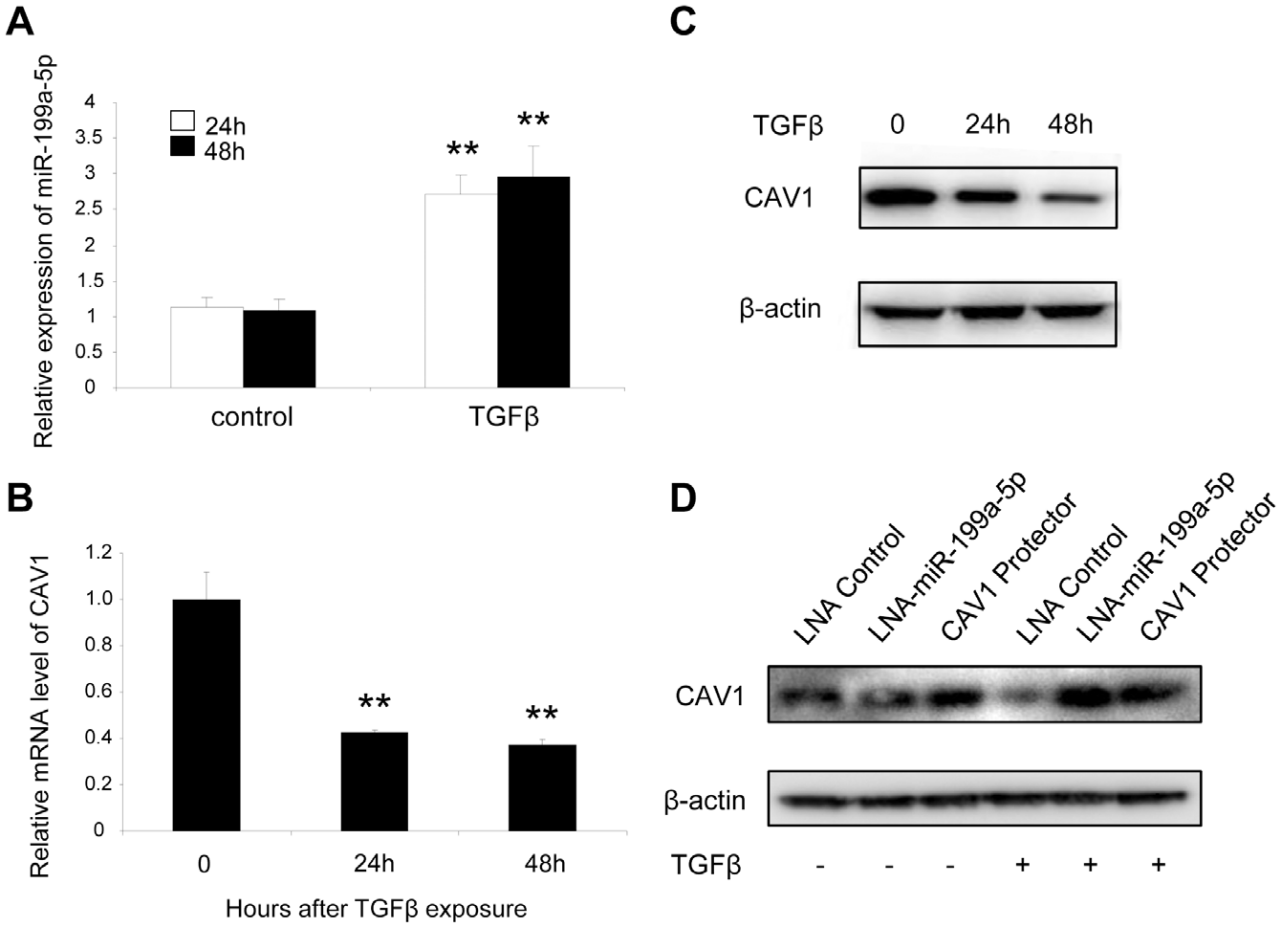
TGFβ regulates CAV1 by increasing miR-199a-5p expression. MRC-5 lung fibroblasts were treated with 10 ng/mL TGFβ for 24 h and 48 h. MiR-199a-5p (A) and CAV1 expression levels (B) were determined by Taqman PCR. Data are expressed as mean ± SEM. ** p<0.01. (C) CAV1 protein levels were determined by immunoblot analysis. (D) MRC5 cells were transfected with LNA-miR-199a-5p, CAV1 protector or LNA-control then incubated with or without 10 ng/ml TGFβ for 24 h and CAV1 protein levels were determined by immunoblot analysis. Data are representative of three independent experiments.

As TGFβ is known to downregulate CAV1 in pulmonary fibroblasts [19], we then investigated whether decreased expression of CAV1 upon TGFβ stimulation was associated with an increase in miR-199a-5p expression. We exposed the MRC-5 cell line to TGFβ, and analyzed the expression levels of CAV1 and miR-199a-5p. As detected by Taqman RT-PCR, TGFβ treatment of human fibroblasts for 24 h or 48 h caused a marked decrease of CAV1 mRNA, whereas miR-199a-5p expression was significantly upregulated (Figure 4A and 4B). Decrease of CAV1 protein levels after TGFβ treatment was time dependent (Figure 4C). To further investigate whether miR-199a-5p is involved in TGFβ-induced downregulation of CAV1, we performed additional experiments using a LNA-based inhibitor of miR-199a-5p as well as a CAV1 target site blocker to specifically interfere with miR-199a-5p binding on CAV1 3′UTR. As depicted in Figure 4D and S6, both LNA-mediated silencing of miR-199a-5p and blocking miR-199a-5p binding on CAV1 3′UTR inhibit TGFβ-induced downregulation of CAV1. Altogether, these experiments demonstrate that, in lung fibroblasts, induction of miR-199a-5p in response to TGFβ mediates CAV1 downregulation through binding on a unique site located in CAV1 3′UTR.

### Altered expression of CAV1 in the lungs of bleomycin induced pulmonary fibrosis mice

**Figure 5:**
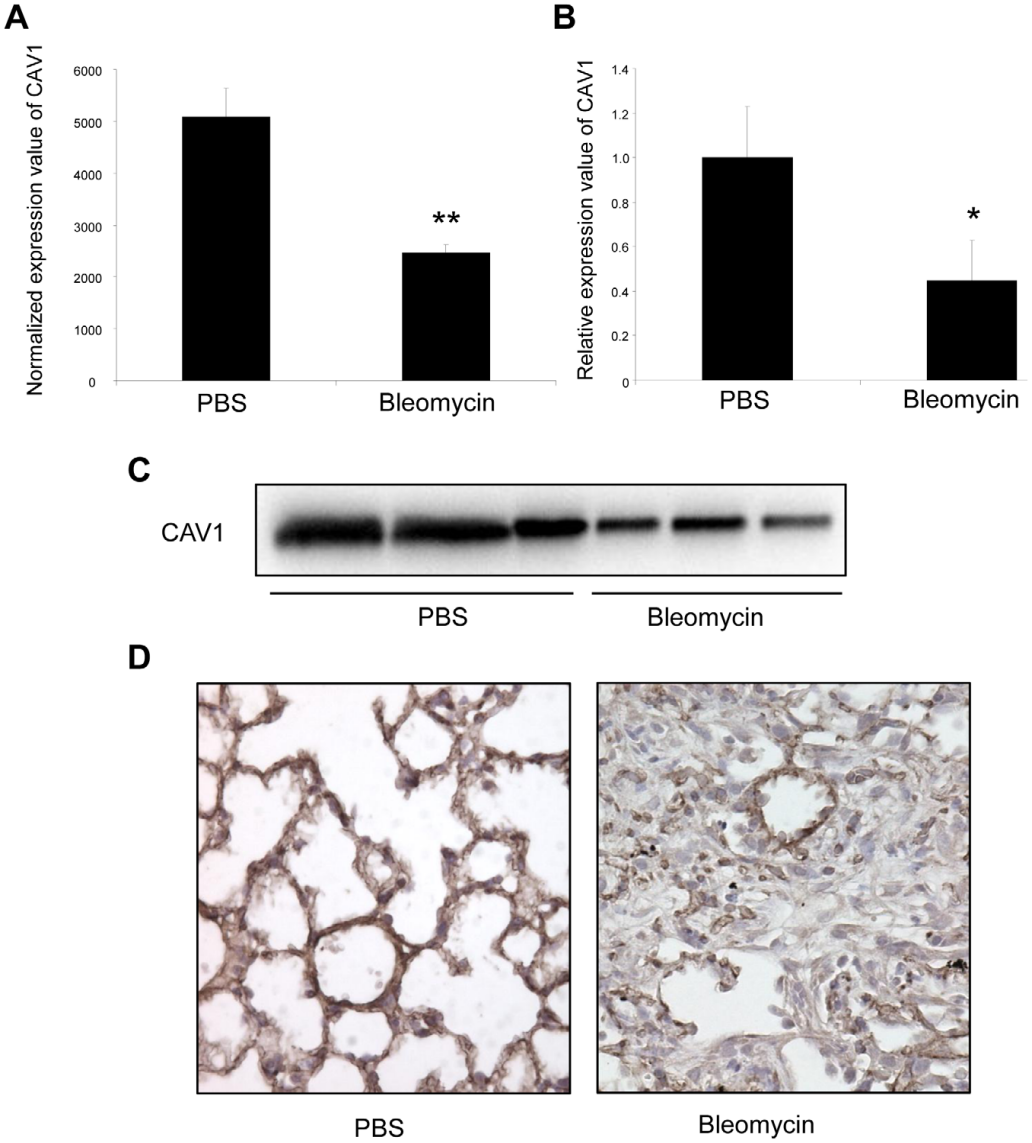
Altered CAV1 expression in bleomycin induced lung fibrosis mouse model. (A) Microarray analysis reveals a significant reduction of CAV1 expression in C57BL/6 mice treated with bleomycin for 14 days compared with control mice. Data are expressed as mean normalized fluorescence values ± SEM (n = 5). ** p<0.01. (B) Real-time PCR was performed to confirm the reduced expression of CAV1 in lungs of C57BL/6 mice 14 days following bleomycin exposure. Data are expressed as mean ± SEM (n = 5). * p<0.05. (C) CAV1 protein expression as detected by immunoblot analysis in lung tissue samples from C57BL/6 mice treated with bleomycin for 14 days and control mice (n = 3). (D) Immunohistochemical analysis of CAV1 expression in lung tissue sections from C57BL/6 mice 14 days after bleomycin intra-tracheal instillation. One representative section out of three is shown.

We then assessed the expression of CAV1 in the fibrotic lungs of mice. Consistent with previous studies [19], [31], our data showed a significant decrease in both CAV1 mRNA and protein expression levels in C57Bl/6 mice 14 days after bleomycin administration (Figure 5A–5C). Additionally, immunohistochemistry staining of CAV1 on lung tissue sections from C57Bl/6 mice 14 days after bleomycin treatment revealed a marked reduction of CAV1 in fibrotic area of the lungs (Figure 5D). Taken together, these experiments show that the observed up-regulation of miR-199a-5p expression in the fibrotic lungs of mice is correlated with a downregulation of CAV1. Of note, BALB/c mice, for which pulmonary expression of miR-199a-5p was not upregulated in response to bleomycin, did not display a significant decrease in CAV1 mRNA expression level 14 days after bleomycin treatment (Figure S7).

### Concomitant altered expression of both CAV1 and miR-199a-5p in lungs of IPF patients

**Figure 6:**
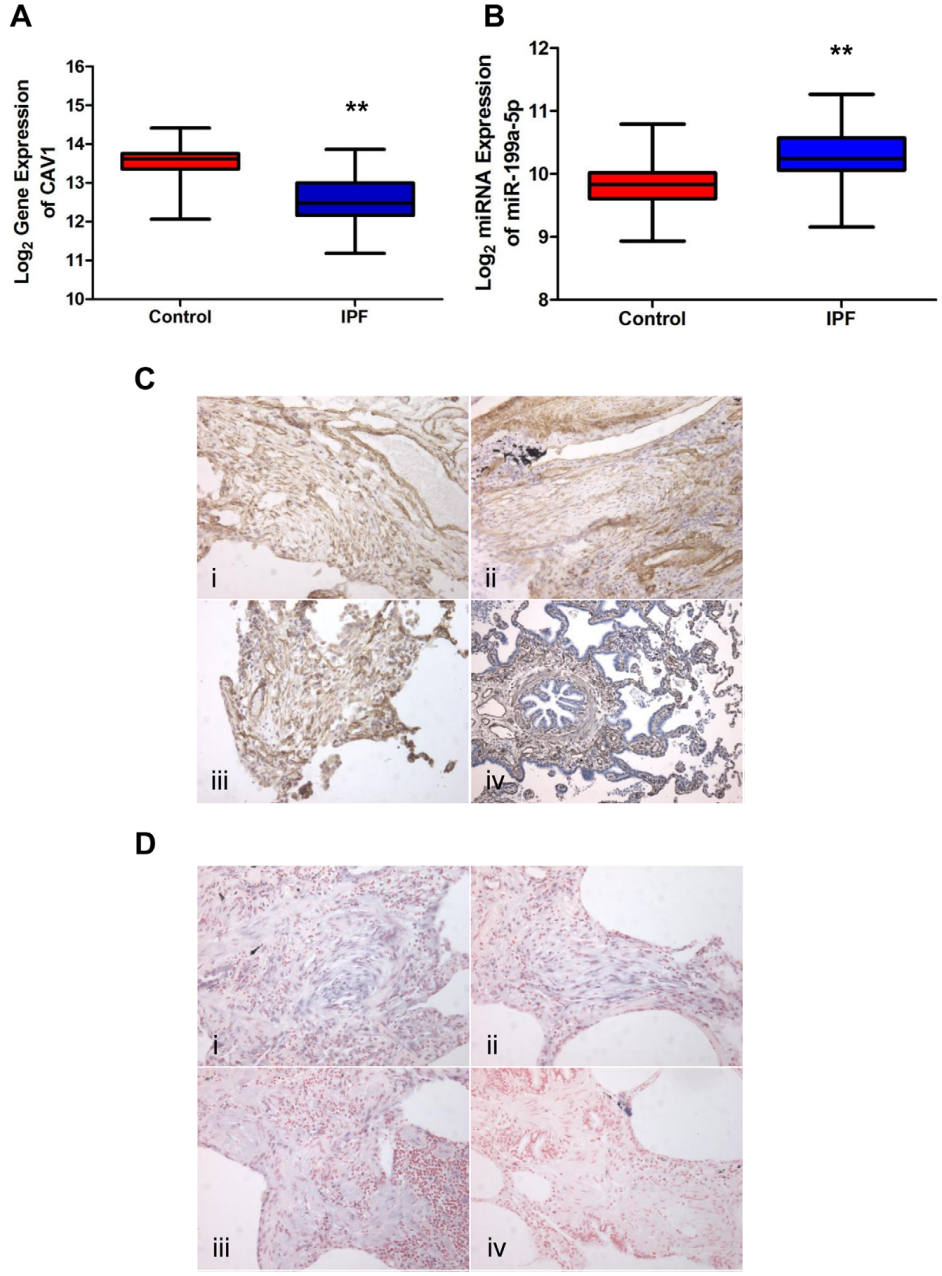
miR-199a-5p and its target CAV1 are dysregulated in IPF. (A,B) Box plots showing the normalized expression of log_2_-transformed CAV1 and miR-199a-5p in both IPF (n = 94) and control (n = 83) lungs. The box represents the 25–75% quartiles, the line in the box represents the median and whiskers represent the range. (**p<0.001). (C) Immunohistochemical analysis of CAV1 expression in lung tissue sections from IPF patients (i, ii and iii) and normal lung (iv). Three representative sections are shown (IPF n = 8 and control n = 8). (D) *In situ* hybridization (ISH) was performed to determine the localization of miR-199a-5p in lung tissue of IPF patients (i, ii and iii). ISH with scrambled probes (iv). Three representative sections are shown (n = 6).

Expression of miR-199a-5p expression was increased in lungs of IPF patients (GEO accession number GSE13316 from [13]; dataset consisting of ten IPF samples and ten control samples; two different probes for miR-199a-5p with a p-value of p = 0.005 and p = 0.006, wilcoxon rank sum test, Table S3). This result was confirmed with an independent dataset composed of 94 IPF and 83 control lungs (p<0.001) (Figure 6A) and in an additional cohort using qPCR (Figure S8). As observed in mice, IPF samples also exhibited a significant decrease in CAV1 expression (p<0.001) (Figure 6B). The linear fold ratio for CAV1 between IPF and control was 0.54 (FDR<0.05) and the linear fold ratio for miR-199a-5p for the same subjects was 1.35 (p<0.05). Finally, examination of IPF lung sections revealed a specific expression of miR-199a-5p in fibroblastic foci of the injured lung as well as a decreased CAV1 expression (Figure 6C and 6D).

### MiR-199a-5p mediates fibrogenic activation of lung fibroblasts through both CAV1-dependent and -independent pathways

**Figure 7:**
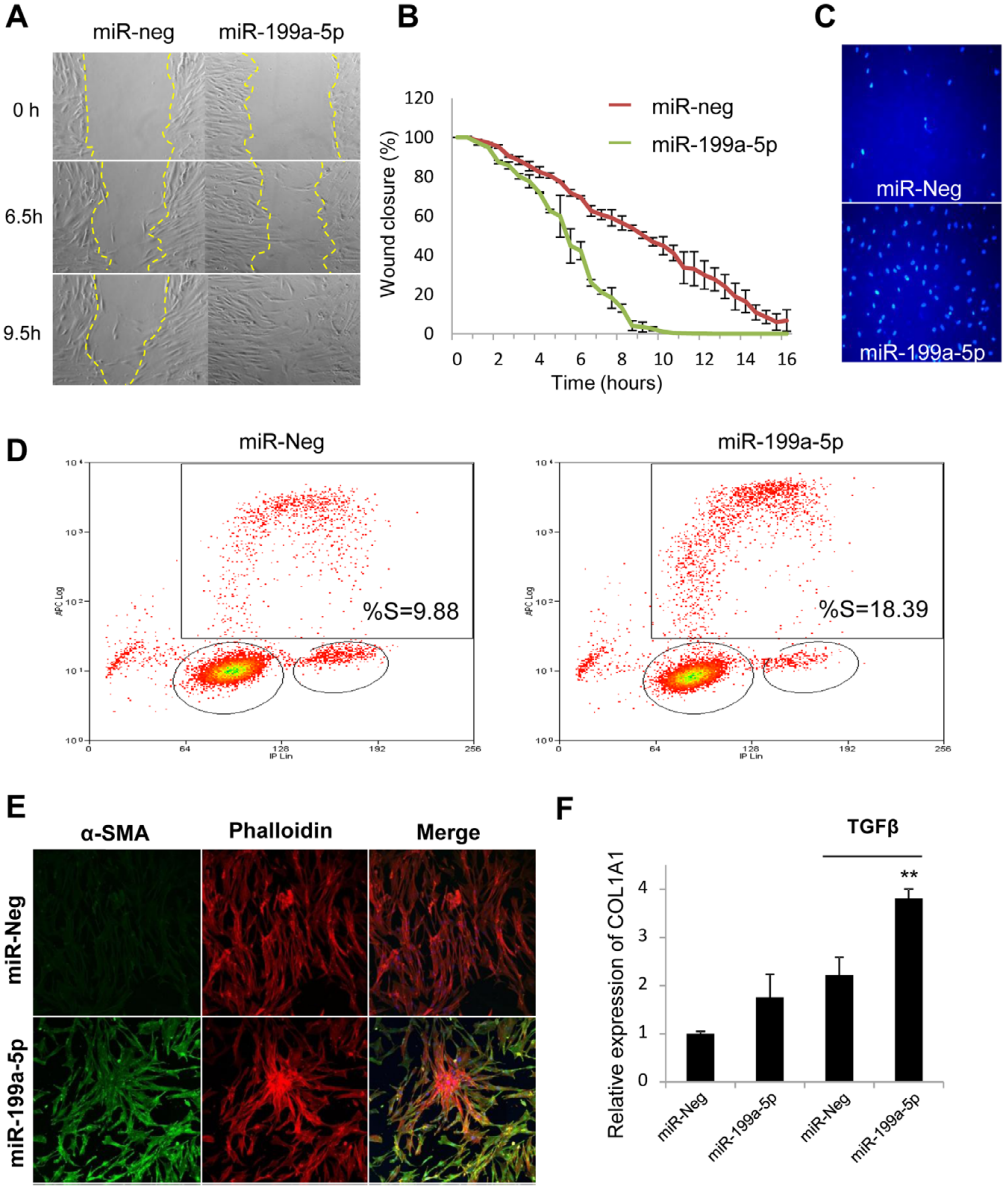
Functional impact of miR-199a-5p on lung fibroblasts. Increased expression of miR-199a-5p in lung fibroblasts results in an enhanced ability of fibroblasts to migrate, invade matrigel, proliferate and differentiate into myofibroblasts. Lung fibroblasts were transfected with 10 nM of miR-199a-5p mimic or control. (A,B) *In vitro* scratch assay was performed to assess the rate of migration of A significant increase (p<0.01) in the migration rate was observed in miR-199a-5p transfected hFL1 lung fibroblasts compared with control. Data are representative of two independent experiments. (C) Invasion assay on matrigel showing that overexpression of miR-199a-5p increases MRC5 lung fibroblast invasiveness.. Data are representative of two independent experiments. (D) Proliferation assay was performed by bivariate flow cytometric analysis of EdU/DNA-stained MRC5 cells. The x-axis represents the linear intensity obtained from propidium iodide (total DNA content), and the y-axis represents the logarithmic intensity obtained from the Alexa Fluor 647. Cells are separated into G0/G1 phase and G2/M phase according to their DNA content, and into labeled undivided and labeled divided subgroups according to the DNA content of EdU-labeled cells. The % of cells in S phase is indicated on the upper right panel. One representative experiment out of three is shown. (E) Confocal microscopy of MRC5 cells overexpressing miR-199a-5p revealed that increasing miR-199a-5p levels in lung fibroblasts promotes their differentiation into myofibroblasts. Cells were stained with an antibody against α-SMA (green) and phalloidin (red). Experiments were performed twice. (F) Relative expression of COL1A1 was assessed by Taqman PCR in MRC5 cells overexpressing miR-199a-5p and exposed or not to TGFβ. Data are expressed as mean ± SEM and derived from 2 independent experiments. *p<0.05.

Given that loss of CAV1 expression represents a critical factor involved in the fibrogenic activation of pulmonary fibroblasts [19], we assessed whether overexpression of miR-199a-5p in lung fibroblasts was sufficient to recapitulate known profibrotic effects associated with a decrease in CAV1 expression (i.e. ECM synthesis, fibroblasts proliferation, migration, invasion and differentiation into myofibroblasts) [30], [32], [33]. Transfection of miR-199a-5p precursors resulted in a significant induction of migration (Figure 7A and 7B) and invasion (Figure 7C). In addition, cell cycle analysis (percent cells in S phase) showed that proliferation rate of pulmonary fibroblasts overexpressing miR-199a-5p was significantly enhanced (Figure 7D). Finally, heterologous expression of miR-199a-5p also led to a strong increase in α smooth mucle actin (αSMA) expression (Figure 7E and Figure S9), a hallmark of myofibroblast differentiation as well as to a significant potentiation of COL1A1 induction in response to TGFβ (Figure 7F).

**Figure 8:**
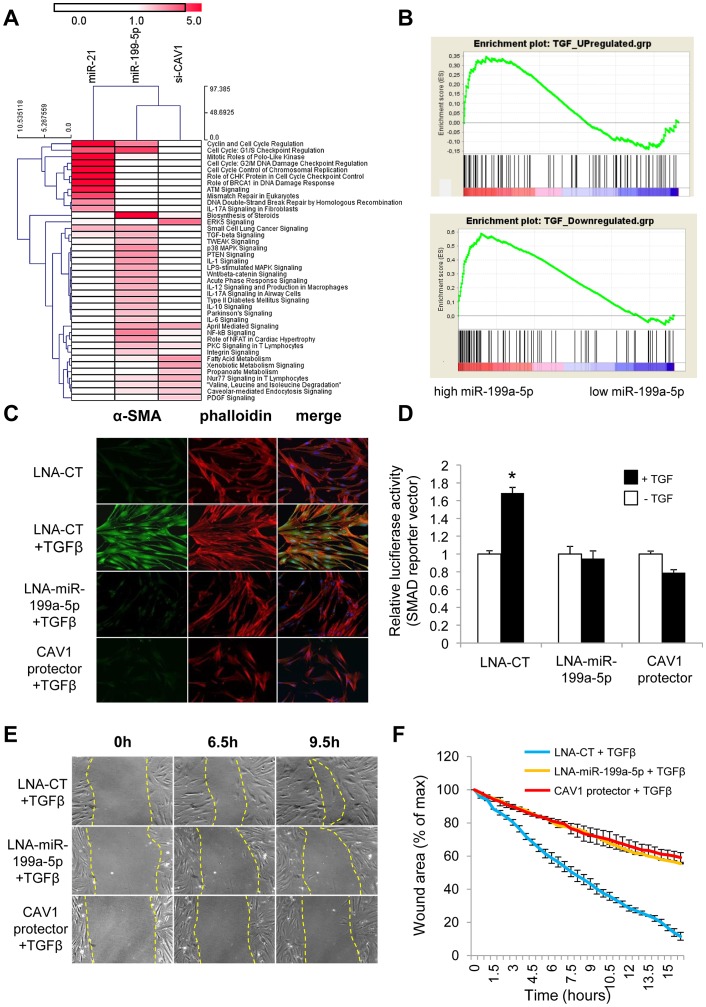
miR-199a-5p is an effector of the TGFβ pathway. (A) Heatmap of significant canonical pathways associated with miR-199a-5p, miR-21 and siCAV1 conditions identified through Ingenuity Pathway Analysis. (B) Gene Set enrichment analysis plots for an experimental TGFβ-signature showing significant enrichment for miR-199a-5p up- and down-regulated genes. Data were treated according to Gene Set Enrichment Analysis (GSEA) software, with transcripts being ordered according to miR-199a-5p versus miR-Neg conditions scaled by their standard deviation. Up- and Down-regulated genes were run separately against an experimental TGFβ signature obtained in the same cellular models (hFL1 fibroblasts treated with 10 ng/ml TGFβ during 48 h): 2 sets of 134 and 113 genes corresponding to TGFβ up- and down-regulated gene sets have been selected as described in the Materials and Method section (p<0.05). In each case, graphs show the enrichment plot, the score at the peak corresponding to the ES and the position of the TGFβ gene set in the ranked list of genes (each transcript indicated by a vertical bar. (C) MRC5 cells were transfected with LNA-miR-199a-5p, CAV1 protector or LNA-control, then incubated with or without 10 ng/ml TGFβ for 24 h and cells were stained with an antibody against α-SMA (green), phalloidin (red) and DAPI (blue). Experiments were performed twice. (D) Cells were co-transfected with SMAD-luciferase reporter plasmid, luciferase activities were analyzed 48 h after transfection. All firefly luciferase activities were normalized with renilla luciferase activity. * p<0.05. Two independent experiments. (E) and (F) *In vitro* scratch assay was performed to assess the migration rate of TGFβ-stimulated hFL1 lung fibroblasts treated with LNA-anti-miR-199a-5p, LNA-control inhibitor and CAV1 protector. A significant decrease (p<0.01) in the migration rate was observed in both LNA-anti-miR-199a-5p and CAV1 protector transfected lung fibroblasts compared with control. Data are representative of two independent experiments.

Comparison of the gene expression profiles obtained in lung fibroblasts transfected with miR-199a-5p precursors or with a siRNA specifically directed against CAV1 revealed an overlap between the 2 signatures, mainly among the down-regulated transcripts (Figure S10A, group 2): 34% of miR-199a-5p downregulated transcripts were also repressed by a siCAV1 (Figure S10B). To gain insights into the pathways modulated by miR-199a-5p, Ingenuity Pathways canonical pathways associated to miR-199a-5p were analyzed and compared to those of miR-21 and siCAV1 conditions. This analysis revealed some proximity between miR-199a-5p and siCAV1 based on the existence of shared regulated pathways (Figure 8A). Pathways that were specific to miR-199a-5p were related to inflammation, such as “IL-1 Signaling”, “Acute Phase Response Signaling” and “P38 MAPK Signaling”, i.e. all typical of fibrotic processes. Importantly, several profibrotic genes were specifically regulated by miR-199a-5p and their altered expression was confirmed *in vivo* (Figure S11 and Table S4). MiR-199a-5p thus regulates multiple signaling pathways involved in lung fibrogenesis. In particular, compared to siCAV1 transfected cells, overexpression of miR-199a-5p significantly increased *CCL2*, *TGFBRI* and *MMP3* expression and significantly decreased *CAV2* and *PLAU* expression (Figure S12). Of note, these two downregulated genes were predicted to be direct targets of miR-199a-5p by Pictar.

### MiR-199a-5p is an effector of TGFβ signaling in lung fibroblasts by regulating CAV1

We next investigated whether miR-199a-5p is associated with TGFβ signaling. For this, we experimentally defined a TGFβ signaling signature in lung fibroblasts (Dataset 2, GSE34815) and compared it to miR-199a-5p signature using gene set enrichment analysis (GSEA) [34]. This analysis revealed a significant overlap between these two signatures, as assessed by normalized enrichment scores above 1 (1.4 and 2.17 for up- and down-regulated genes respectively, with nominal p-value and FDR q-value being <0.05), suggesting therefore, that miR-199a-5p is involved in the TGFβ response of lung fibroblasts (Figure 8B). To further demonstrate the importance of miR-199a-5p in TGFβ response, silencing of miR-199a-5p was performed in lung fibroblasts using LNA-based inhibitors. In particular, we showed that LNA-mediated silencing of miR-199a-5p strongly inhibited TGFβ-induced differentiation of lung fibroblasts into myofibroblasts (Figure 8C and Figure S6), SMAD signaling (Figure 8D) and stimulation of wound repair (Figure 8E and 8F). Remarkably, similar results were obtained using CAV1 protector, demonstrating therefore that miR-199a-5p is a key effector of TGFβ response through CAV1 regulation (Figure 8C, 8D, 8E, 8F and Figure S6).

### MiR-199a-5p is dysregulated in mouse models of kidney and liver fibrosis

**Figure 9:**
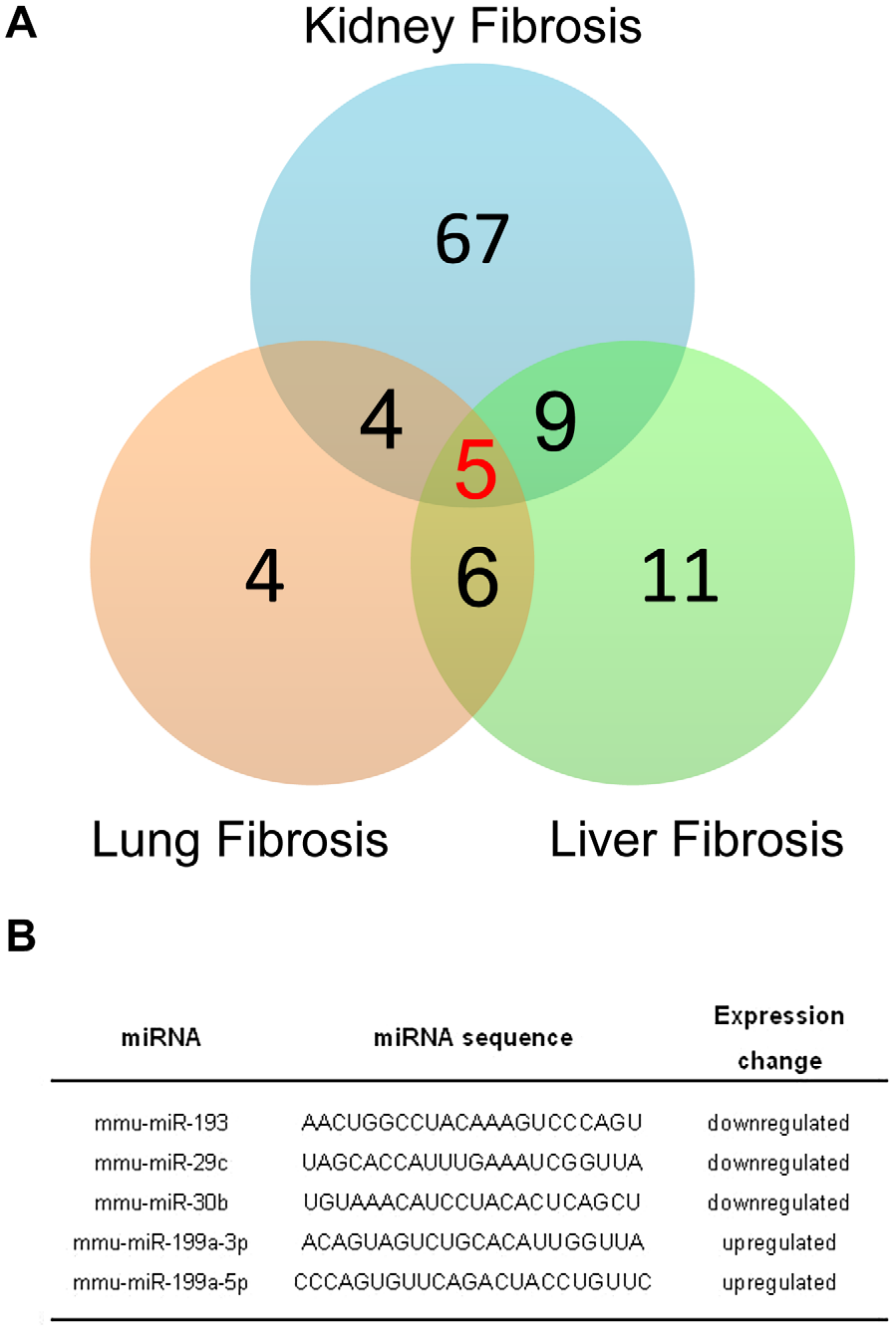
MiR-199a-5p is commonly dysregulated in three experimental mouse models of liver, lung, and kidney fibrosis. (A) Venn diagram showing the relationships of miRNA expression changes in lungs from C57BL/6 14 days after Bleomycin treatment (n = 4 mice), livers from BALB/C mice 6 weeks after CCl_4_ administration (n = 5 mice) and kidneys from C57BL/6 mice 28 days following unilateral ureteral obstruction (n = 4 mice). The numbers of miRNAs whose expression was differentially detected in each mouse model at p<0.01 are shown. Data on liver fibrosis are from (17). (B) List of the 5 miRNAs dysregulated in the three experimental models of fibrosis. ttest, p<0.01.

A growing body of evidence suggests that miRNAs contribute to the fibrotic process in various organs such as heart, kidneys, liver or lungs. For example, previous reports have shown that miR-21 has an important role in both pulmonary and heart fibrosis experimental mouse models. Thus, we investigated whether miR-199a-5p was also dysregulated in other fibrotic tissues, namely kidney fibrosis and liver fibrosis. To this end, we assessed the overlap between the miRNA expression profiles corresponding to three experimental models of fibrosis. Measurements were made using the same miRNA based platform. We identified 5 miRNAs commonly dysregulated at a p-value of less than 0.01 (Figure 9A). Among these miRNAs, 3 were downregulated (miR-193, miR-30b and miR-29c) and 2 were upregulated (miR-199a-3p and miR-199a-5p) (Figure 9B).

**Figure 10:**
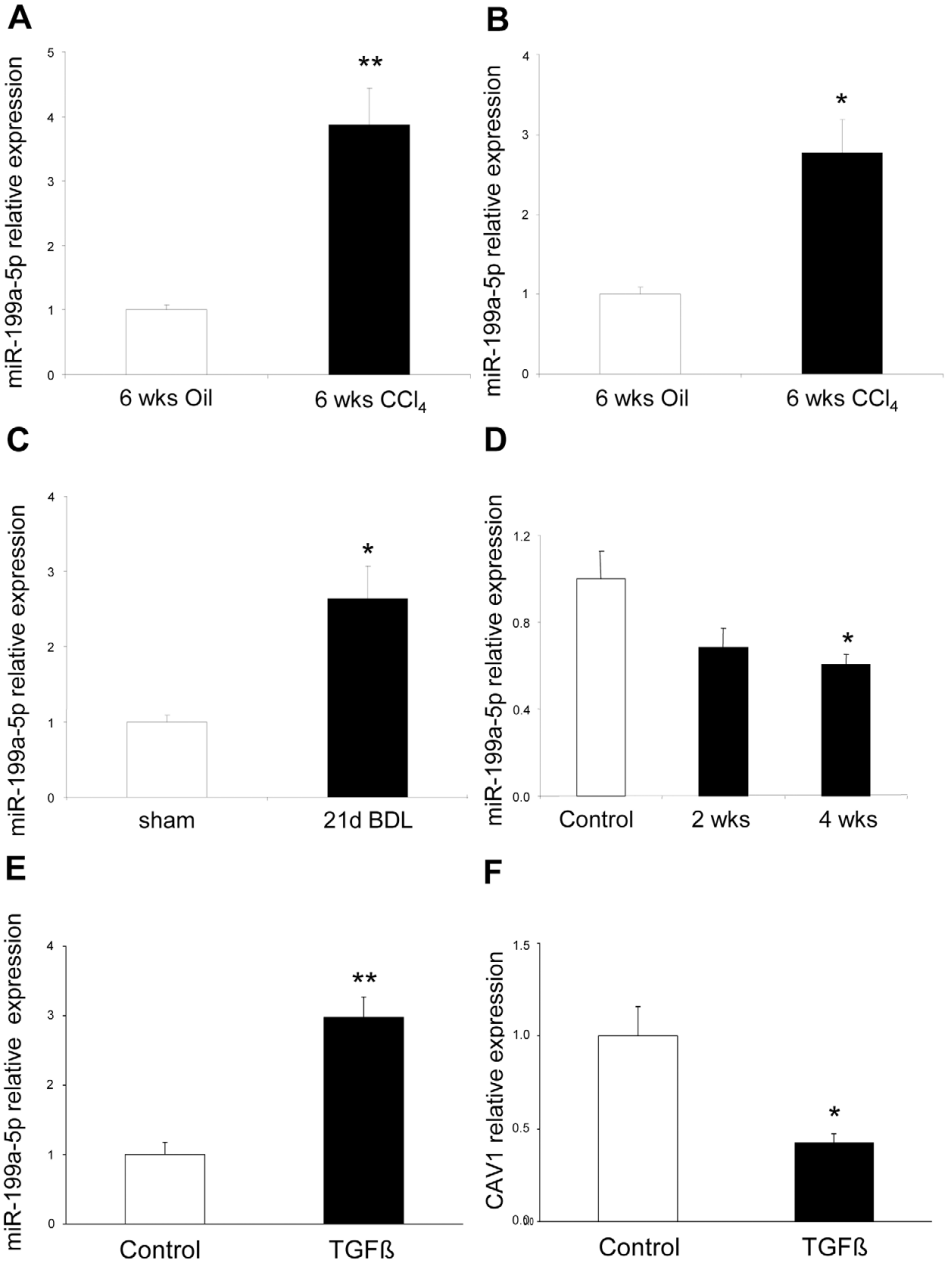
Altered expression of miR-199a-5p in CCl_4_ induced liver fibrosis mouse model. (A) Expression of miR-199a-5p in livers from 6 weeks CCl_4_-treated or oil-treated BALB/C mice was analyzed by qPCR; n = 5 per group. Data are expressed as mean ± SEM. ** p<0.01. (B) Expression of miR-199a-5p in livers from 6 weeks CCl_4_-treated or oil-treated C57BL/6 mice was analyzed by qPCR; n = 5 mice per group. Data are expressed as mean ± SEM. * p<0.05. (C) Expression of miR-199a-5p in livers from C57BL/6 mice 21 days following bile duct ligation or sham operation was analyzed by qPCR; n = 4 mice per group. Data are expressed as mean ± SEM. * p<0.05. (D) Expression of miR-199a-5p in liver of C57BL/6 mice during fibrosis regression. Liver fibrosis was induced by injection of CCl_4_ and expression of miR-199a-5p was assessed 6 weeks after CCl_4_ treatment as well as 2 and 4 weeks after the last injection. Data are expressed as mean ± SEM. * p<0.05; Expression of miR-199a-5p (E) and CAV1 (F) in primary stellate cells isolated from C57BL/6 mice and stimulated with 20 ng/ml TGFß for 48 h.

The enhanced expression of miR-199a-5p was confirmed in two independent experimental models of liver fibrosis (Figure 10A–10C) and was correlated with the severity of liver fibrosis, as BALB/C mice have a more pronounced liver fibrosis than C57BL/6 mice, following administration of CCL_4_ (Figure 10A and 10B). In addition, miR-199a-5p was significantly decreased during regression of experimental CCL_4_-induced liver fibrosis (Figure 10D). Furthermore, we showed that TGFβ exposure of stellate cells was associated with an increase of miR-199a-5p expression and a decrease of CAV1 expression level (Figure 10E and 10F). Interestingly, enhanced expression of miR-199a-5p was also observed in clinical samples from patients with liver fibrosis (Figure S13).

**Figure 11:**
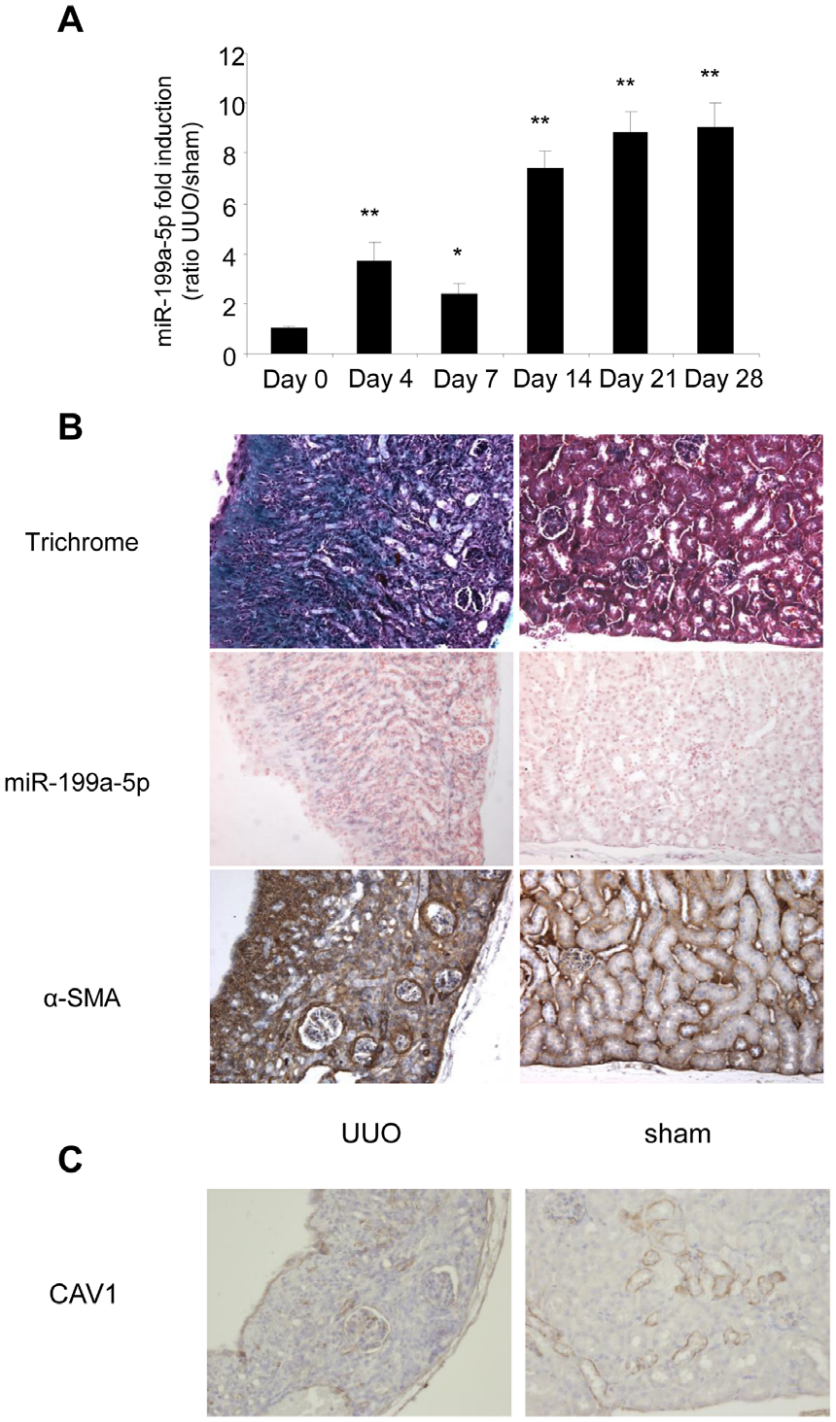
Altered expression of miR-199a-5p and CAV1 in the unilateral ureteral obstruction (UUO) mouse model of kidney fibrosis. (A) miR-199a-5p expression in kidney from C57BL/6 mice after UUO at the indicated time points. n = 5 to 7 mice in each group. Data are expressed as mean ± SEM. * p<0.05 and ** p<0.01. (B) Paraffin sections were prepared from kidneys of C57BL/6 mice harvested 28 days after UUO. *In situ* hybridization assay was performed to determine the localization of miR-199a-5p in the kidney. Results represent one out of three independent experiments. (C) Immunohistochemical analysis of CAV1 expression in kidney tissue sections from C57BL/6 mice 28 days after UUO. One representative section out of two is shown.

Similarly, data obtained from the unilateral ureteral obstruction model of kidney fibrosis showed an enhanced expression of miR-199a-5p in the injured kidney compared to sham operated mice (Figure 11A). Interestingly, as for lung fibrosis, kidney expression of miR-199a-5p was correlated with disease progression. As depicted in Figure 11B, *in situ* hybridization performed 28 days after surgery (*i.e.* when the fibrosis is established) showed no detectable signal for miR-199a-5p in normal kidney, whereas the hybridization signal was greatly enhanced throughout the injured kidney in area consistent with (myo)fibroblasts. Furthermore, immunohistochemistry of CAV1 performed on fibrotic kidney from mice 28 days after surgery showed a marked reduction of CAV1 in fibrotic area of the kidney (Figure 11C).

## Discussion

MiRNA expression profiling using high-throughput genomic approaches has provided important new insights into the pathogenesis, classification, diagnosis, stratification, and prognosis of many human diseases including tissue fibrosis [15], [35], [36]. In particular, such approaches have been previously successfully applied to IPF, revealing miR-21 and let-7d as important contributors to the lung fibrosis process [13], [14]. Our work represents however, to our knowledge, the first analysis of miRNAs involved into the differential susceptibility of two murine strains to bleomycin-induced lung fibrosis. The identification of a specific miRNA profile associated with bleomycin-sensitive animals suggests the functional importance of these dysregulated miRNAs during the pathogenic processes leading to lung fibrosis. MiR-199a-5p appeared as the most statistically significant and was well correlated to IPF progression. Thus, altered expression of miR-199a-5p is likely to represent a primary pathogenic mechanism in the development of lung fibrosis rather than a secondary effect of the long-standing disease process. Other miRNAs candidates including miR-214, clustered with miR-199a-2 on mouse chromosome 1 as well as other miRNAs that have been previously associated to fibrosis, including miR-221-222 and miR-449a [37], [38] also showed an enhanced expression in the lung fibrosis-susceptible mice. These miRNAs need to be further analyzed in IPF samples, as previous studies have shown their implication in the regulation of the stress response or the cell cycle/apoptosis balance in the epithelial or fibroblast compartment [38]–[41].

MiR-199a is an evolutionary conserved small RNA initially identified in the context of inner ear hair cells development and chondrogenesis [42]–[44] and numerous reports have now shown its implication in various tumor types [45]–[47]. In the context of tissue fibrosis, both mature forms of miR-199a (*i.e.*, miR-199a-5p and miR-199a-3p) have been associated with the progression of liver fibrosis in both humans and mice [48], [49]. While our data also showed an enhanced pulmonary expression of these two miRNAs in the bleomycin-induced mouse model, expression of miR-199a-5p was more significant in IPF samples (Table S3 and Figure S14A). Moreover, our data indicated that miR-199a-3p had distinct effects on lung fibroblasts differentiation than miR-199-5p, as assessed by their different impact on αSMA (Figure S14B and data not shown). This led us to investigate in depth miR-199a-5p profibrotic effects.

In a recent report describing the miRNA expression profile of lung fibroblasts, miR-199a-5p was found to be highly expressed [25]. Our present data establish stromal cells as the primary source of miR-199a-5p in the injured lungs and also suggests that miR-199a-5p is involved in the profibrotic effects mediated by pulmonary fibroblasts. A combination of *in silico* and experimental data, described in [25], [26], [40], identified the transcripts affected by miR-199a-5p in lung fibroblasts. Functional annotations of the miR-199a-5p experiments highlighted terms such as “Integrin Signaling” and “Caveolar-mediated Endocytosis Signaling”. Among the set of transcripts that were down-regulated after ectopic expression of miR-199a-5p, we then restricted our work to a group of 21 miR-199a-5p target genes predicted by 3 independent algorithms, showing the largest modulation factors and smallest statistical p-values. This short list included *CAV1*, a structural component of caveolae, previously associated with lung fibrosis [14], [18], [19].

**Figure 12:**
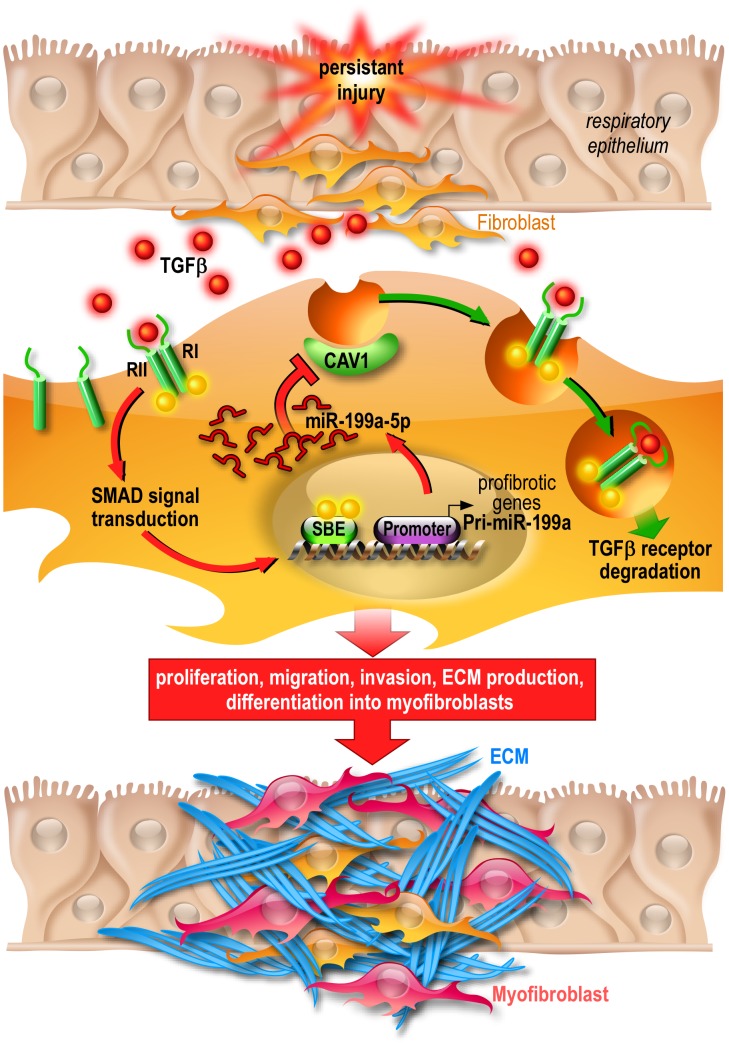
Proposed model of miR-199a-5p profibrotic function in lung fibrogenesis. Persistent injury of the respiratory epithelium causes release of profibrotic factors such as TGFβ. In lung fibroblasts, TGFβ binds to TGFβR I (RI) and II (RII) and the resultant complex can be internalized via two distinct endocytic pathways. The clathrin-coated pit pathway (red arrows) increases TGFβ signal transduction leading to the activation of lung fibroblasts and ultimately lung fibrosis whereas the CAV1 lipid raft pathway (green arrows) mediates TGFβ/TGFβR complex degradation, thus decreasing TGFβ signaling and preventing lung fibrosis [73]. Production of miR-199a-5p in response to TGFβ in lung fibroblasts results in CAV1 downregulation and subsequently, impaired TGFβ/TGFβR complex degradation. This miRNA-mediated mechanism for low CAV1 expression promotes TGFβ signaling and the pathogenic activation of lung fibroblasts. CAV1 = caveolin-1, TGFβ = Transforming Growth factor-β, TGFβR = Transforming Growth factor-β Receptor, SBE = SMAD Binding Element, ECM = Extracellular Matrix.

Caveolae refer to 50–100 nanometers small bulb-shaped invaginations of the plasma membrane. They exert major biological functions in numerous cellular processes such as membrane trafficking or cell signaling [50]. CAV1 and CAV2, the main coat proteins of caveolae, are relatively highly expressed in endothelial cells and fibroblasts of pulmonary origin [51]. Caveolae role is particularly important in the context of TGFβ signaling. Whereas TGFβ receptor endocytosis via clathrin-coated pit-dependent internalization promotes TGFβ signaling, the lipid raft-caveolar internalization pathway facilitates the degradation of TGFβ receptors, therefore decreasing TGFβ signaling [52]. Previous studies have shown that a reduced CAV1 expression in lung fibroblasts contributes to IPF pathogenesis by promoting TGFβ profibrotic effects [19]. In line with this, we provide evidence that miR-199a-5p can directly repress CAV1 in lung fibroblasts, thereby stimulating their proliferation, migration, invasion and differentiation into myofibroblasts (Figure 12). Additionally, we showed in a large cohort of IPF patients an enhanced expression of miR-199a-5p that was reproduced in three independent mouse models of fibrosis as well as a decreased expression of CAV1. Finally, in contrast to a recent report showing that miR-199a-5p, by targeting SMAD4, inhibited TGFβ-induced gastric cancer cell growth [53], we found that lung fibroblasts overexpressing miR-199a-5p have an increased SMAD4 expression (Figure S14B), suggesting thus a potential opposite function of this miRNA between epithelial and mesenchymal cells.

MiRNAs, by affecting the expression of multiple genes, can act as master regulators of complex biological processes and aberrant expression of miRNA is known to have a profound impact on various distinct biological pathways. Thus, the elucidation of the critical genes and relevant pathways/networks modulated by miRNAs is important to understand the mechanisms by which miRNAs exert their pathogenic effects. Our systematic analysis of the gene expression profiles of lung fibroblasts overexpressing miR-199a-5p led to the identification of a large number of transcripts that were significantly modulated by this miRNA. These experiments have established that miR-199a-5p is directly or indirectly involved in the regulation of genes previously associated with lung fibrosis: *CCL2*, a potent mononuclear cell chemoattractant, *PLAU* [54], a component of the fibrinolysis system, *TGFBRI*, the TGFβ receptor type I [55], *MMP3* [56] and *CAV2* [57]. Interestingly, these regulations were independent of CAV1 targeting, suggesting therefore that miR-199a-5p modulates the expression of several components of various distinct biological pathways to elicit, in lung fibroblasts, a profibrotic response.

Before this study, miR-21 was clearly established as an effector of TGFβ signaling, able to promote fibroblast proliferation and differentiation into myofibroblasts [58]. In the context of lung fibrosis, miR-21 has been described to mediate lung fibroblast activation and fibrosis [14]. MiR-199a-5p and miR-21 exert indeed similar pro-fibrotic effects on lung fibroblasts. This is further demonstrated by overexpression of miR-21 and miR-199a-5p, which induce lung fibroblast migration to a similar extent (Figure S15). Interestingly, while both miRNAs appear as TGFβ effectors, the comparison of their associated gene expression signature indicated a limited overlap (Figure 2A). Moreover, CAV1 expression is unaffected by overexpression of miR-21 in lung fibroblasts, suggesting that both miRNAs, in response to TGFβ, modulate distinct signaling pathways to produce cooperative effects involved in fibroblast activation.

The mechanisms involved in the TGFβ-dependent modulation of miR-21 and miR-199a-5p are also of particular interest. While both miR-21 and miR-199a-5p have been shown to be regulated by TGFβ, their expression may be primarily regulated through a Smad-dependent post-transcriptional mechanism promoting miRNA maturation by Drosha [59], [60]. Our data showing that both pri-miRNA-199a1 and pri-miRNA-199a2 are significantly upregulated in bleomycin-treated mice (Figure S2B) and TGFβ-stimulated fibroblasts (Figure S16) suggest that additional TGFβ-dependent transcriptional regulations occur that will need to be more fully analyzed.

Finally, our observation that miR-199a-5p is also dysregulated in two additional experimental models of tissue fibrosis (*i.e.* kidney fibrosis and liver fibrosis) establishes miR-199a-5p as a ubiquitous factor associated with tissue fibrogenesis. The recently reported association of CAV1 with kidney fibrosis [61], [62], together with the exclusive distributions of miR-199a-5p and CAV1 in the injured kidney, leads us to hypothesize that miR-199a-5p also controls CAV1 expression in kidney, thus contributing to kidney fibrosis. Further information came from the liver fibrosis model. As liver fibrosis can regress after cessation of the triggering injury, even at advanced fibrotic stages [63], the decrease of miR-199a-5p observed during resolution of liver fibrosis sets for the first time a specific miRNA as an important player for orchestrating the molecular events occurring during regression of liver fibrosis. Importantly, this implies that therapeutic strategies based on modulation of miRNAs have a potential to prevent liver fibrosis progression but also to resolve liver fibrosis.

In conclusion, the results of this study further underline the pivotal roles played by specific miRNAs in mediating changes in gene expression and cell functions occurring during pulmonary fibrosis. In particular, our results identified miR-199a-5p as a new determinant of tissue fibrosis. We thus anticipate that strategies preventing the up-regulation of miR-199a-5p may represent a new effective therapeutic option to treat fibroproliferative diseases.

## Methods

### Cell lines, reagents, and antibodies

Human normal pulmonary fibroblasts MRC-5 (CCL-171) and hFL1 (CCL-153), human lung cancer cell line A549 (CCL-185) and HEK-293 (CRL-1573) cells were purchased from the American Type Culture Collection (ATCC, Manassas, VA, USA), frozen at an early passage and each vial used for experiments was cultured for a limited number of passages (<8). For maintenance, cells were cultured in the appropriate medium (MEM for MRC-5, F12-K for hFL1 and A549, DMEM for HEK-293) containing 10% fetal calf serum (FCS), at 37°C with 5% v/v CO2. Recombinant TGFβ was purchased from Sigma-Aldrich. The following monoclonal (mAbs) and polyclonal (pAbs) Antibodies were used: rabbit anti-CAV1 pAbs (sc-894, Santa Cruz Biotechnology Inc.), rabbit anti-SMAD4 (9515) and anti- β-Actin (13E5) mAbs (Cell Signalling), mouse anti- αSMA mAbs (1A4, Dako) for immunohistochemistry and (4A8-2H3, Abnova) for Western Blot and immunocytofluorescence.

### Animal treatment

All animal care and experimental protocols were conducted according to European, national and institutional regulations. Personnel from the laboratory carried out all experimental protocols under strict guidelines to insure careful and consistent handling of the mice.

#### Mouse model of lung fibrosis

9–12 weeks old male C57BL/6 and BALB/C mice were purchased from Charles River, France. To induce fibrotic changes, mice were intratracheally instillated with bleomycin or PBS as previously described [25], [64]. Briefly, mice were anesthetized with sevoflurane inhalation (Abbott) and placed in dorsal recumbency. Transtracheal insertion of a 24-G animal feeding needle was used to instillate bleomycin (0.75 unit/ml) or vehicle (PBS), in a volume of 80 µl. Mice were sacrificed 7 and 14 days after instillation and lungs were removed for further analysis. Expression analyses were performed on 5 mice per group except for the miRNA microarray experiment where 3 mice per group were used. Histological analyses and western blot were performed on 3 C57BL/6 mice.

#### Mouse model of kidney fibrosis

9–12 weeks old male C57BL/6 mice were purchased from Charles River, France. Mice underwent anesthesia by intraperitoneal injection of pentobarbital (50 mg/kg body wt). After a standard laparotomy, the left proximal ureter was exposed and ligatured with 4-0 silk at two points. The sham operation consisted of a similar identification of the left ureter, but ligature of the ureter was not performed. Expression analyses were performed on 4 to 7 mice per group. For the histological analyses, 2 to 3 mice were used.

#### Mouse model of liver fibrosis

6–8 weeks old male C57BL/6 and BALB/C mice were purchased from Jackson laboratory (Bar Harbor). To induce liver fibrosis, mice received 0.6 ml/kg body weight of CCl_4_ (Merck) mixed with corn oil (Sigma life science) intraperitoneally as previously described [65]. Bile duct ligation (BDL) was performed by exposing the common biliary duct and double-ligaturing it, then cutting through between the ligations as described in [65]. For fibrosis regression mice were treated for 6 weeks with CCl_4_ as described above and sacrificed 2 or 4 weeks respectively after the end of treatment. For CCl_4_ induced liver fibrosis mouse model, expression analyses were performed on 5 mice whereas 4 mice were used for bile duct ligation.

### Isolation of primary stellate cells

Primary stellate cells were isolated from C57BL/6 mice at the age of 40 to 55 weeks and stimulated with 20 ng/ml of TGF-ß (Sigma Aldrich) for 48 h as previously described [65].

### Human lung tissue

Flash frozen lung tissue from 94 human subjects with IPF and 83 control subjects with no chronic lung disease were obtained from the Lung Tissue Research Consortium (LTRC). These diagnoses were made using ATS/ERS guidelines [66], [67] from review of clinical history, pathology, and radiology. All experiments were approved by the local Institutional Review Board at the University of Pittsburgh (IRB# 0411036). Clinical data were made entirely available to the investigators for review.

Paraffin lung sections from patients with IPF were obtained from Lille's Hospital. Experiments were approved by the institutional review board of Lille's Hospital.

### Histopathology

Kidneys and lungs were fixed overnight with neutral buffered formalin and then embedded in paraffin. Five-micrometer-thick sections were mounted and stained with hematoxylin and eosin as well as Masson's trichrome to assess the degree of fibrosis. Histologic sections were reviewed by an experienced pathologist.

### RNA isolation

Total RNA were extracted from lung tissue and cell samples with TRIzol solution (Invitrogen). Integrity of RNA was assessed by using an Agilent BioAnalyser 2100 (Agilent Technologies) (RIN above 7).

### Microarrays

#### MiRNA microarrays of mice lungs

The oligonucleotide sequences corresponding to 2054 mature miRNAs found in the miRNA registry (Release 8.2 [68]) are available on http://www.microarray.fr:8080/merge/index (follow the link to “microRNA”: platform referenced in GEO as GPL4718). Three biological replicates were performed for each comparison. The experimental data and microarray design have been deposited in the NCBI Gene Expression Omnibus (GEO) (http://www.ncbi.nlm.nih.gov/geo/) under serie GSE34812. The experimental design used a dye-swap approach, so that each mouse probe, printed 8 times on the microarray was measured independently 16 times for each sample. Target preparation and array hybridization were performed as previously described [24]–[26].

#### MiRNA microarray analysis of human lungs

A microRNA microarray analysis was done as previously described by us [13]. Briefly, 100 ng of total RNA was labeled and hybridized onto the Agilent microRNA Microarray Release 16.0, 8×60K. After washing, the arrays were scanned using the Agilent Microarray Scanner. The scanned images were processed by Agilent's Feature Extraction software version 9.5.3. MicroRNA microarray data analysis was done using GeneSpring v11.5 and BRB-ArrayTools v4.1 developed by Dr. Richard Simon and the BRB-ArrayTools Development Team. The data were quantile normalized. MicroRNA microarray data are publically available through the Lung Genomics Research Consortium (LGRC) website (lung-genomics.org).

#### Expression microarrays

For gene expression arrays RNA samples were labeled with Cy3 dye using the low RNA input QuickAmp kit (Agilent) as recommended by the supplier. 825 ng of labeled cRNA probe were hybridized on 8×60K high density SurePrint G3 gene expression mouse or human Agilent microarrays. Two (human *in vitro* experiments) or five (*in vivo*-derived samples) biological replicates were performed for each comparison. The experimental data have been deposited in the NCBI Gene Expression Omnibus (GEO) (http://www.ncbi.nlm.nih.gov/geo/) under SuperSerie record GSE34818 (series GSE34812 and GSE34814 for miRNA and mRNAs responses to bleomycin instillation, respectively; serie GSE34815 for miRNA/siRNA transfection experiments in human fibroblasts hFL1). For the human gene expression arrays, the data was log_2_ transformed and normalized using a cyclic loess algorithm in the R programming environment as previously described [69]. The human microarray data has been made available at the LTRC (ltrcpublic.org) and the LGRC websites as part of the LTRC protocol.

#### Statistical analysis and biological theme analysis

Normalization was performed using the Limma package available from Bioconductor (http://www.bioconductor.org). Intra slide (for 2 colours dye-swap experiments only) and inter slide normalization was performed using the Print Tip Loess and the quantile methods, respectively. Means of ratios from all comparisons were calculated and B test analysis was performed. Differentially expressed genes were selected using Benjamini-Hochberg correction of the p-value for multiple tests, based on a p-value below 0.05. Data from expression microarrays were analyzed for enrichment in biological themes (Gene Ontology molecular function and canonical pathways) and build biological networks built using Ingenuity Pathway Analysis software (http://www.ingenuity.com/) and Mediante (http://www.microarray.fr:8080/merge/index) [70], an information system containing diverse information about probes and data sets. Gene Set Enrichment Analysis (GSEA) was used to determine whether an *a-priori* defined set of genes can characterize differences between two biological states [34], [71]. Hierarchical clusterings were done with the MultiExperiment Viewer (MeV) program version 4.3, using a Manhattan distance metric and average linkage.

#### MiRNA targets analysis

MiRonTop [29] is an online java web tool (available at http://www.microarray.fr:8080/miRonTop/index) that integrates DNA microarrays data to identify the potential implication of miRNAs on a specific biological system. Briefly, MiRonTop ranks the transcripts into 2 categories (‘Upregulated’ and ‘Downregulated’), according to thresholds for expression level and for differential expression. It then calculates the number of predicted targets for each miRNA, according to the prediction software selected (Targetscan, MiRBase, PicTar, exact seed search: 2–7 or 1–8 first nucleotides of the miRNA, TarBase v1), in each set of genes. Enrichment in miRNA targets in each category is then tested using the hypergeometric function. The absence of siRNA off-target effect was checked in si-CAV1 transcriptome experiments using the Sylamer Tool [72].

### Transfection and luciferase assays

#### Pre-miRNAs, LNA-based miRNA inhibitors, target site blocker, and siRNAs transfection in lung fibroblasts

Pre-miR-199a-5p, pre-miR-21 and control miRNA (miR-Neg # 1) were purchased from Ambion. For miR-199a-5p knockdown and target site blocker experiments, anti-miR-199a-5p LNA, negative control anti-miR-159s LNA (miRCURY LNA Knockdown probes) and CAV1 protector were ordered from Exiqon. Si-RNA directed against CAV1 and control siRNA (Silencer Select validated siRNAs) were from Applied Biosystems. MRC5/hFL1 cells were grown in 10% FCS in DMEM and transfected at 30 to 40% confluency in 6- 12- or 96 well plates using Lipofectamin RNAi MAX™ (Invitrogen) with pre-miRNA, siRNAs LNA inhibitors and CAV1 protector at a final concentration of 10 nM unless indicated.

#### Pre-miRNAs and psiCHECK-2 plasmid constructs co-transfection

Molecular constructs were made in psiCHECK-2 (Promega) by cloning behind the Renilla luciferase in the XhoI and NotI restrictions sites, annealed oligonucleotides derived from the CAV1 3′ UTR described below. Mutated nucleotides located in the miR-199a-5p-binding site are underlined. HEK293 cells were plated into 96-well and cotransfected using lipofectamin 2000 (Invitrogen) with 0.2 µg of psiCHECK-2 plasmid construct and pre-miR-199a-5p or control miRNA at different concentrations. 48 hours after transfection, Firefly and Renilla Luciferase activities were measured using the Dual-Glo Luciferase assay (Promega).

hsa-CAV1: WT (sense):

TCGAGGACACTTTAATTACCAACCTGTTACCTACTTTGACTTTTTGCATTTAAAACAGACACTGGCATGGATATAGTTTTACTTTTAAACTGTGTACGC

hsa-CAV1: WT (reverse):

GGCCGCGTACACAGTTTAAAAGTAAAACTATATCCATGCCAGTGTCTGTTTTAAATGCAAAAAGTCAAAGTAGGTAACAGGTTGGTAATTAAAGTGTCC

hsa-CAV1: MUT (sense):

TCGAGGACACTTTAATTACCAACCTGTTACCTACTTTGACTTTTTGCATTTAAAACAGAGAGTCGCATGGATATAGTTTTACTTTTAAACTGTGTACGC

hsa-CAV1: MUT (reverse):

GGCCGCGTACACAGTTTAAAAGTAAAACTATATCCATGCGACTCTCTGTTTTAAATGCAAAAAGTCAAAGTAGGTAACAGGTTGGTAATTAAAGTGTCC

#### Smad reporter assay

MRC5 cells were seeded in 96 well plate and cotransfected 24 h later at 40% confluency using RNAi MAX lipofectamine reagent with 100 ng SMAD reporter vector (Cignal Smad reporter,- QIAGEN) and 10 nM LNA-control, LNA-199a-5p or CAV1 protector. Twenty four hours after transfection, cells were serum starved 3 h before adding 10 ng/ml TGFβ. Cells were lyzed and Glo luciferase assay (Promega) was performed 24 h following TGFβ exposure.

### Quantitative RT–PCR

#### Mature miRNA expression

MiR-199a-5p expression was evaluated using TaqMan MicroRNA Assay (Applied Biosystems) as specified in their protocol. Real-time PCR was performed using Universal Master Mix (Applied Biosystems) and ABI 7900HT real-time PCR machine. Expression levels of mature microRNAs were evaluated using comparative CT method (2^−deltaCT^).

#### Pri-miRNA expression

Pri-miR-199a-1 and pri-miR-199a-2 expression were evaluated using TaqMan pri-microRNA Assay (Applied Biosystems) following manufacturer's recommandations. Real-time PCR was performed using TaqMan gene expression Master Mix (Applied Biosystems) and ABI 7900HT real-time PCR machine. Expression levels of pri-miRNAs were evaluated using comparative CT method (2^−deltaCT^).

#### Gene expression

Expression levels of both human and mouse CAV1, human COL1A1 were analyzed using TaqMan MicroRNA Assay (Applied Biosystems) according to the manufacturer's instructions. Real-time PCR was performed using TaqMan Gene Expression Master Mix (Applied Biosystems) and ABI 7900HT real-time PCR machine. Expression levels were evaluated using comparative CT method (2^−deltaCT^). For normalization, transcript levels of RNU44 (human samples) and sno202 (mouse samples) were used as endogenous control for miRNA real time PCR. Transcript levels of PPIA (human and mouse samples) were used as endogenous control for gene/pri-miRNA expression.

### Protein extraction and immunoblotting

Cells or tissues were lysed in lysis buffer (M-PER protein extraction reagent for cells, T-PER protein extraction reagent for tissues) and protease inhibitors cocktail (Pierce). The lysates were quantified for protein concentrations using the Bradford assay (Biorad). Proteins (10 µg per sample) were separated by SDS-polyacrylamide gel and transferred onto nitrocellulose membranes (GE Healthcare). The membranes were blocked with 5% fat free milk in Tris-buffered saline (TBS) containing 0.1% Tween-20 (TBS-T) and subsequently incubated with CAV1, α-SMA or β-actin primary antibodies overnight at 4°C. After washing with TBS-T for 30 minutes at room temperature, the membrane was further incubated with horseradish peroxidase–conjugated secondary antibodies for 1.5 hours, followed by 30 minutes of washing with TBS-T. Protein bands were visualized with Amersham ECL substrates (GE Healthcare).

### Immunohistochemistry

Five-µm paraffin-embedded sections were sequentially incubated in xylene (5 minutes twice), 100% ethyl alcohol (5 minutes twice), 95% ethyl alcohol (5 minutes twice), and 80% ethyl alcohol (5 minutes). After washing with water, the sections were antigen-retrieved using citrate buffer (pH 6.0; DAKO) in a steamer for 20 minutes and cooled to ambient temperature. Sections were then washed with TBS-T and quenched with 3% hydrogen peroxide in TBS for 10 minutes, blocked for avidin/biotin activity, blocked with serum-free blocking reagent, and incubated with primary antibody as follows: for CAV1 staining, sections were incubated with antibody for 1 hour at ambient temperature; for alpha-SMA staining, sections were incubated with antibody overnight at 4°C. Immunohistochemical staining was developed using the DAB substrate system (DAKO).

### *In situ* hybridization

*In situ* hybridization of miR-199a-5p was performed using double DIG-labeled LNA probes (Exiqon, Woburn, MA). Paraffin-embedded mouse tissues were dewaxed in xylene and rehydrated in descending grades of alcohol. The slides were then washed in PBS (pH 7.5) and permeabilized by incubating for 15 min in proteinase K (Ambion) for 15 min at 37°C. The slides were again washed in PBS, and prehybridized in hybridization buffer (50% formamide, 5× SSC, 0.1% Tween-20, 9.2 mM citric acid, 50 µg/ml heparin, and 500 µg/ml yeast RNA, pH 6) in a humidified chamber. The double DIG-labeled LNA probes were then added to the sections at a 80 nM concentration and incubated 2 hours at 50°C in a humidified chamber. The slides were rinsed in 5× SSC, 1× SSC and 0.2× SSC solutions at the same hybridization temperature. This step was followed by blocking with 2% sheep serum, 2 mg/ml BSA in PBS+0.1% Tween-20 (PBST) and incubation with anti-DIG-AP Fab fragments antibody (1∶800) (Roche Applied Sciences) for 2 hours at room temperature. After washing in PBST, the color reaction was carried out by incubation in 5-bromo-4-chloro-3-indolyl phosphate (BCIP)/nitro blue tetrazolium (NBT) color solution (Roche Applied Sciences) with 1 mM levamisole overnight at room temperature. The color reaction was stopped after observation of sufficient development of blue precipitate by washing with PBST. The slides were then counterstained with Fastred (Sigma Aldrich), mounted and coverslipped.

### Immunofluorescence analysis

MRC5 cells were grown on a Round Glass Coverslips Ø 16 mm (thermo scientific) placed inside a 12 Multiwell Plate. Coverslips slides were washed in phosphate-buffered saline and fixed in 4% paraformaldehyde for 15 min, cells were then permeated using 0.1% Triton X-102 (Agilent Technologies) for 10 min. and blocked with PBS solution containing BSA (3%) for 30 min. Incubation with primary antibodies was performed in a bloking solution BSA (1%) at 37°C for 1 h at the following dilutions; α-SMA (1∶1000), CAV1 (1∶50),. After three washes with PBS, cells were incubated with secondary Alexa Fluor 488 goat anti-Mouse IgG (Invitrogen) (1∶500), Alexa Fluor 647 goat anti-rabbit IgG (Invitrogen) (1∶500) and Alexa Fluor 647 Phalloidin (A22287 - Life technologies) (1Unit/slide). Fourty five min later, Coverslips slides were fixed on microscope slides using ProLong Gold Antifade Reagent with DAPI (Invitrogen). Fluorescence was viewed with an FV10i Olympus confocal scanning microscope.

### Cell proliferation assay

MRC5 cells (150,000/well) were seeded in duplicate in DMEM supplemented with 10% FBS on 60-mm cell culture dishes. Cells were serum starved the next day and transfected with pre-miR-199a-5p at 10 nM. Cell proliferation was assessed 48 h after transfection by flow cytometry using the click-iT EdU cell proliferation assay (Invitrogen) according to the manufacturer's instructions.

### *In vitro* wound healing assay

hFL1 cells were seeded on Type-I collagen coated 12-well plates and transfected as described above. Forty eight hours after transfection, confluent cells were (FCS) starved 3 h before adding 10 ng/ml TGFβ and wounded using a pipet tip. The *in vitro* wound-healing process was then recorded by videomicroscopy for 24 h from then scratching on an Axiovert 200 M inverted microscope (Carl Zeiss) equipped with 37°C and 5% CO2 regulated insert (Pecon GmbH). Brightfied images were taken each 30 min through a 10× phase contrast objective with a CoolSNAPHQ CCD Camera managed by Metamorph Software (Roper Scientific). The motility of the cells was assessed by evaluating the repaired area percentage using ImageJ sotware.

### Invasion assay

Invasion of MRC5 fibroblast overexpressing miR-199a-5p was assessed using commercially available 24-well BioCoat Matrigel Invasion Chamber (BD Biosciences). In brief, pulmonary fibroblasts were transfected either with pre-miR-199a-5p or negative control as described above. Twenty four hours after transfection, cells were harvested with trypsin-EDTA, centrifuged, and resuspended in DMEM medium. Cell suspensions (1×10^5^ cells/well) were added to the upper chamber. Bottom wells of the chamber were filled with DMEM medium containing 10% FBS as chemoattractant, whereas the upper chamber was filled with DMEM only. After incubation for 48 h at 37°C, the non-invading cells on the top of the membrane were removed with a cotton swab. Membrane containing invading-cells were fixed with methanol, washed three times with PBS and mounted with DAPI hard set (Vector Laboratories) onto glass slides for fluorescent microscopy.

### Statistical analysis

Results are given as mean±S.E.M. Statistical analyses were performed by using Student's t-test as provided by Microsoft Excel.

## Supporting Information

Figure S1 Study schema.
(TIF)

Figure S2 miR-199a-5p and pri-miR-199a expression in C57BL/6 mice 14 days following bleomycin exposure. (A) Real-time PCR was performed to confirm the enhanced expression of miR-199a-5p in lungs of C57BL/6 and BALB/C mice 14 days following bleomycin exposure on an independent set of mice (n = 5 mice in each group). Data are expressed as mean ± SEM. *p<0.05. (B) Pri-miR-199a-1 and pri-miR-199a-2 gene expression in lungs from C57BL/6 mice 14 days after bleomycin instillation. n = 5 mice in each group, data are expressed as mean ± SEM. *p<0.05 and **p<0.01.
(TIF)

Figure S3 miR-21 expression during bleomycin induced lung fibrosis. (A) Normalized fluorescence expression values of miR-21 in lungs from Balb/c and C57BL/6 mice in response to bleomycin at the indicated time points from microarrays experiments (n = 3). Data are expressed as mean ± SEM. **p<0.01 (B) Real-time PCR was performed to confirm the enhanced expression of miR-21 in lungs of C57BL/6 and BALB/c mice 14 days following bleomycin exposure. n = 5 mice in each group, data are expressed as mean ± SEM. ** p<0.01. (C) Paraffin sections were prepared from C57BL/6 mice harvested 14 days following bleomycin intra-tracheal instillation. In situ hybridization was performed to show the localization of miR-21 in fibrotic area of the lungs (i–iv). Results represent one out of three independently performed experiments.
(TIF)

Figure S4 CAV1 is a direct target of miR-199a-5p. Co-transfection of pre-miR-199a-5p or pre-miR-Neg and human CAV1 3′UTR-derived psiCHECK-2 construct in A549 cells show a significant decrease in normalized luciferase activity 48 h post-transfection. * p<0.05.
(TIF)

Figure S5 Decreased CAV1 expression after transfection of MRC5 lung fibroblasts with pre-miR-199a-5p. (A) MRC5 Lung fibroblasts that were transfected with 10 nM of pre-miR-199a-5p for 48 h show a significant decrease in CAV1 expression as determined by real-time PCR. Data are expressed as mean ± SEM. **p<0.01. (B) Western blot analysis showing the downregulated expression of CAV1 protein after transfection of MRC-5 lung fibroblasts with pre-miR-199a-5p. Data are representative of two independent experiments.
(TIF)

Figure S6 MiR-199a-5p mediates TGFβ dependent differentiation of lung fibroblast into myofibroblasts through CAV1 regulation. Normal human pulmonary fibroblasts MRC5 were transfected with a control LNA inhibitor (LNA-CT), a LNA-miR-199a-5p inhibitor or a target site blocker directed against CAV1 3-UTR (CAV1 protector) (n = 2). Protein samples were harvested at 48 h post-transfection and analyzed by western Blot for CAV1 and αSMA.
(TIF)

Figure S7 Pulmonary expression of CAV1 in BALB/c mice 14 days after bleomycin injection. Real-time PCR was performed to assess the expression of CAV1 in lungs of BALB/c mice 14 days following bleomycin exposure. n = 5 mice in each group, data are expressed as mean ± SEM. n.s. = non significant.
(TIF)

Figure S8 MiR-199a-5p expression determined by qPCR using FFPE lung samples. Box plot showing the increased expression of miR-199a-5p in IPF samples (n = 10) compared to control (n = 10). Significance was evaluated using the Kruskal–Wallis rank-sum test. The box represents the 25–75% quartiles, the line in the box represents the median and whiskers represent the range.
(TIF)

Figure S9 Expression of *ACTA2* and *PPARG* following overexpression of miR-199a-5p in lung fibroblasts. Microarray analysis of lung fibroblasts transfected with 10 nM of miR-199a-5p mimic or miR-Neg reveals a significant increase of ACTA2 expression (*p<0.05), a hallmark of myofibroblast differentiation, as well as a significant decrease of *PPARG* expression (**p<0.01), a known inhibitor of myofibroblast differentiation. Data are expressed as mean of normalized fluorescence values ± SEM.
(TIF)

Figure S10 Comparison of transcriptomic changes induced by miR-199a-5p and a siRNA directed against CAV1. Normal human pulmonary fibroblasts hFL1 were transfected with pre-miR-Neg, pre-miR-199a-5p as well as siCAV1 or a control siRNA (n = 2). RNA samples were harvested at 48 h post-transfection and expression profiles were determined with pan genomic arrays. (A) Heatmap comparing the normalized log2 of the ratios between pre-miR-199a-5p versus pre-miR-Neg or siCAV1 versus siNeg signals. (B) Venn diagram comparing the set of down-regulated transcripts following miR-199a-5p and siCAV1. Cut-offs for selection are equal to 7.0 for the log_2_ (signal), 0.7 for the log_2_ (ratio), and 0.05 for the adjusted p-value.
(TIF)

Figure S11 Comparison of gene expression changes between miR-199a-5p-regulated genes in hFL1 human lungs fibroblasts and lungs from C57BL/6 mice 14 days after bleomycin injection. (A) Venn diagram showing the relationships of gene expression changes between miR-199a-5p transfected lung fibroblasts (two independent experiments) and lungs from C57BL/6 14 days after Bleomycin treatment (n = 5 mice). The numbers of genes whose expression was differentially detected in each condition at p<0.05 are shown. Microarray analysis shows a significant reduction of *CAV2* (B), *TGFBRI* (C), *CCL2* (D), *ACTA2* (E) and *MMP3* (F) expression in C57BL/6 mice treated with bleomycin for 14 days (n = 5) compared with control mice (n = 5). Data are expressed as mean ± SEM. ** p<0.01.
(TIF)

Figure S12 Profibrotic genes significantly modulated in lung fibroblasts by miR-199a-5p independently of CAV1 regulation. Lung fibroblasts were transfected by miR-199a-5p mimic, si-CAV1 or negative controls. Microarray analysis shows the expression of known profibrotic genes: *TGFBRI* (A), *MMP3* (B), *CAV2* (C), *PLAU* (D) and *CCL2* (E) 48 h after transfection. Data are expressed as mean ± SEM. *p<0.05, **p<0.01.
(TIF)

Figure S13 Enhanced expression of miR-199a-5p in clinical samples from patients with liver fibrosis. *In situ* hybridization assay was performed to determine the localization of miR-199a-5p in normal and fibrotic human livers. Results represent one out of three independent experiments.
(TIF)

Figure S14 Expression of miR-199a-3p in lung fibrosis and impact of its overexpression on pulmonary fibroblast differentiation. (A) Increased expression of miR-199a-3p and miR-199a-5p in lung samples from IPF patients (n = 10) compared to control lung (n = 10). The mean normalized fluorescence intensity for the agilent probe is displayed; (B) Western blot analysis showing the effect of miR-199-5p or miR-199a-3p overexpression in hFL1 lung fibroblasts on SMAD4 and αSMA expression. One representative experiment out of two is shown.
(TIF)

Figure S15 Transfection of human lung fibroblasts with pre-miR-199a-5p or pre-miR-21 increases cell motility. Sratch wound was induced in confluent cell monolayers plated on plastic and wound closure was measured using image J software (2 independent experiments).
(TIF)

Figure S16 Effect of TGFβ on mature and pri-miRNAs forms of miR-199a in human lung fibroblasts. HFL1 human fibroblasts were treated or not with 10 nM TGFβ for 48 hours. Real Time TaqMan PCR showing the levels of (A) mature miR-199a-5p and miR-199a-3p; (B) pri-miR-199a-1 and pri-miR-199a-2. n = 2. Data are expressed as mean ± SEM *p<0.05.
(TIF)

Table S1 List of themes corresponding to “canonical pathways” annotations identified by Ingenuity Pathway Analysis in response to overexpression of miR-199a-5p or miR-21 in human pulmonary fibroblasts hFL1. Normal human pulmonary fibroblasts hFL1 were transfected with pre-miR-Neg, pre-miR-199a-5p or pre-miR-21 (n = 2). RNA samples were harvested at 48 h post-transfection and expression profiles were determined with whole genome microarrays. The probability to obtain the number of genes in a certain pathway in the list of differentially expressed genes between either miR-199a-5p or miR-21 versus miR-Neg was compared with the representation of the same pathway among all the genes on the microarray; −log10 of the Fisher's exact probability is indicated. Significant pathways are shown in progressively brighter shades of orange according to their significance. ns = non significant.
(DOCX)

Table S2 List of themes corresponding to “canonical pathways” annotations identified by Ingenuity Pathway Analysis in lungs from C57BL/6 mice treated with bleomycin. RNAs extracted from C57BL/6 mice lungs 14 days after instillation with bleomycin or PBS were analyzed with whole genome microarrays (n = 5).The probability to obtain the number of genes in a certain pathway in the list of differentially expressed genes between bleomycin and PBS conditions was compared with the representation of the same pathway among all the genes on the microarray; −log10 of the Fisher's exact probability is indicated.
(DOCX)

Table S3 Pulmonary expression of miR-199a-5p in 10 IPF and 10 controls (dataset GEO accession number GSE13316).
(DOCX)

Table S4 List of the 133 genes modulated by miR-199a-5p in lung fibroblasts that are also dysregulated in lungs from C57BL/6 mice 14 days after bleomycin treatment.
(DOCX)
