# Supplementary figures and images for: miR-199a-5p Is Upregulated during Fibrogenic Response to Tissue Injury and Mediates TGFbeta-Induced Lung Fibroblast Activation by Targeting Caveolin-1
Source: PLoS Genet. 2013 Feb 14;9(2):e1003291. doi: 10.1371/journal.pgen.1003291 (PMC3573122; doi:10.1371/journal.pgen.1003291)

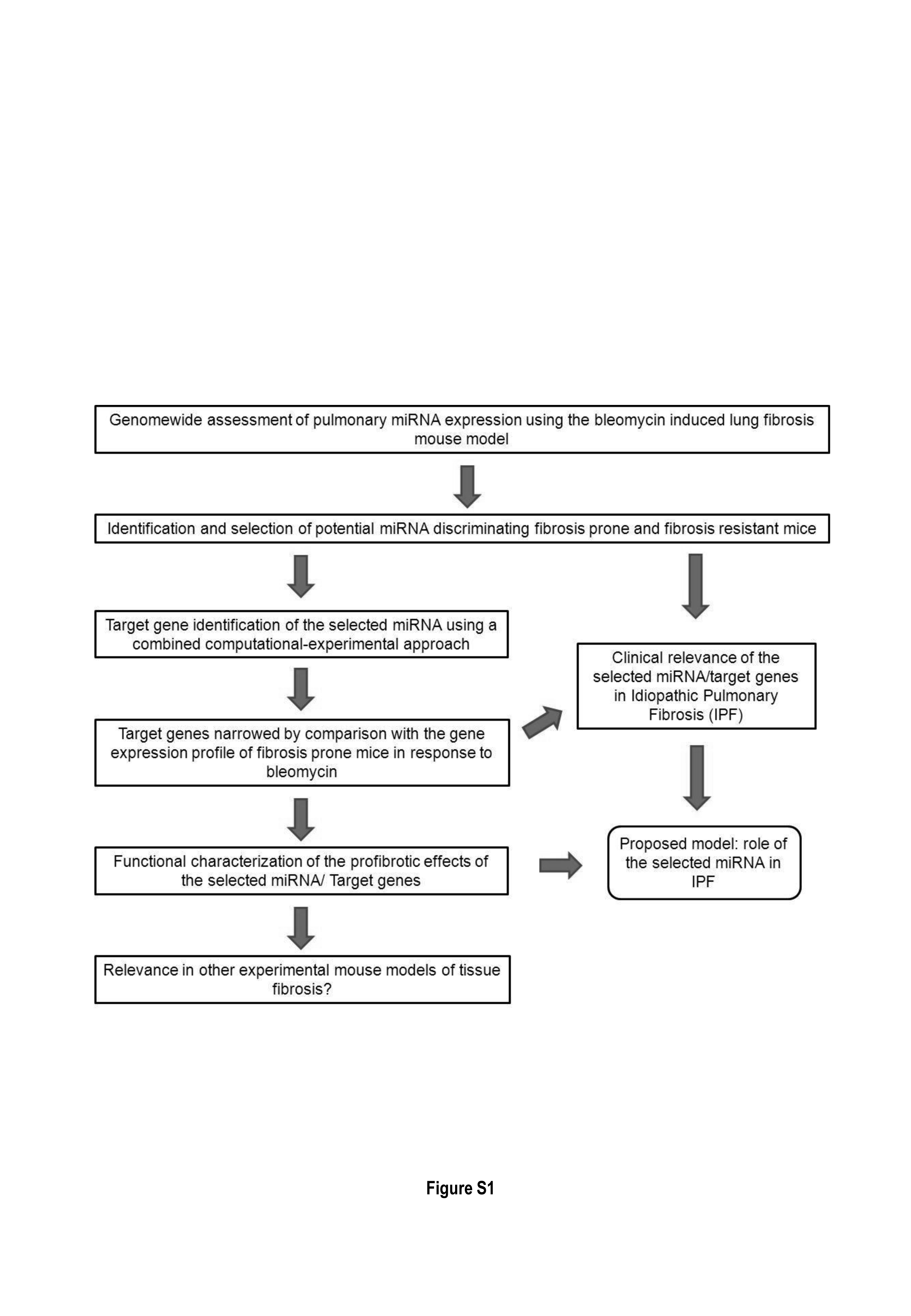

Supplement: Figure S1 — Study schema. (TIF) [file pgen.1003291.s001.tif]

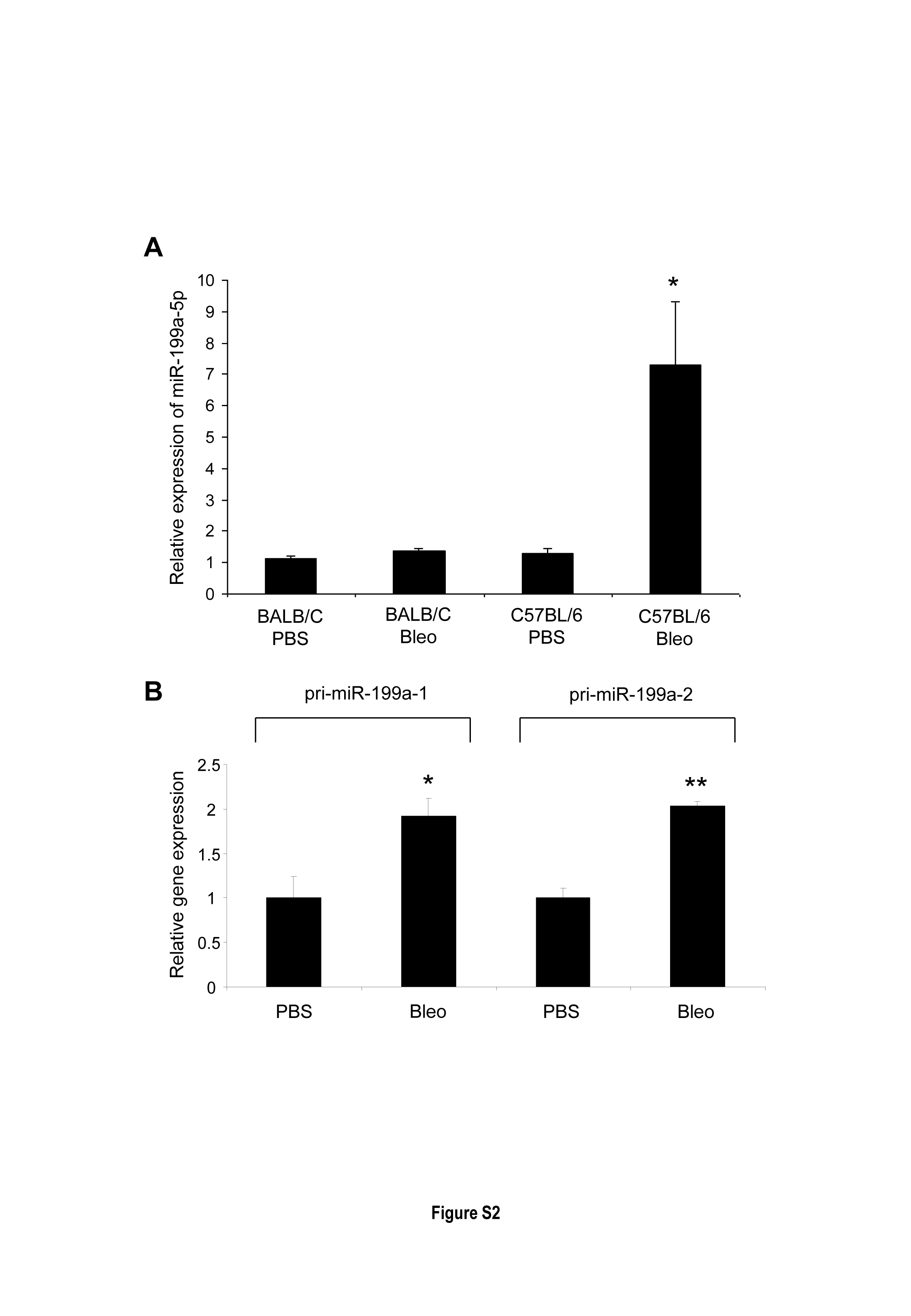

Supplement: Figure S2 — miR-199a-5p and pri-miR-199a expression in C57BL/6 mice 14 days following bleomycin exposure. (A) Real-time PCR was performed to confirm the enhanced expression of miR-199a-5p in lungs of C57BL/6 and BALB/C mice 14 days following bleomycin exposure on an independent set of mice (n = 5 mice in each group). Data are expressed as mean ± SEM. *p<0.05. (B) Pri-miR-199a-1 and pri-miR-199a-2 gene expression in lungs from C57BL/6 mice 14 days after bleomycin instillation. n = 5 mice in each group, data are expressed as mean ± SEM. *p<0.05 and **p<0.01. (TIF) [file pgen.1003291.s002.tif]

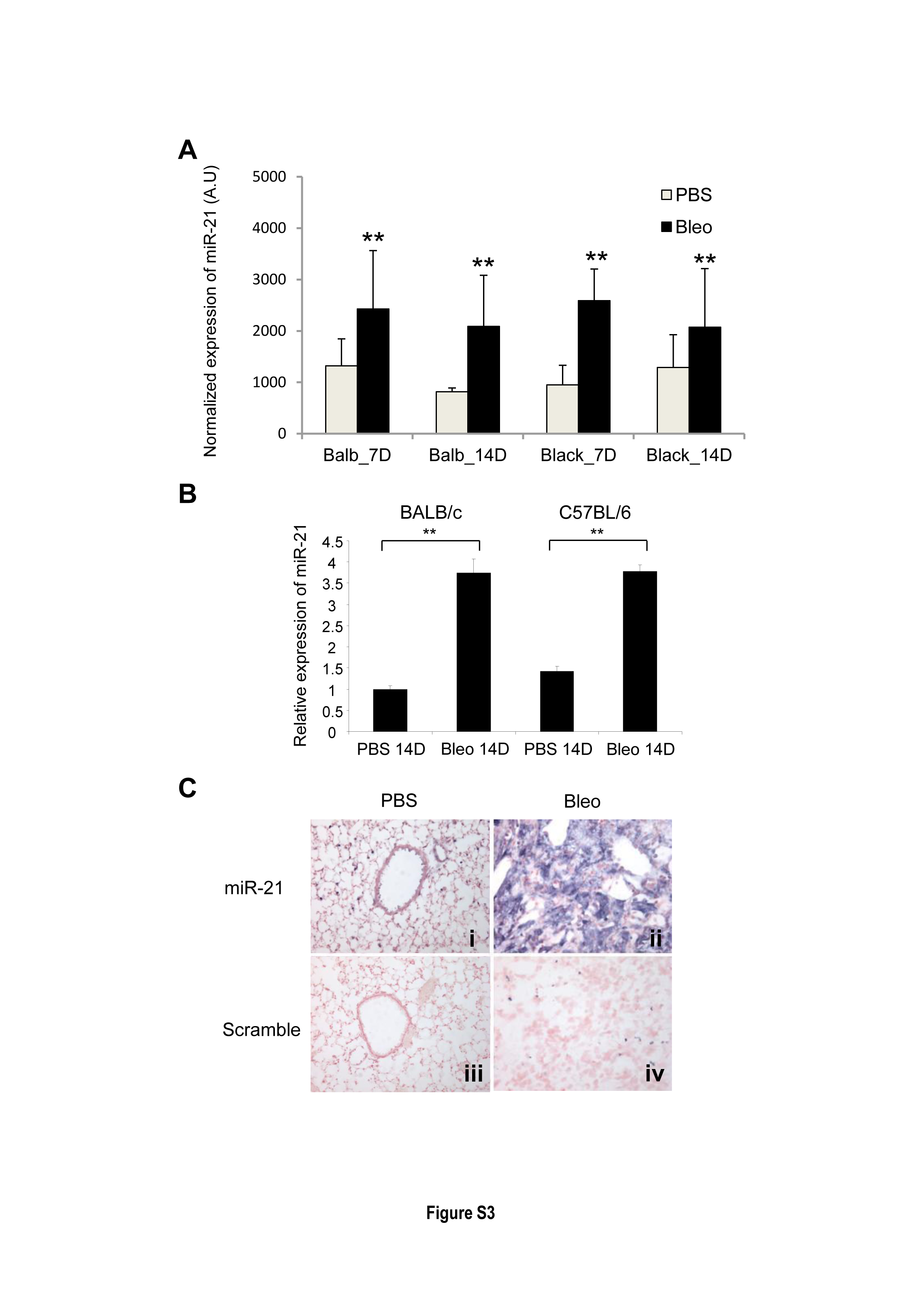

Supplement: Figure S3 — miR-21 expression during bleomycin induced lung fibrosis. (A) Normalized fluorescence expression values of miR-21 in lungs from Balb/c and C57BL/6 mice in response to bleomycin at the indicated time points from microarrays experiments (n = 3). Data are expressed as mean ± SEM. **p<0.01 (B) Real-time PCR was performed to confirm the enhanced expression of miR-21 in lungs of C57BL/6 and BALB/c mice 14 days following bleomycin exposure. n = 5 mice in each group, data are expressed as mean ± SEM. ** p<0.01. (C) Paraffin sections were prepared from C57BL/6 mice harvested 14 days following bleomycin intra-tracheal instillation. In situ hybridization was performed to show the localization of miR-21 in fibrotic area of the lungs (i–iv). Results represent one out of three independently performed experiments. (TIF) [file pgen.1003291.s003.tif]

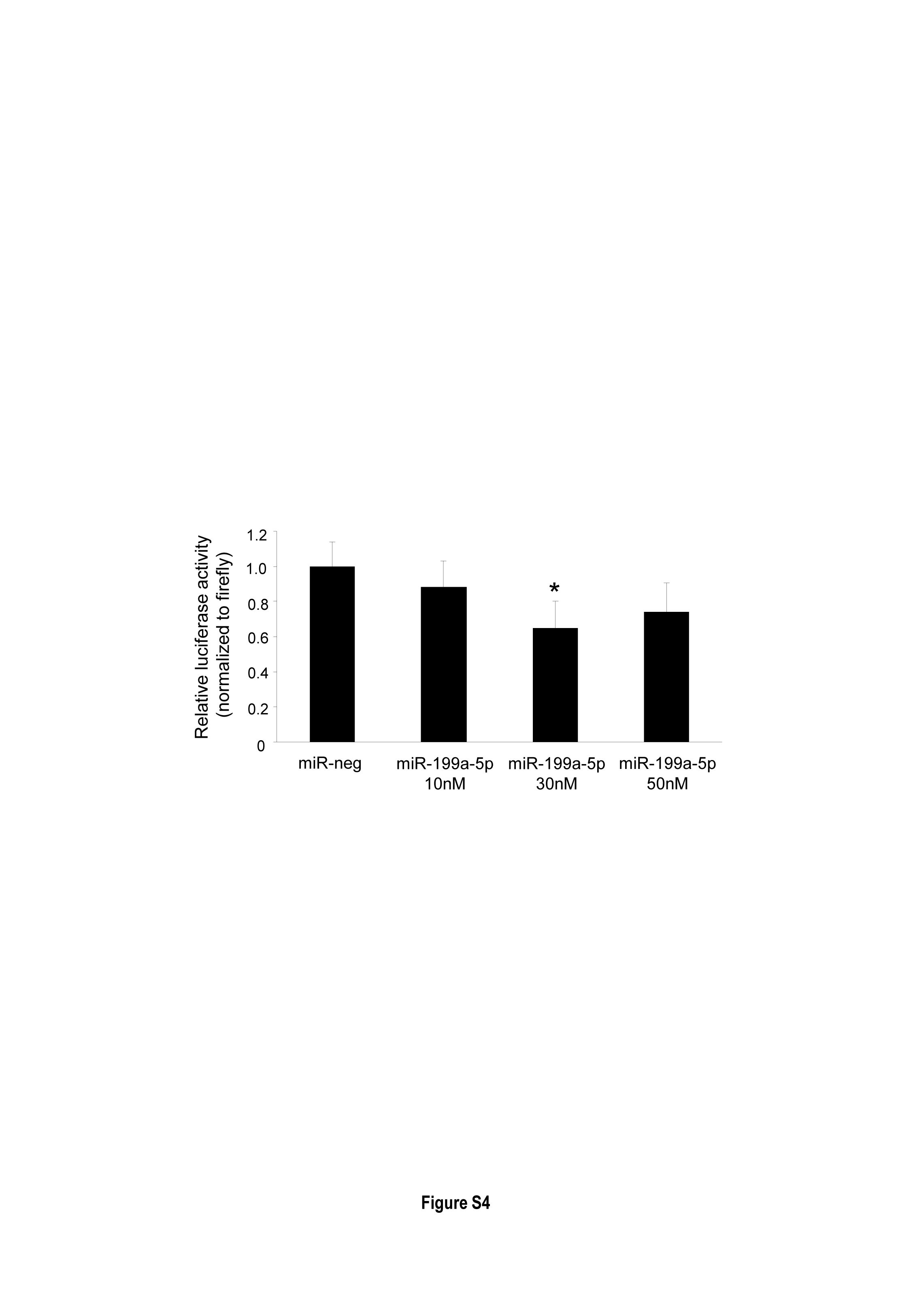

Supplement: Figure S4 — CAV1 is a direct target of miR-199a-5p. Co-transfection of pre-miR-199a-5p or pre-miR-Neg and human CAV1 3′UTR-derived psiCHECK-2 construct in A549 cells show a significant decrease in normalized luciferase activity 48 h post-transfection. * p<0.05. (TIF) [file pgen.1003291.s004.tif]

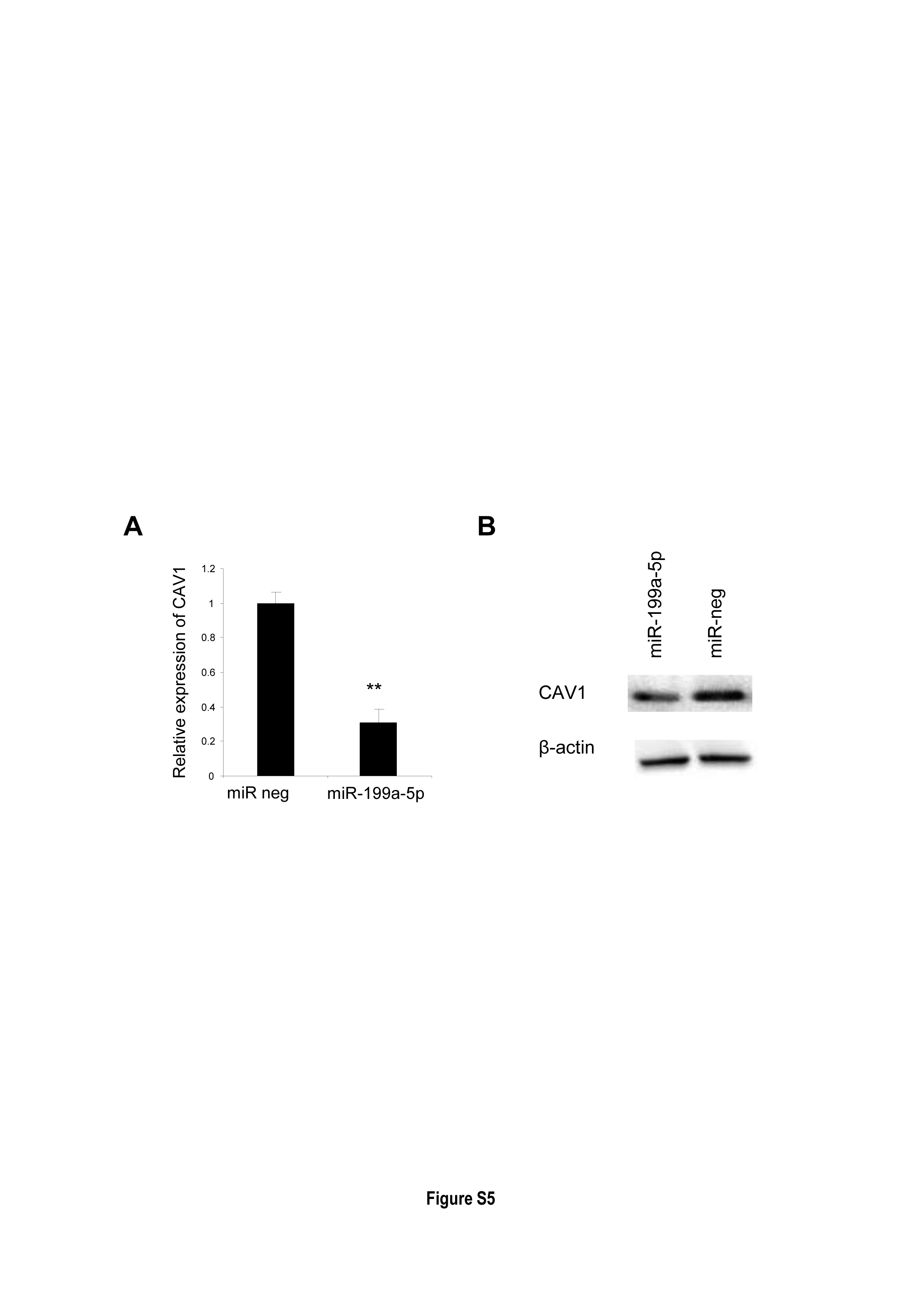

Supplement: Figure S5 — Decreased CAV1 expression after transfection of MRC5 lung fibroblasts with pre-miR-199a-5p. (A) MRC5 Lung fibroblasts that were transfected with 10 nM of pre-miR-199a-5p for 48 h show a significant decrease in CAV1 expression as determined by real-time PCR. Data are expressed as mean ± SEM. **p<0.01. (B) Western blot analysis showing the downregulated expression of CAV1 protein after transfection of MRC-5 lung fibroblasts with pre-miR-199a-5p. Data are representative of two independent experiments. (TIF) [file pgen.1003291.s005.tif]

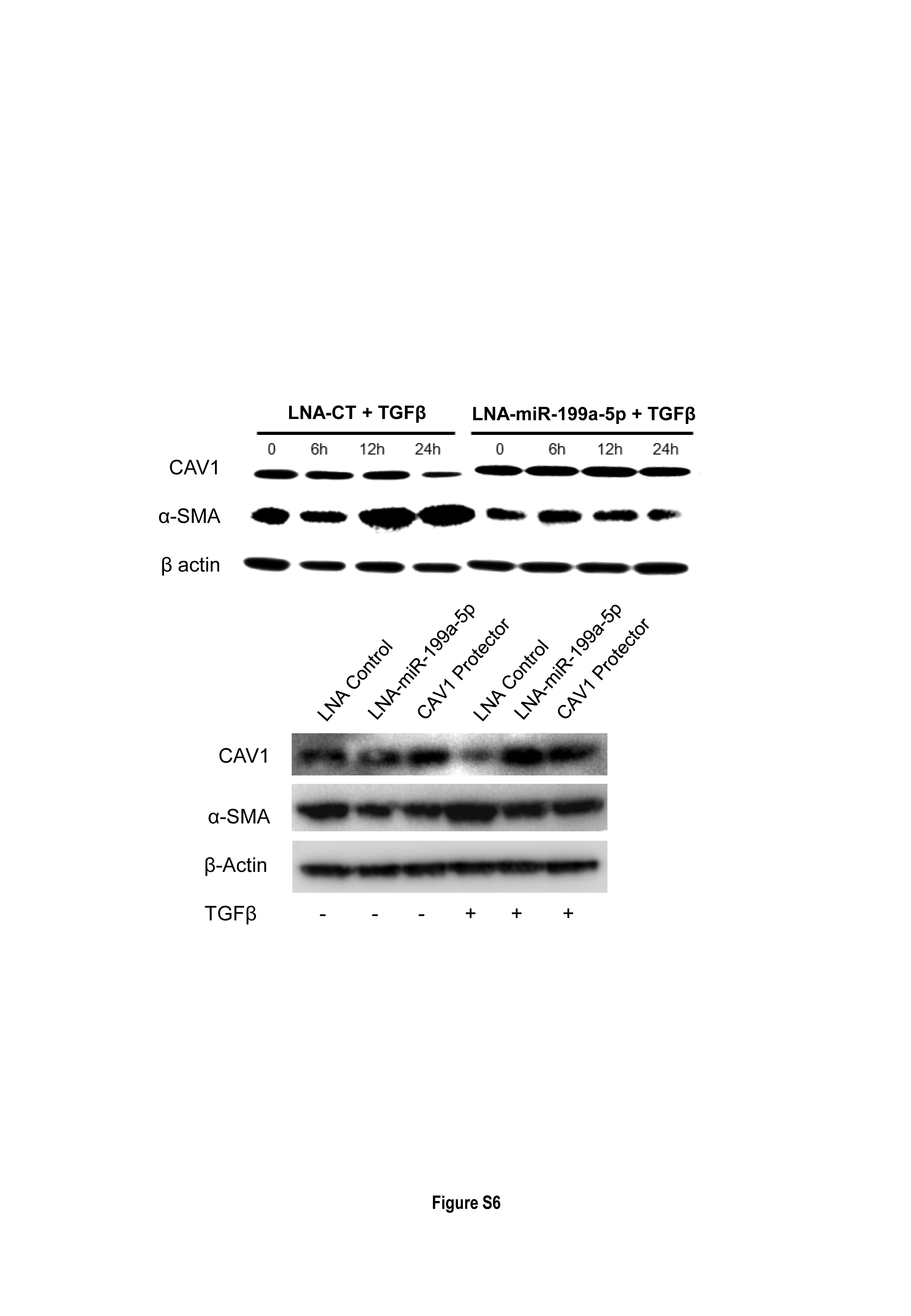

Supplement: Figure S6 — MiR-199a-5p mediates TGFβ dependent differentiation of lung fibroblast into myofibroblasts through CAV1 regulation. Normal human pulmonary fibroblasts MRC5 were transfected with a control LNA inhibitor (LNA-CT), a LNA-miR-199a-5p inhibitor or a target site blocker directed against CAV1 3-UTR (CAV1 protector) (n = 2). Protein samples were harvested at 48 h post-transfection and analyzed by western Blot for CAV1 and αSMA. (TIF) [file pgen.1003291.s006.tif]

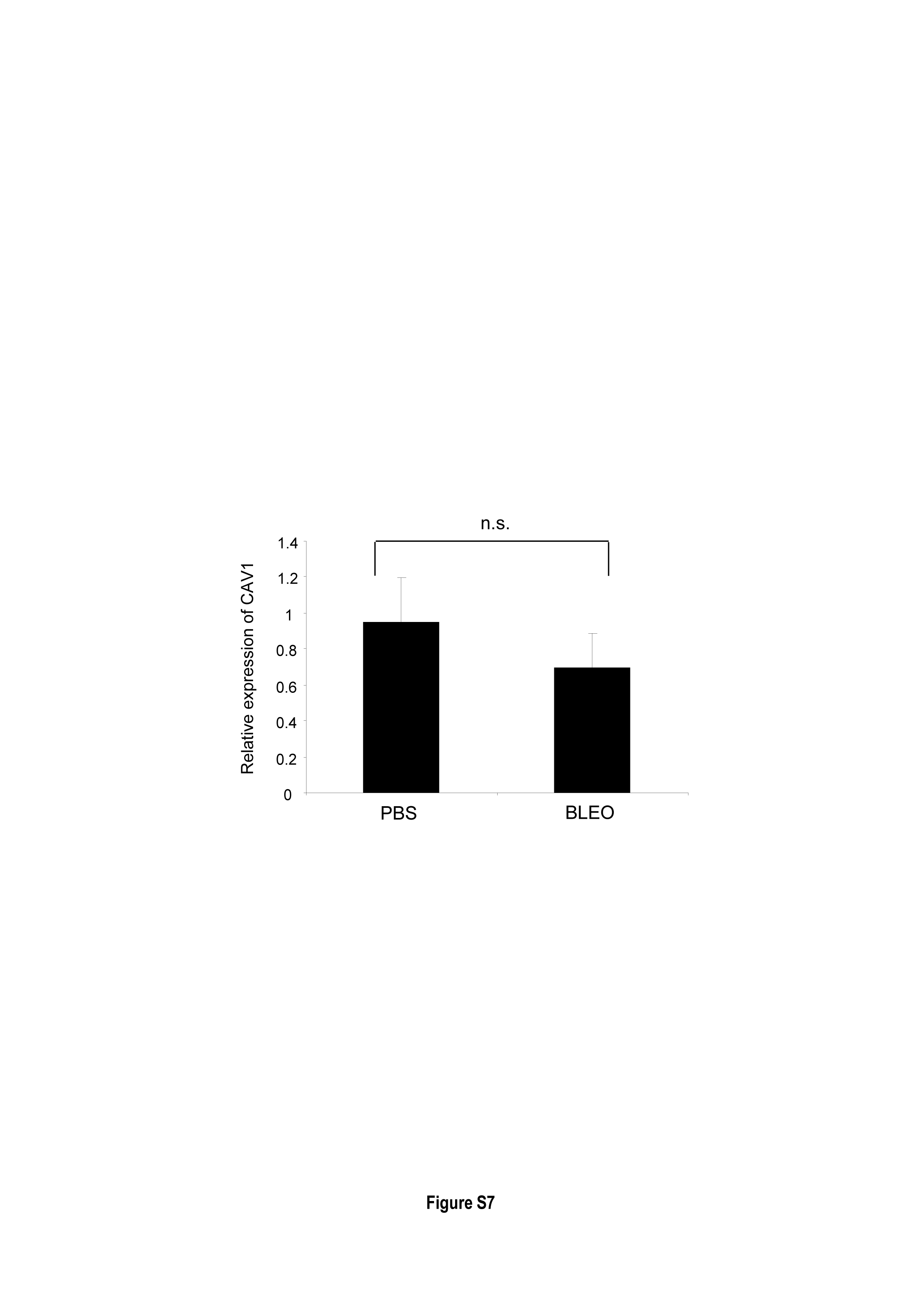

Supplement: Figure S7 — Pulmonary expression of CAV1 in BALB/c mice 14 days after bleomycin injection. Real-time PCR was performed to assess the expression of CAV1 in lungs of BALB/c mice 14 days following bleomycin exposure. n = 5 mice in each group, data are expressed as mean ± SEM. n.s. = non significant. (TIF) [file pgen.1003291.s007.tif]

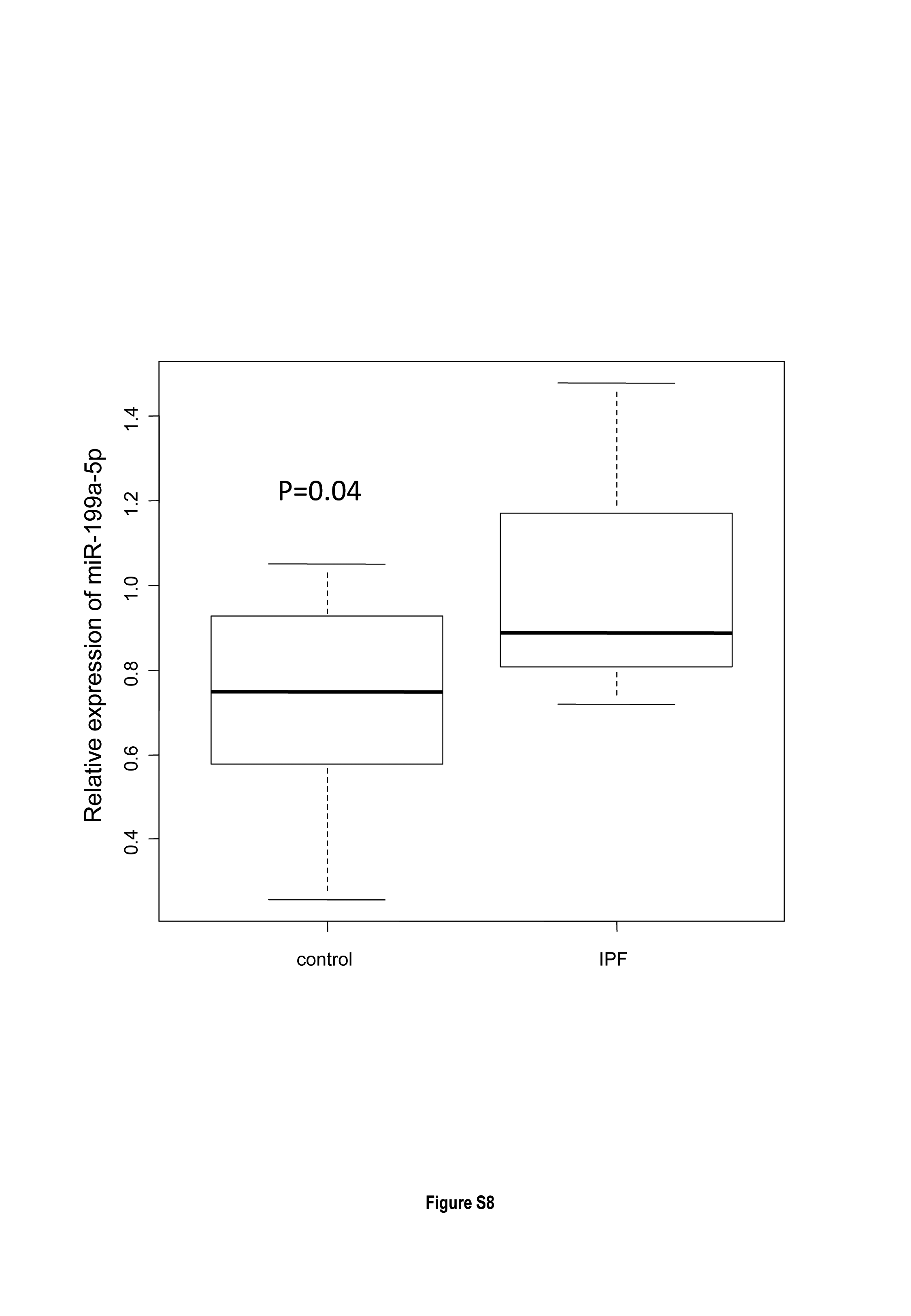

Supplement: Figure S8 — MiR-199a-5p expression determined by qPCR using FFPE lung samples. Box plot showing the increased expression of miR-199a-5p in IPF samples (n = 10) compared to control (n = 10). Significance was evaluated using the Kruskal–Wallis rank-sum test. The box represents the 25–75% quartiles, the line in the box represents the median and whiskers represent the range. (TIF) [file pgen.1003291.s008.tif]

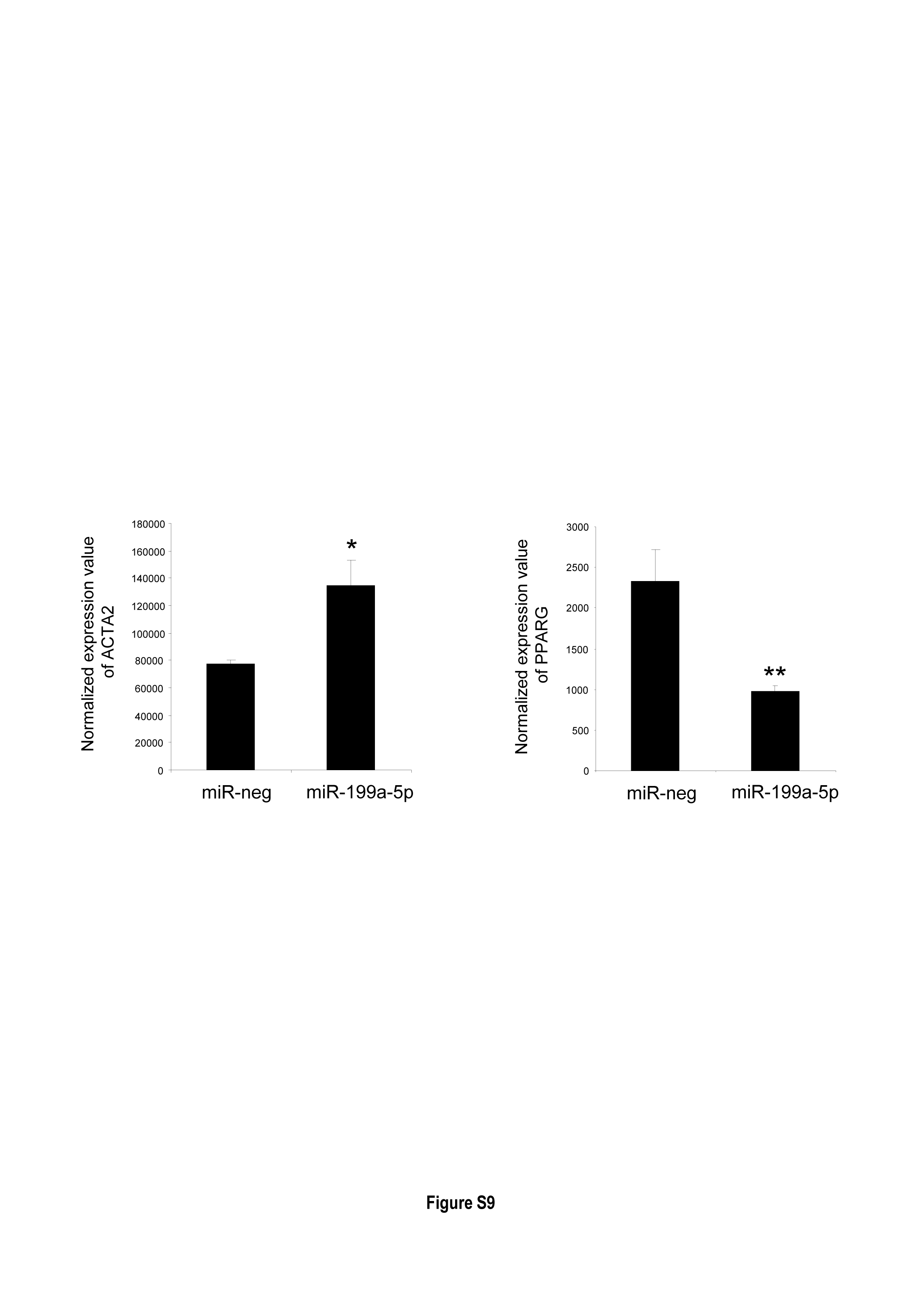

Supplement: Figure S9 — Expression of ACTA2 and PPARG following overexpression of miR-199a-5p in lung fibroblasts. Microarray analysis of lung fibroblasts transfected with 10 nM of miR-199a-5p mimic or miR-Neg reveals a significant increase of ACTA2 expression (*p<0.05), a hallmark of myofibroblast differentiation, as well as a significant decrease of PPARG expression (**p<0.01), a known inhibitor of myofibroblast differentiation. Data are expressed as mean of normalized fluorescence values ± SEM. (TIF) [file pgen.1003291.s009.tif]

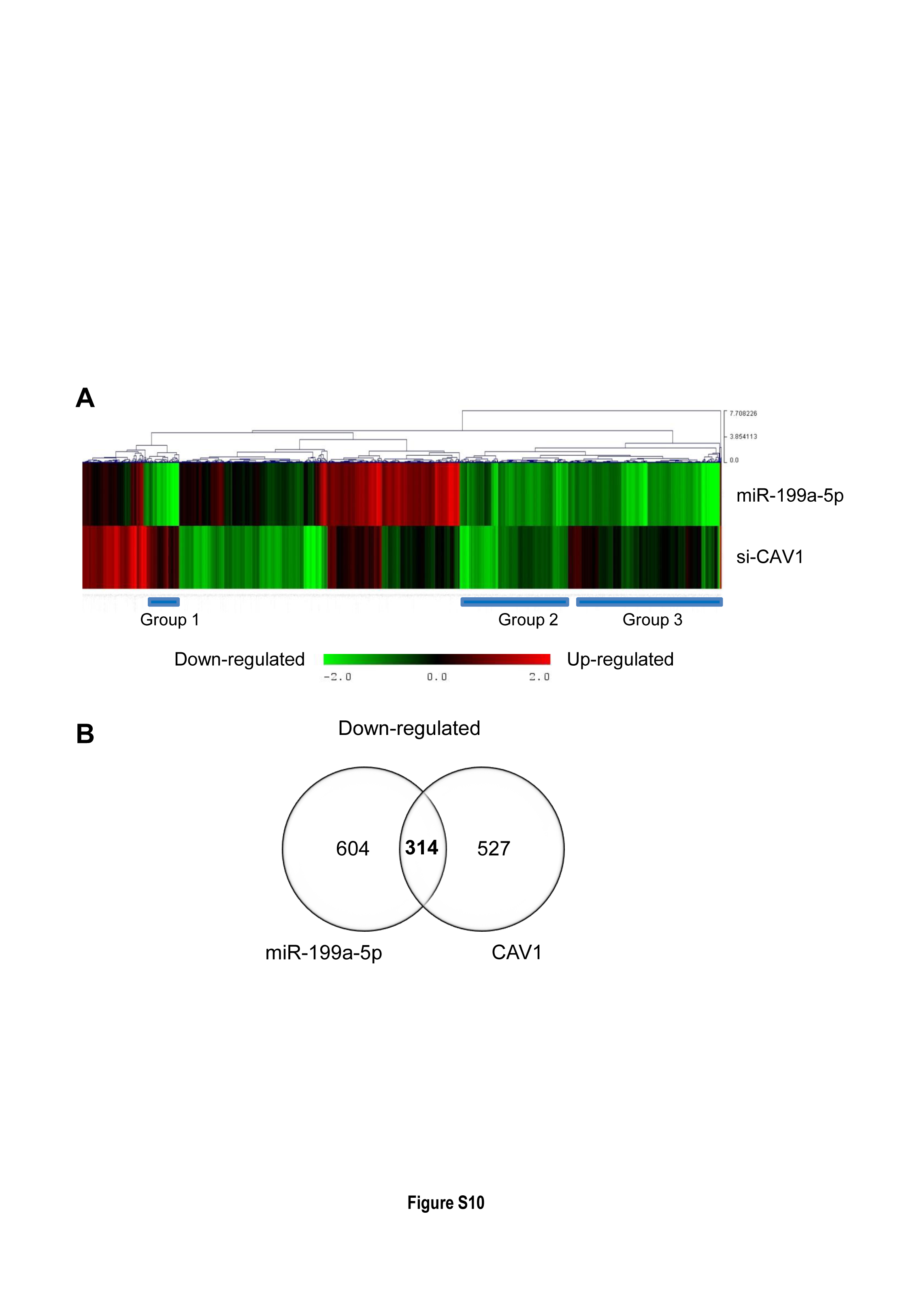

Supplement: Figure S10 — Comparison of transcriptomic changes induced by miR-199a-5p and a siRNA directed against CAV1. Normal human pulmonary fibroblasts hFL1 were transfected with pre-miR-Neg, pre-miR-199a-5p as well as siCAV1 or a control siRNA (n = 2). RNA samples were harvested at 48 h post-transfection and expression profiles were determined with pan genomic arrays. (A) Heatmap comparing the normalized log2 of the ratios between pre-miR-199a-5p versus pre-miR-Neg or siCAV1 versus siNeg signals. (B) Venn diagram comparing the set of down-regulated transcripts following miR-199a-5p and siCAV1. Cut-offs for selection are equal to 7.0 for the log2 (signal), 0.7 for the log2 (ratio), and 0.05 for the adjusted p-value. (TIF) [file pgen.1003291.s010.tif]

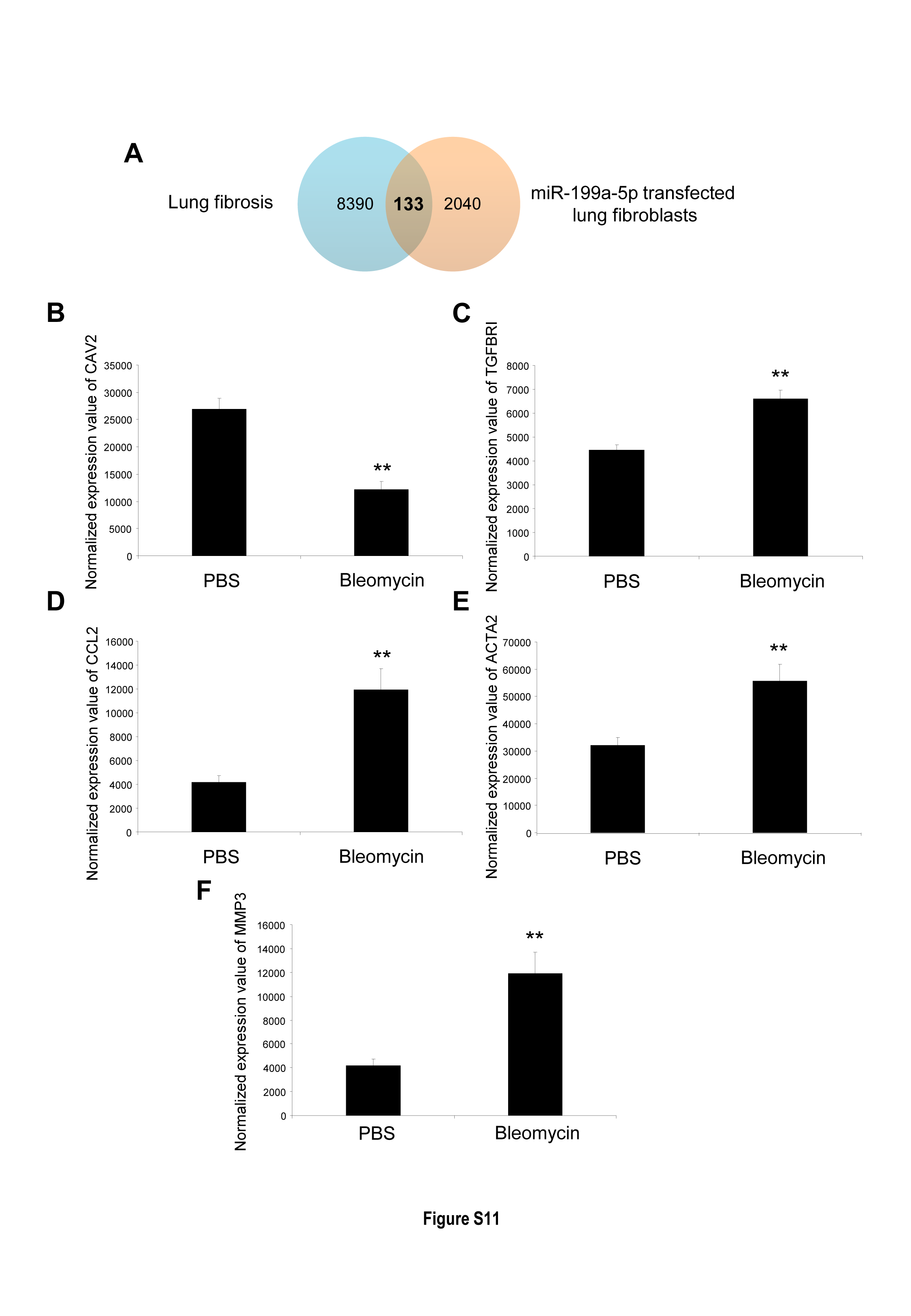

Supplement: Figure S11 — Comparison of gene expression changes between miR-199a-5p-regulated genes in hFL1 human lungs fibroblasts and lungs from C57BL/6 mice 14 days after bleomycin injection. (A) Venn diagram showing the relationships of gene expression changes between miR-199a-5p transfected lung fibroblasts (two independent experiments) and lungs from C57BL/6 14 days after Bleomycin treatment (n = 5 mice). The numbers of genes whose expression was differentially detected in each condition at p<0.05 are shown. Microarray analysis shows a significant reduction of CAV2 (B), TGFBRI (C), CCL2 (D), ACTA2 (E) and MMP3 (F) expression in C57BL/6 mice treated with bleomycin for 14 days (n = 5) compared with control mice (n = 5). Data are expressed as mean ± SEM. ** p<0.01. (TIF) [file pgen.1003291.s011.tif]

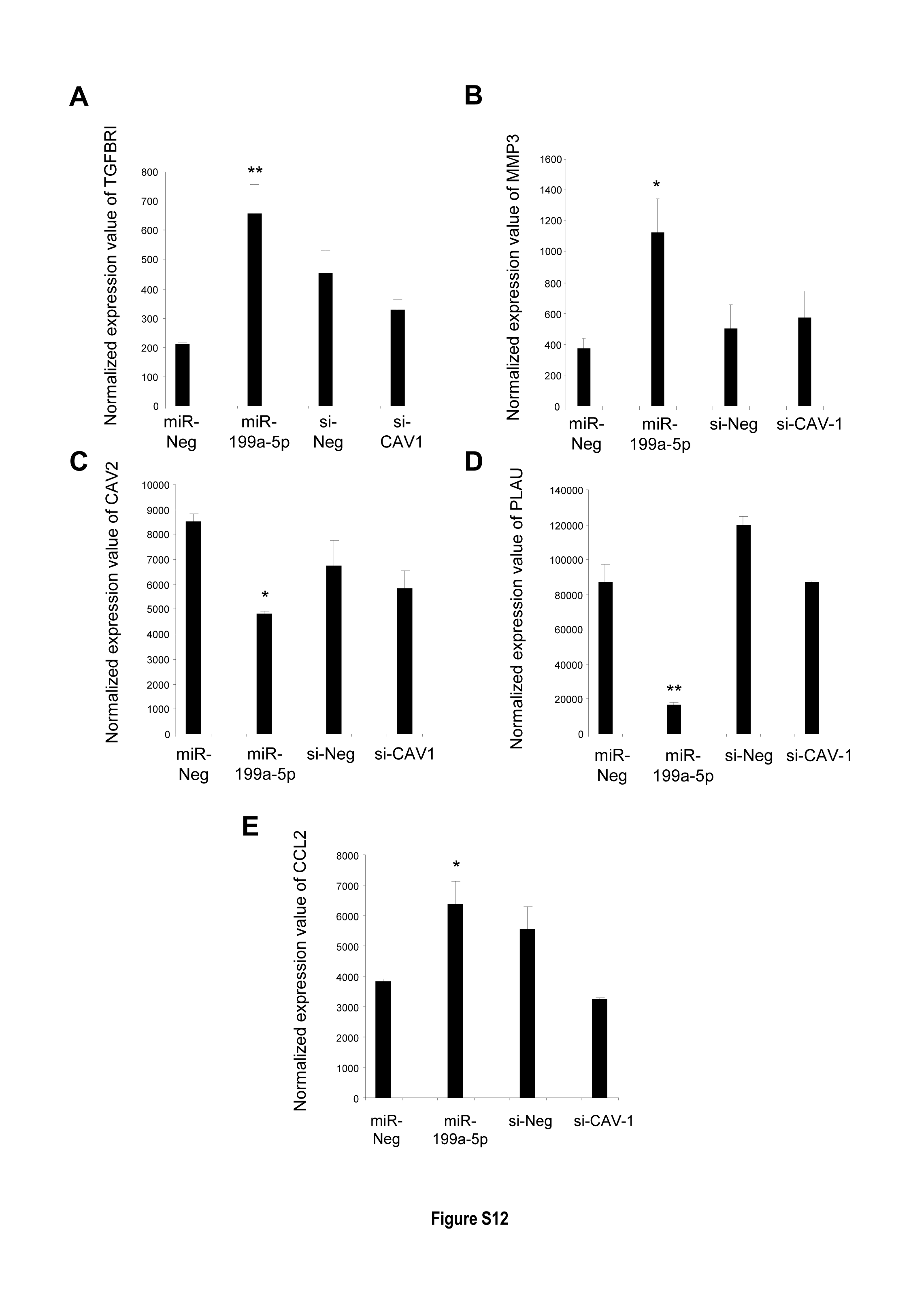

Supplement: Figure S12 — Profibrotic genes significantly modulated in lung fibroblasts by miR-199a-5p independently of CAV1 regulation. Lung fibroblasts were transfected by miR-199a-5p mimic, si-CAV1 or negative controls. Microarray analysis shows the expression of known profibrotic genes: TGFBRI (A), MMP3 (B), CAV2 (C), PLAU (D) and CCL2 (E) 48 h after transfection. Data are expressed as mean ± SEM. *p<0.05, **p<0.01. (TIF) [file pgen.1003291.s012.tif]

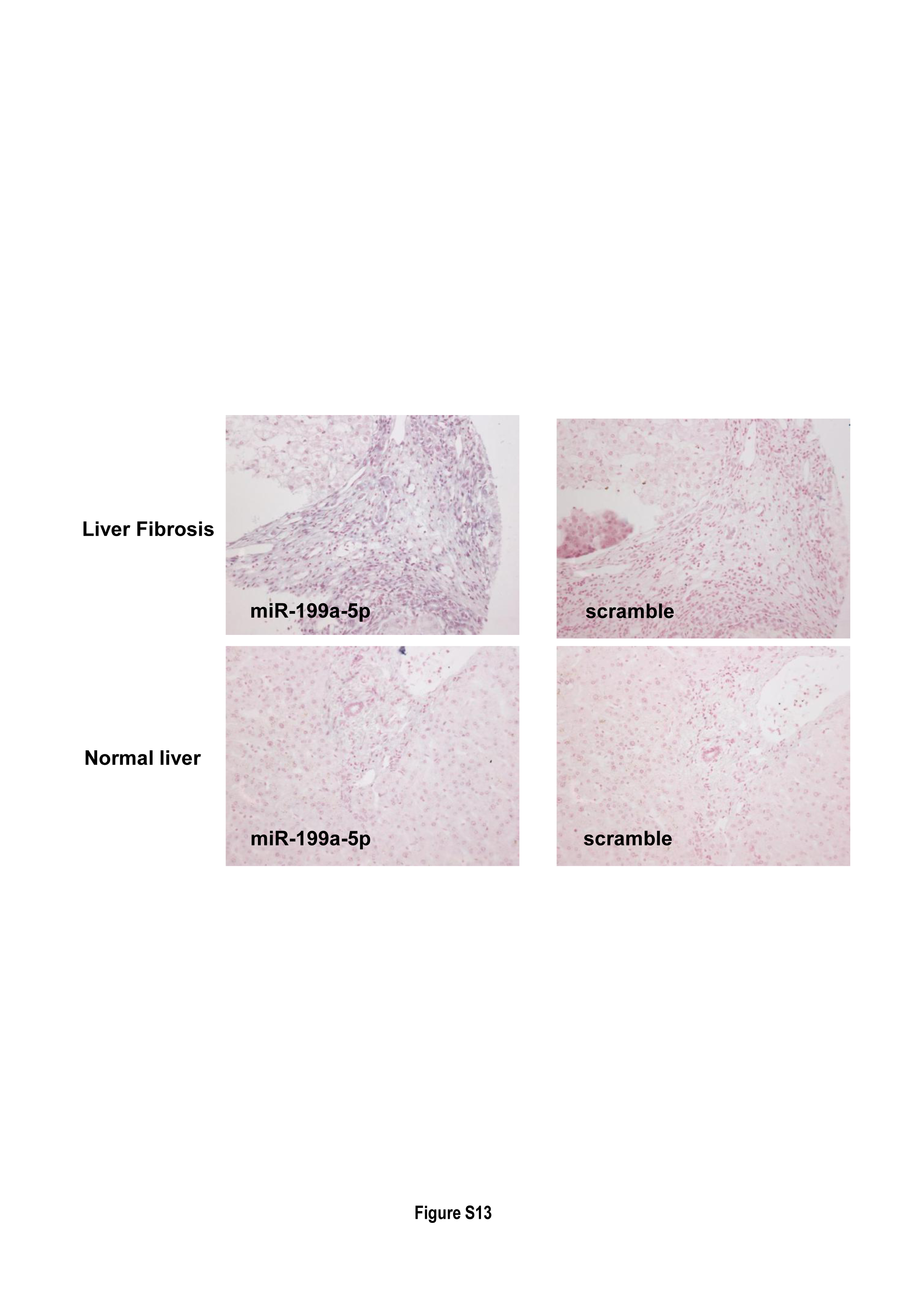

Supplement: Figure S13 — Enhanced expression of miR-199a-5p in clinical samples from patients with liver fibrosis. In situ hybridization assay was performed to determine the localization of miR-199a-5p in normal and fibrotic human livers. Results represent one out of three independent experiments. (TIF) [file pgen.1003291.s013.tif]

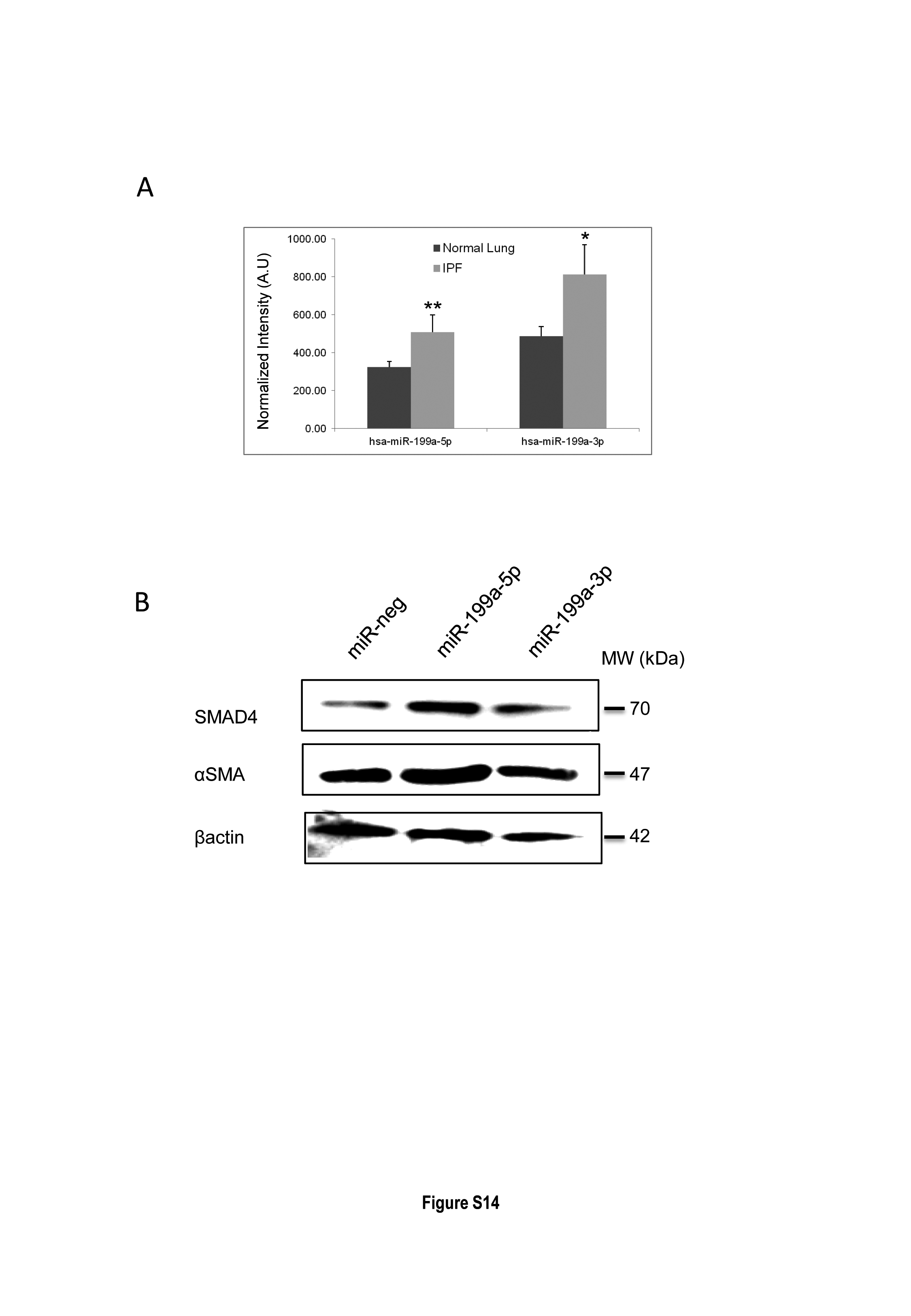

Supplement: Figure S14 — Expression of miR-199a-3p in lung fibrosis and impact of its overexpression on pulmonary fibroblast differentiation. (A) Increased expression of miR-199a-3p and miR-199a-5p in lung samples from IPF patients (n = 10) compared to control lung (n = 10). The mean normalized fluorescence intensity for the agilent probe is displayed; (B) Western blot analysis showing the effect of miR-199-5p or miR-199a-3p overexpression in hFL1 lung fibroblasts on SMAD4 and αSMA expression. One representative experiment out of two is shown. (TIF) [file pgen.1003291.s014.tif]

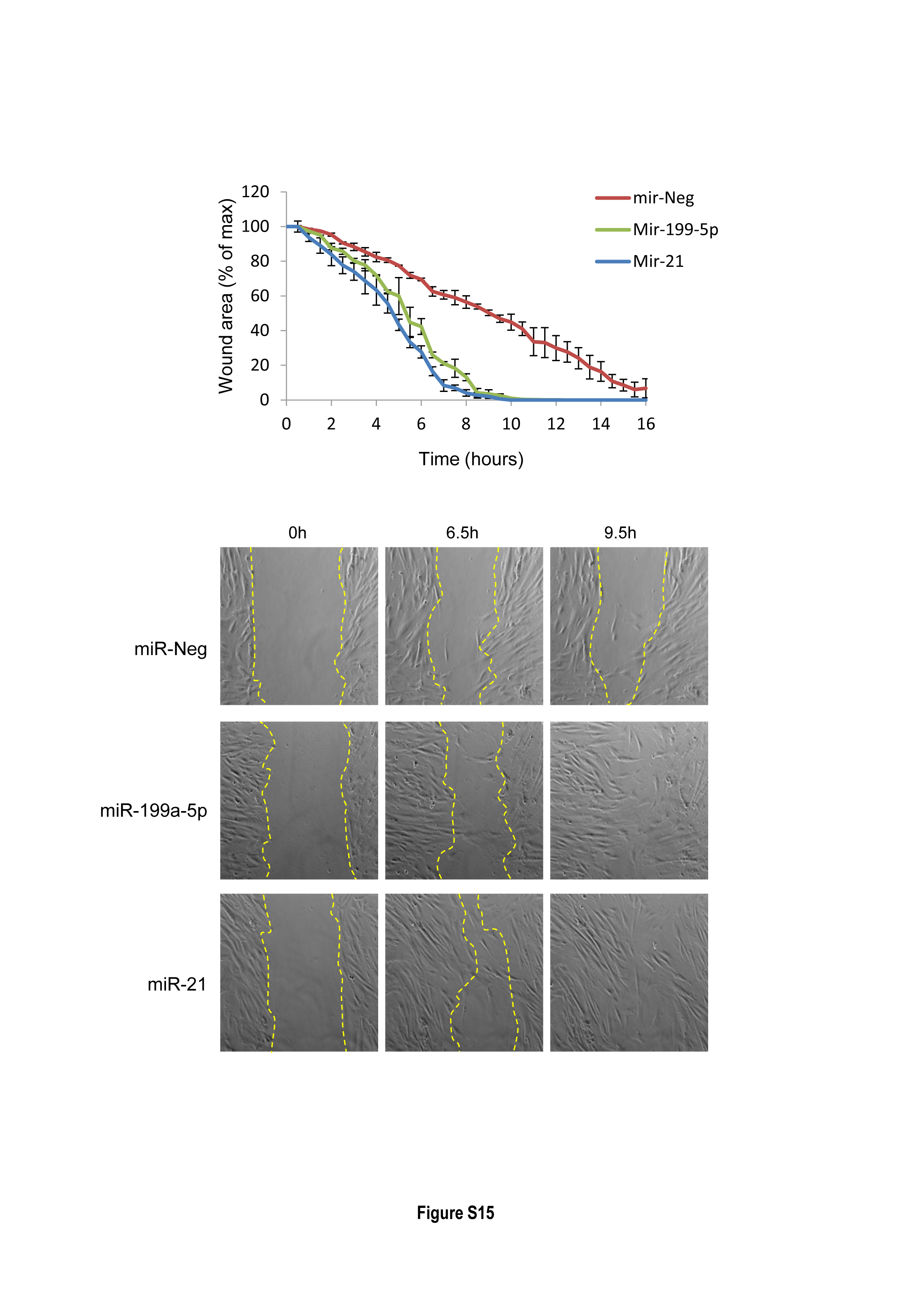

Supplement: Figure S15 — Transfection of human lung fibroblasts with pre-miR-199a-5p or pre-miR-21 increases cell motility. Sratch wound was induced in confluent cell monolayers plated on plastic and wound closure was measured using image J software (2 independent experiments). (TIF) [file pgen.1003291.s015.tif]

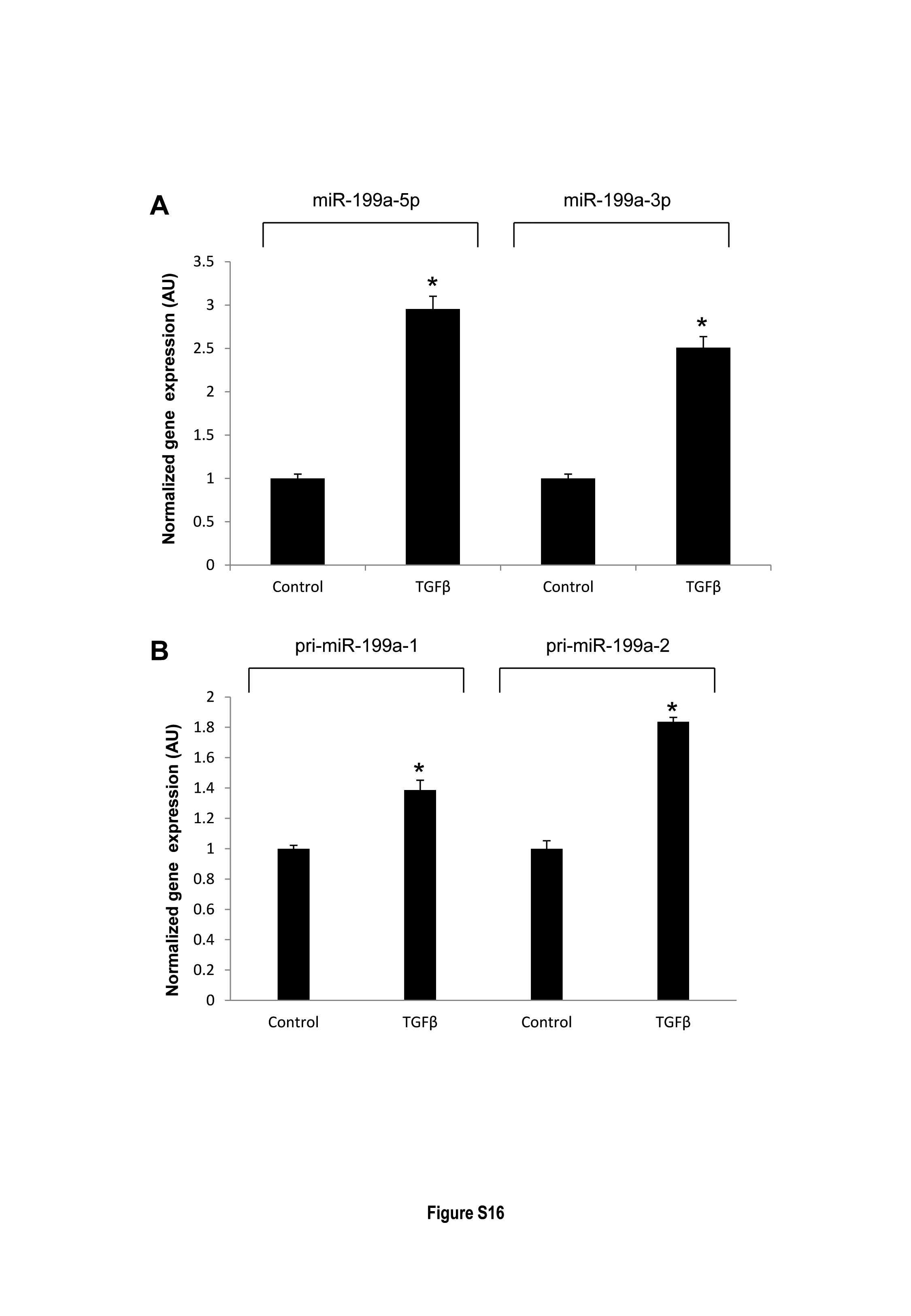

Supplement: Figure S16 — Effect of TGFβ on mature and pri-miRNAs forms of miR-199a in human lung fibroblasts. HFL1 human fibroblasts were treated or not with 10 nM TGFβ for 48 hours. Real Time TaqMan PCR showing the levels of (A) mature miR-199a-5p and miR-199a-3p; (B) pri-miR-199a-1 and pri-miR-199a-2. n = 2. Data are expressed as mean ± SEM *p<0.05. (TIF) [file pgen.1003291.s016.tif]
