# Supplementary material for: miR-199a-5p Is Upregulated during Fibrogenic Response to Tissue Injury and Mediates TGFbeta-Induced Lung Fibroblast Activation by Targeting Caveolin-1
Source: PLoS Genet. 2013 Feb 14;9(2):e1003291. doi: 10.1371/journal.pgen.1003291 (PMC3573122; doi:10.1371/journal.pgen.1003291)
Supplement: Table S1 — List of themes corresponding to “canonical pathways” annotations identified by Ingenuity Pathway Analysis in response to overexpression of miR-199a-5p or miR-21 in human pulmonary fibroblasts hFL1. Normal human pulmonary fibroblasts hFL1 were transfected with pre-miR-Neg, pre-miR-199a-5p or pre-miR-21 (n = 2). RNA samples were harvested at 48 h post-transfection and expression profiles were determined with whole genome microarrays. The probability to obtain the number of genes in a certain pathway in the list of differentially expressed genes between either miR-199a-5p or miR-21 versus miR-Neg was compared with the representation of the same pathway among all the genes on the microarray; −log10 of the Fisher's exact probability is indicated. Significant pathways are shown in progressively brighter shades of orange according to their significance. ns = non significant. (DOCX) [file pgen.1003291.s017.docx]

| **Ingenuity Canonical Pathways** | **miR-199a-5p** | **miR-21** |
| --- | --- | --- |
| Acute Phase Response Signaling | 2.39 | ns |
| April Mediated Signaling | 2.52 | ns |
| ATM Signaling | ns | 4.69 |
| Biosynthesis of Steroids | 6.94 | ns |
| Caveolar-mediated Endocytosis Signaling | 1.46 | ns |
| Cell Cycle Control of Chromosomal Replication | ns | 6.84 |
| Cell Cycle: G1/S Checkpoint Regulation | 4.06 | 3.71 |
| Cell Cycle: G2/M DNA Damage Checkpoint Regulation | ns | 7.71 |
| Cyclins and Cell Cycle Regulation | 3.03 | 6.55 |
| Death Receptor Signaling | 1.74 | 1.21 |
| DNA Double-Strand Break Repair by Homologous Recombination | ns | 2.86 |
| Fatty Acid Metabolism | 1.31 | ns |
| GNRH Signaling | 1.74 | ns |
| IL-1 Signaling | 2.63 | ns |
| IL-10 Signaling | 2.13 | ns |
| IL-12 Signaling and Production in Macrophages | 2.28 | ns |
| IL-15 Signaling | 1.59 | ns |
| IL-17A Signaling in Airway Cells | 2.16 | ns |
| IL-17A Signaling in Fibroblasts | ns | 2.75 |
| IL-6 Signaling | 1.89 | ns |
| Integrin Signaling | 1.87 | ns |
| LPS-stimulated MAPK Signaling | 2.45 | ns |
| Mismatch Repair in Eukaryotes | ns | 3.96 |
| Mitotic Roles of Polo-Like Kinase | 1.22 | 9.15 |
| Molecular Mechanisms of Cancer | 1.74 | 1.41 |
| NF-κB Signaling | 3.03 | ns |
| Nur77 Signaling in T Lymphocytes | 1.57 | ns |
| p38 MAPK Signaling | 2.79 | ns |
| p53 Signaling | 1.78 | ns |
| Pantothenate and CoA Biosynthesis | 1.71 | ns |
| Parkinson's Signaling | 2.04 | ns |
| Phospholipase C Signaling | 1.57 | ns |
| PKC Signaling in T Lymphocytes | 2.12 | ns |
| PPAR Signaling | 1.53 | ns |
| Production of Nitric Oxide and Reactive Oxygen Species in Macrophages | 1.68 | ns |
| Protein Ubiquitination Pathway | ns | 1.50 |
| PTEN Signaling | 2.70 | ns |
| Purine Metabolism | 1.69 | ns |
| Pyrimidine Metabolism | 1.52 | 1.19 |
| Role of BRCA1 in DNA Damage Response | ns | 5.61 |
| Role of CHK Proteins in Cell Cycle Checkpoint Control | ns | 6.07 |
| Role of JAK family kinases in IL-6-type Cytokine Signaling | 1.60 | ns |
| Role of NFAT in Cardiac Hypertrophy | 2.65 | ns |
| Role of PKR in Interferon Induction and Antiviral Response | 1.68 | ns |
| Small Cell Lung Cancer Signaling | 2.45 | 2.22 |
| TGF-β Signaling | 2.22 | 1.41 |
| TWEAK Signaling | 2.04 | 1.07 |
| Type I Diabetes Mellitus Signaling | 1.82 | ns |
| Type II Diabetes Mellitus Signaling | 2.14 | ns |
| Valine, Leucine and Isoleucine Degradation | 1.56 | ns |
| Wnt/β-catenin Signaling | 2.41 | ns |
