# Supplementary material for: miR-199a-5p Is Upregulated during Fibrogenic Response to Tissue Injury and Mediates TGFbeta-Induced Lung Fibroblast Activation by Targeting Caveolin-1
Source: PLoS Genet. 2013 Feb 14;9(2):e1003291. doi: 10.1371/journal.pgen.1003291 (PMC3573122; doi:10.1371/journal.pgen.1003291)
Supplement: Table S2 — List of themes corresponding to “canonical pathways” annotations identified by Ingenuity Pathway Analysis in lungs from C57BL/6 mice treated with bleomycin. RNAs extracted from C57BL/6 mice lungs 14 days after instillation with bleomycin or PBS were analyzed with whole genome microarrays (n = 5).The probability to obtain the number of genes in a certain pathway in the list of differentially expressed genes between bleomycin and PBS conditions was compared with the representation of the same pathway among all the genes on the microarray; −log10 of the Fisher's exact probability is indicated. (DOCX) [file pgen.1003291.s018.docx]

| **Ingenuity Canonical Pathways** | **-log(p-value)** | **Molecules** |
| --- | --- | --- |
| Hepatic Fibrosis / Hepatic Stellate Cell Activation | 6.63 | CCR5, CTGF, FN1, EDNRB, LEPR, FGFR2, MMP13, MYH7, MMP2, IL6, PDGFC, PGF, FGF1, VEGFA, CCL2, IGF1, TIMP1, KDR, IGFBP3, LAMA1, TNFRSF1B, COL3A1 |
| Bladder Cancer Signaling | 3.98 | CDKN2A, MMP14, MMP15, MMP10, MMP13, MMP2, PDGFC, PGF, FGF1, VEGFA, MMP8, CDKN1A, MMP12 |
| Complement System | 3.66 | CFB, CFI, C1QC, C1QA, C5, C1QB, C3AR1 |
| LPS/IL-1 Mediated Inhibition of RXR Function | 3.47 | GSTA3, PPARA, APOE, GAL3ST2, GSTM5, MGMT, IL4I1, APOC2, CHST1, MAOB, Gstm3, CAT, SLC27A6, GSTM4, FABP1, FABP7, FMO1, PLTP, CYP2B6, TNFRSF1B |
| Cell Cycle: G2/M DNA Damage Checkpoint Regulation | 3.23 | CDKN2A, CDKN1A, CKS1B, TOP2A, CCNB2, PLK1, SFN, CDK1 |
| Atherosclerosis Signaling | 3.11 | MSR1, CCL2, CXCL12, LPL, PLA2G1B, MMP13, IL6, CCR2, COL18A1, PLA2G7, PDGFC, COL3A1 |
| Role of Macrophages, Fibroblasts and Endothelial Cells in Rheumatoid Arthritis | 2.83 | TLR1, SOCS3, WNT10B, FN1, FRZB, NFKBIE, MMP13, IL6, PLCH2, PDGFC, FCGR1A, PGF, VEGFA, ROR2, CCL2, DKK2, TNFRSF1B, NOS2, FCGR3A, ADAMTS4, C5, WNT10A, CXCL12, Tlr13, SFRP1 |
| LXR/RXR Activation | 2.73 | APOE, MSR1, CCL2, CCL7, LPL, APOC2, PLTP, IL6, TNFRSF1B, NOS2 |
| Role of Osteoblasts, Osteoclasts and Chondrocytes in Rheumatoid Arthritis | 2.66 | ACP5, CTSK, WNT10B, SPP1, FRZB, NFKBIE, MMP14, SMAD6, MMP13, IL6, IGF1, WNT10A, MMP8, CALCR, DKK2, SFRP1, TNFRSF1B, BMP6, BMP1, ADAMTS4 |
| Mitotic Roles of Polo-Like Kinase | 2.35 | CDC20, PLK3, PPM1L, PRC1, CCNB2, PLK1, Ccnb1/Gm5593, CDK1 |
| HIF1 Signaling | 2.29 | VEGFA, MMP14, MMP8, MMP15, MMP10, MMP13, MMP2, MMP12, NOS2, PDGFC, PGF |
| Role of IL-17F in Allergic Inflammatory Airway Diseases | 2.13 | CXCL10, IGF1, CCL2, CCL7, MMP13, IL6 |
| TREM1 Signaling | 2.11 | TLR1, CCL2, CCL7, Tlr13, CD86, IL6, FCGR2B |
| Role of Pattern Recognition Receptors in Recognition of Bacteria and Viruses | 2.09 | PTX3, TLR1, C1QC, C1QA, C5, C1QB, IL6, Oas1f, C3AR1 |
| Eicosanoid Signaling | 1.81 | PTGS1, PLA2G1B, FPR2, HPGDS, TBXAS1, PLA2G7, DPEP2 |
| Acute Phase Response Signaling | 1.76 | SOCS3, ITIH3, FN1, NFKBIE, SAA2, SERPINA3, C5, IL6, HMOX1, ITIH4, CFB, TNFRSF1B, SERPINE1, SAA1 |
| Dendritic Cell Maturation | 1.74 | FCGR2A, LEPR, NFKBIE, CD86, IL6, FCGR2B, TNFRSF1B, COL18A1, TREM2, FCGR1A, FCGR3A, COL3A1 |
| G-Protein Coupled Receptor Signaling | 1.72 | CCR5, HTR2B, NFKBIE, PDE1A, PDE4D, ADRB3, ADRB1, GPR116, CCRL2, GPR37, GPR85, CCRL1, CCR2, BAI2, DARC, APLNR, S1PR5, EDNRB, GPR84, FPR2, RGS16, RGS4, GPR176, CD97, P2RY14, CXCR7, P2RY6, PTH1R, GPR65, CALCR, PDE8B, EMR1, C3AR1 |
| IL-17A Signaling in Fibroblasts | 1.72 | CCL2, CCL7, NFKBIE, LCN2, IL6 |
| p53 Signaling | 1.69 | CDKN2A, PLAGL1, GADD45G, CDKN1A, SERPINB5, PIDD, SFN, BIRC5, SERPINE2 |
| Colorectal Cancer Metastasis Signaling | 1.69 | TLR1, WNT10B, ADRBK2, MMP14, MMP15, MMP10, MMP13, MMP2, IL6, PDGFC, PGF, BIRC5, VEGFA, WNT10A, MMP8, Tlr13, MMP12, NOS2 |
| IL-10 Signaling | 1.49 | HMOX1, SOCS3, CCR5, FCGR2A, NFKBIE, IL6, FCGR2B |
| Role of Hypercytokinemia/hyperchemokinemia in the Pathogenesis of Influenza | 1.47 | CXCL10, CCR5, CCL2, IL6 |
| Intrinsic Prothrombin Activation Pathway | 1.47 | KNG1, F10, COL18A1, COL3A1 |
| Airway Pathology in Chronic Obstructive Pulmonary Disease | 1.42 | MMP8, MMP2 |
| Aryl Hydrocarbon Receptor Signaling | 1.42 | CDKN2A, GSTA3, CTSD, CCNA2, CCNE1, GSTM5, TFF1, Gstm3, CDKN1A, GSTM4, IL6 |
| Serotonin Receptor Signaling | 1.37 | MAOB, HTR2B, IL4I1, SLC6A4 |
| Xenobiotic Metabolism Signaling | 1.33 | GSTA3, GAL3ST2, GSTM5, MGMT, IL4I1, IL6, Ces1e, HMOX1, CHST1, MAOB, Gstm3, CAT, PPM1L, CAMK1G, GSTM4, FMO1, CYP2B6, NOS2 |
| Pathogenesis of Multiple Sclerosis | 1.30 | CXCL10, CCR5 |
| Pancreatic Adenocarcinoma Signaling | 1.29 | VEGFA, CDKN2A, HMOX1, CCNE1, PLD3, CDKN1A, PDGFC, PGF, BIRC5 |
| Tryptophan Metabolism | 1.27 | BDH2, CYP2F1, MAOB, CYP7B1, MGMT, CAT, IL4I1, CYP2B6, INMT, Aox3 |
| Cyclins and Cell Cycle Regulation | 1.24 | CDKN2A, CCNA2, CCNE1, PPM1L, CDKN1A, CCNB2, CDK1 |
| Phospholipid Degradation | 1.19 | HMOX1, PLD3, PLA2G1B, DGKG, PLCH2, PLA1A, PLA2G7 |
| Nitrogen Metabolism | 1.15 | CA14, CA4, CA13, CA8 |
| T Helper Cell Differentiation | 1.15 | FOXP3, CD86, IL2RA, IL6, TNFRSF1B, GATA3 |
| Glutathione Metabolism | 1.10 | GSTA3, GSTM5, Gstm3, GSTM4, HPGDS |
| ATM Signaling | 1.07 | SMC2, GADD45G, CDKN1A, CCNB2, CDK1 |
| Communication between Innate and Adaptive Immune Cells | 1.05 | CXCL10, TLR1, Tlr13, CD86, IL6, Ccl9 |
| FXR/RXR Activation | 1.05 | PPARA, APOE, PON1, MLXIPL, FOXA1, APOC2, PLTP |
| Neuroprotective Role of THOP1 in Alzheimer's Disease | 1.05 | KNG1, PDYN, NTS, SERPINA3 |
| Role of IL-17A in Arthritis | 1.04 | CCL2, CCL7, NFKBIE, MMP13, NOS2 |
| Chemokine Signaling | 1.03 | CCR5, CCL13, CCL2, CCL7, CXCL12, CAMK1G |
