# Supplementary material for: miR-199a-5p Is Upregulated during Fibrogenic Response to Tissue Injury and Mediates TGFbeta-Induced Lung Fibroblast Activation by Targeting Caveolin-1
Source: PLoS Genet. 2013 Feb 14;9(2):e1003291. doi: 10.1371/journal.pgen.1003291 (PMC3573122; doi:10.1371/journal.pgen.1003291)
Supplement: Table S3 — Pulmonary expression of miR-199a-5p in 10 IPF and 10 controls (dataset GEO accession number GSE13316). (DOCX) [file pgen.1003291.s019.docx]

| **Probe ID** | **miRNA name** | **control^a^** | **IPF^a^** | **Ratio^b^** | **p-value^c^** |
| --- | --- | --- | --- | --- | --- |
| A_25_P00010700 | hsa-miR-199a-5p | 8.38 | 8.69 | 1.24 | 0.006 |
| A_25_P00010701 | hsa-miR-199a-5p | 6.31 | 6.69 | 1.30 | 0.005 |
| A_25_P00010069 | hsa-miR-199a-3p | 8.97 | 9.47 | 1.41 | 0.015 |
| A_25_P00010068 | hsa-miR-199a-3p | 7.89 | 8.19 | 1.23 | 0.045 |

^a^median expression; ^b^IPF vs.control; ^c^Wilcoxon rank sum test
