# Supplementary material for: miR-199a-5p Is Upregulated during Fibrogenic Response to Tissue Injury and Mediates TGFbeta-Induced Lung Fibroblast Activation by Targeting Caveolin-1
Source: PLoS Genet. 2013 Feb 14;9(2):e1003291. doi: 10.1371/journal.pgen.1003291 (PMC3573122; doi:10.1371/journal.pgen.1003291)
Supplement: Table S4 — List of the 133 genes modulated by miR-199a-5p in lung fibroblasts that are also dysregulated in lungs from C57BL/6 mice 14 days after bleomycin treatment. (DOCX) [file pgen.1003291.s020.docx]

|  | **miR-199a-5p transfected lung fibroblasts** | | | **bleomycin induced lung fibrosis** | | |
| --- | --- | --- | --- | --- | --- | --- |
| **Gene Symbol** | **Average intensity** | **Log Ratio** | **p-value** | **Average intensity** | **Log Ratio** | **p-value** |
| ADAMTS4 | 10.87 | 0.77 | 0.037205 | 9.02 | 3.17 | 0.000044 |
| AKAP7 | 9.57 | -1.59 | 0.002165 | 12.05 | -1.07 | 0.001094 |
| ANAPC13 | 12.52 | -1.22 | 0.001805 | 15.47 | -0.47 | 0.034385 |
| AOX1 | 11.46 | -1.69 | 0.000211 | 12.48 | -0.98 | 0.001395 |
| ARAP3 | 11.96 | -0.70 | 0.027772 | 10.49 | -1.28 | 0.000042 |
| ARHGAP12 | 11.29 | -2.00 | 0.010078 | 10.52 | -0.85 | 0.000414 |
| ASGR1 | 9.08 | -0.99 | 0.012113 | 9.12 | -3.12 | 0.000005 |
| ASPH | 13.01 | -1.43 | 0.029179 | 12.21 | -0.44 | 0.002034 |
| ATP9A | 13.11 | -1.20 | 0.008302 | 16.02 | -0.36 | 0.049043 |
| BTRC | 9.16 | -1.39 | 0.004120 | 10.11 | -0.54 | 0.014927 |
| CABYR | 10.57 | 0.94 | 0.041231 | 7.21 | 1.32 | 0.003024 |
| CACNB3 | 10.68 | 0.72 | 0.048016 | 10.44 | 0.97 | 0.000449 |
| CAV1 | 15.73 | -1.71 | 0.005572 | 11.76 | -1.03 | 0.002899 |
| CAV2 | 12.60 | -0.92 | 0.025258 | 14.01 | -1.20 | 0.001986 |
| CCNA2 | 11.73 | 0.94 | 0.022863 | 10.95 | 2.20 | 0.000330 |
| CCNB2 | 14.79 | 0.89 | 0.015840 | 11.52 | 1.84 | 0.000200 |
| CDH2 | 14.14 | -1.96 | 0.000123 | 10.48 | -0.54 | 0.027403 |
| CDKN1B | 11.99 | -0.84 | 0.018487 | 13.08 | -0.90 | 0.017326 |
| CDKN3 | 10.76 | 0.90 | 0.026616 | 8.65 | 1.88 | 0.000218 |
| CDT1 | 12.14 | 1.00 | 0.038265 | 11.65 | 0.87 | 0.005190 |
| CENPA | 11.43 | 0.83 | 0.045218 | 10.73 | 1.23 | 0.003830 |
| CENPH | 10.83 | 1.06 | 0.011271 | 8.43 | 1.71 | 0.000086 |
| CENPN | 13.62 | 0.76 | 0.023514 | 7.52 | 1.06 | 0.015405 |
| CHAF1A | 13.01 | 0.91 | 0.010525 | 9.30 | 0.81 | 0.003431 |
| CHCHD10 | 11.89 | -1.15 | 0.015618 | 16.97 | -0.92 | 0.001257 |
| CHTF18 | 11.97 | 0.67 | 0.048768 | 9.41 | 1.02 | 0.014577 |
| CTF1 | 10.88 | -0.69 | 0.039451 | 10.65 | -0.41 | 0.025602 |
| CTNND1 | 10.78 | -0.74 | 0.023539 | 15.01 | -0.72 | 0.002786 |
| CYB5B | 8.30 | -0.94 | 0.023676 | 13.70 | -0.72 | 0.009805 |
| CYP2S1 | 7.54 | -2.00 | 0.000431 | 15.16 | -0.79 | 0.002440 |
| CYTL1 | 7.96 | -1.23 | 0.008807 | 11.05 | -1.54 | 0.000494 |
| DALRD3 | 10.58 | -0.86 | 0.040702 | 9.06 | -0.61 | 0.037489 |
| DDX58 | 9.73 | -1.72 | 0.014459 | 9.22 | -0.62 | 0.035363 |
| DECR1 | 12.69 | -0.79 | 0.042105 | 9.29 | -0.66 | 0.015693 |
| EIF2S1 | 11.42 | 1.73 | 0.016695 | 12.95 | 0.53 | 0.029109 |
| ELN | 10.94 | 1.36 | 0.013629 | 13.42 | 3.08 | 0.000016 |
| ENO3 | 10.06 | -2.23 | 0.001120 | 12.35 | -0.69 | 0.009636 |
| ENPP2 | 9.12 | -1.91 | 0.004048 | 12.99 | -0.41 | 0.016366 |
| EPAS1 | 14.26 | -2.59 | 0.000410 | 13.14 | -0.64 | 0.014242 |
| FAM154B | 7.77 | -1.06 | 0.033605 | 10.21 | -0.36 | 0.035670 |
| FAM20C | 12.84 | 0.87 | 0.022157 | 10.69 | 1.44 | 0.003179 |
| FSTL1 | 17.14 | 1.03 | 0.017730 | 15.16 | 1.96 | 0.000004 |
| GGCX | 10.73 | -0.73 | 0.029469 | 10.70 | -0.96 | 0.001969 |
| GGT5 | 7.34 | -0.67 | 0.044755 | 8.16 | -0.93 | 0.001706 |
| GINS1 | 12.36 | 1.10 | 0.013368 | 9.69 | 1.32 | 0.000611 |
| GINS2 | 12.70 | 0.67 | 0.030172 | 8.98 | 1.71 | 0.000083 |
| GPR20 | 9.21 | 2.02 | 0.000580 | 7.07 | 0.95 | 0.005075 |
| GSTA4 | 12.11 | -0.72 | 0.016022 | 11.55 | -0.61 | 0.017292 |
| HDAC6 | 10.63 | 1.53 | 0.000478 | 10.86 | 0.63 | 0.002000 |
| HES6 | 11.29 | 1.53 | 0.016007 | 12.56 | 0.73 | 0.001583 |
| HIP1R | 12.29 | -1.05 | 0.007774 | 9.86 | -0.68 | 0.000640 |
| HMGN3 | 12.82 | 0.69 | 0.048957 | 11.26 | 0.72 | 0.006134 |
| INCENP | 10.04 | 1.31 | 0.036223 | 12.30 | 1.14 | 0.000272 |
| ING4 | 10.38 | -1.08 | 0.008163 | 12.66 | -0.41 | 0.021327 |
| IQSEC2 | 11.60 | -1.04 | 0.009299 | 12.44 | -0.48 | 0.022345 |
| KCNN2 | 12.06 | -1.80 | 0.002929 | 8.47 | -1.22 | 0.009402 |
| KCTD14 | 7.07 | -0.95 | 0.039884 | 9.80 | -0.61 | 0.031442 |
| KIT | 9.66 | -0.99 | 0.027587 | 13.12 | -0.88 | 0.012905 |
| LBH | 10.31 | 1.56 | 0.002584 | 12.87 | 0.72 | 0.002351 |
| LPCAT2 | 9.25 | 0.98 | 0.037453 | 7.60 | 0.90 | 0.008390 |
| LRRFIP1 | 11.97 | -0.66 | 0.037696 | 13.27 | -0.54 | 0.007590 |
| MAGI1 | 7.52 | -1.21 | 0.024711 | 9.03 | -0.51 | 0.049757 |
| MAP3K11 | 13.29 | -2.78 | 0.000346 | 11.58 | -0.56 | 0.012199 |
| MAP3K5 | 11.98 | -1.80 | 0.006828 | 10.00 | -0.87 | 0.006964 |
| MBOAT1 | 9.75 | 1.31 | 0.000828 | 12.08 | 0.42 | 0.014647 |
| MDGA1 | 9.91 | 1.90 | 0.007768 | 8.10 | 1.57 | 0.005156 |
| MEGF6 | 9.82 | -0.87 | 0.030507 | 12.14 | -1.07 | 0.000602 |
| NBL1 | 14.18 | -0.93 | 0.043617 | 15.72 | -0.47 | 0.022266 |
| NCBP2 | 11.40 | -1.97 | 0.000828 | 10.18 | -0.45 | 0.014578 |
| NEDD4L | 10.41 | -1.28 | 0.045937 | 9.60 | -1.68 | 0.001422 |
| NHLRC3 | 8.90 | 1.69 | 0.008096 | 8.34 | 0.95 | 0.000194 |
| NIPAL3 | 13.11 | -1.34 | 0.001092 | 13.13 | -1.10 | 0.001203 |
| NUBP1 | 13.19 | 0.79 | 0.038434 | 13.00 | 0.98 | 0.000249 |
| NUSAP1 | 12.38 | 0.87 | 0.019359 | 9.80 | 1.60 | 0.001794 |
| ODF3B | 7.27 | -0.90 | 0.025859 | 13.09 | -0.69 | 0.015031 |
| OTUD3 | 9.22 | 1.70 | 0.012549 | 11.10 | 0.72 | 0.009897 |
| PMEPA1 | 10.80 | 0.86 | 0.013635 | 13.06 | 1.49 | 0.000062 |
| PNKD | 7.12 | -1.15 | 0.005612 | 7.96 | -1.08 | 0.003491 |
| PNPLA6 | 9.56 | -1.63 | 0.009919 | 12.37 | -0.30 | 0.047030 |
| POMGNT1 | 13.66 | -0.88 | 0.005364 | 12.08 | -0.32 | 0.023190 |
| PPFIBP2 | 8.82 | -0.75 | 0.032615 | 13.05 | -0.80 | 0.015070 |
| PPP2R3C | 13.91 | 0.86 | 0.033926 | 11.45 | 0.53 | 0.025985 |
| PRC1 | 14.41 | 0.84 | 0.004102 | 11.18 | 1.78 | 0.000124 |
| PRELID2 | 9.33 | 0.99 | 0.042979 | 9.99 | 0.56 | 0.005315 |
| PRICKLE1 | 11.41 | -1.22 | 0.005224 | 14.07 | -1.42 | 0.000180 |
| PRMT2 | 10.05 | -0.66 | 0.047910 | 14.01 | -0.67 | 0.030405 |
| PRPS2 | 8.02 | -0.95 | 0.041219 | 10.22 | -0.91 | 0.000555 |
| R3HDM2 | 12.36 | -1.23 | 0.019502 | 13.32 | -0.42 | 0.017910 |
| RALGAPB | 10.47 | -1.18 | 0.023239 | 12.96 | -0.47 | 0.009014 |
| RASGRP2 | 8.06 | -1.37 | 0.009020 | 12.24 | -0.60 | 0.004782 |
| RBM24 | 9.47 | -2.59 | 0.000360 | 9.44 | -0.27 | 0.039708 |
| REEP5 | 13.48 | -2.17 | 0.002005 | 12.55 | -0.42 | 0.025985 |
| RGMB | 9.81 | -0.75 | 0.028634 | 11.30 | -0.87 | 0.005668 |
| RNASEH2A | 13.07 | 0.75 | 0.036633 | 10.81 | 0.49 | 0.048594 |
| RNF141 | 11.52 | -1.54 | 0.015631 | 12.99 | -0.67 | 0.001122 |
| ROBO4 | 7.54 | -1.65 | 0.011311 | 14.51 | -0.67 | 0.011915 |
| RRM2B | 9.58 | -2.97 | 0.001651 | 8.51 | -0.62 | 0.006787 |
| RTCD1 | 14.33 | 1.38 | 0.004627 | 12.19 | 0.52 | 0.007499 |
| SELENBP1 | 12.64 | -0.81 | 0.016828 | 17.28 | -0.45 | 0.040980 |
| SGIP1 | 8.80 | -1.31 | 0.012026 | 8.65 | -0.62 | 0.019749 |
| SH3D19 | 12.72 | -1.29 | 0.008996 | 9.60 | -0.91 | 0.013287 |
| SIRT5 | 10.40 | -1.13 | 0.011311 | 11.21 | -0.51 | 0.013278 |
| SKA1 | 9.70 | 1.02 | 0.006580 | 8.56 | 1.79 | 0.000316 |
| SLC2A6 | 11.76 | 0.89 | 0.014990 | 10.92 | 0.71 | 0.019866 |
| SLC39A14 | 13.51 | 0.96 | 0.017472 | 11.15 | 2.02 | 0.000197 |
| SLC40A1 | 8.90 | 1.32 | 0.021215 | 10.64 | 0.92 | 0.000270 |
| SMARCC2 | 14.23 | -0.64 | 0.032899 | 9.83 | -0.54 | 0.018426 |
| SMS | 15.75 | -1.76 | 0.017748 | 10.87 | -0.87 | 0.000163 |
| SMURF2 | 8.88 | -1.23 | 0.013799 | 10.89 | -0.83 | 0.000156 |
| SOCS1 | 13.01 | 1.67 | 0.000297 | 10.87 | 0.60 | 0.009453 |
| SORL1 | 9.72 | -1.50 | 0.000718 | 11.34 | -1.06 | 0.002206 |
| SPC25 | 11.97 | 1.35 | 0.015518 | 11.39 | 2.26 | 0.000033 |
| SPON2 | 15.60 | -0.80 | 0.025019 | 12.53 | -1.24 | 0.011622 |
| SREBF2 | 11.51 | 1.25 | 0.001645 | 10.32 | 1.35 | 0.000207 |
| STX3 | 8.69 | -1.21 | 0.024221 | 8.72 | -1.13 | 0.000179 |
| SYNGR1 | 9.53 | -1.37 | 0.008975 | 7.11 | -0.46 | 0.043806 |
| TCEAL1 | 11.86 | -0.91 | 0.018668 | 10.23 | -0.63 | 0.041974 |
| TDG | 13.68 | 0.99 | 0.033435 | 8.30 | 0.43 | 0.029690 |
| TGFB3 | 8.06 | 1.00 | 0.010858 | 13.52 | 0.81 | 0.007305 |
| TGFBR1 | 8.50 | 1.74 | 0.007822 | 12.40 | 0.56 | 0.005692 |
| THBD | 7.77 | -1.61 | 0.012078 | 15.29 | -1.22 | 0.034879 |
| THBS3 | 10.56 | -0.67 | 0.030059 | 13.75 | -1.70 | 0.000072 |
| TMEM159 | 12.32 | -1.33 | 0.001835 | 12.14 | -0.46 | 0.046713 |
| TMEM164 | 11.32 | -1.95 | 0.000495 | 13.69 | -0.49 | 0.020506 |
| TMEM20 | 7.65 | 0.85 | 0.027311 | 9.65 | 0.61 | 0.024024 |
| TMEM45A | 12.57 | 1.09 | 0.027911 | 11.66 | 1.14 | 0.002854 |
| TRAIP | 11.29 | 1.01 | 0.039309 | 8.71 | 1.42 | 0.005390 |
| TRAK1 | 11.44 | -1.45 | 0.032442 | 7.15 | -0.33 | 0.034043 |
| TSPAN13 | 7.88 | -1.12 | 0.045464 | 15.27 | -1.74 | 0.000216 |
| TST | 14.12 | -2.01 | 0.000099 | 12.60 | -0.86 | 0.001296 |
| TTC39C | 8.78 | 0.82 | 0.025257 | 10.00 | 1.44 | 0.017367 |
| UBE2V1 | 13.50 | 2.19 | 0.000423 | 10.54 | 0.40 | 0.038237 |
| WASF1 | 12.40 | 0.65 | 0.031618 | 8.51 | 0.41 | 0.049891 |
